# The health, cost and equity impacts of restrictions on the advertisement of high fat, salt and sugar products across the transport for London network: a health economic modelling study

**DOI:** 10.1186/s12966-022-01331-y

**Published:** 2022-07-27

**Authors:** Chloe Thomas, Penny Breeze, Steven Cummins, Laura Cornelsen, Amy Yau, Alan Brennan

**Affiliations:** 1 School for Health and Related Research, University of Sheffield, 30 Regent Court, Regent Street, Sheffield, S1 4DA UK; 2 Department of Public Health, Environments and Society, London School of Hygiene and Tropical Medicine, 15-17 Tavistock Place, London, WC1H 9SH UK

**Keywords:** Diet, Obesity, High fat, salt and sugar (HFSS) products, Dietary policy, Advertising, Advertising restrictions, Health economic modelling

## Abstract

**Background:**

Policies aimed at restricting the marketing of high fat, salt and sugar products have been proposed as one way of improving population diet and reducing obesity. In 2019, Transport for London implemented advertising restrictions on high fat, salt and sugar products. A controlled interrupted time-series analysis comparing London with a north of England control, suggested that the advertising restrictions had resulted in a reduction in household energy purchases. The aim of the study presented here was to estimate the health benefits, cost savings and equity impacts of the Transport for London policy using a health economic modelling approach, from an English National Health Service and personal social services perspective.

**Methods:**

A diabetes prevention microsimulation model was modified to incorporate the London population and Transport for London advertising intervention. Conversion of calorie to body mass index reduction was mediated through an approximation of a mathematical model estimating weight loss. Outcomes gathered included incremental obesity, long-term diabetes and cardiovascular disease events, quality-adjusted life years, healthcare costs saved and net monetary benefit. Slope index of inequality was calculated for proportion of people with obesity across socioeconomic groups to assess equity impacts.

**Results:**

The results show that the Transport for London policy was estimated to have resulted in 94,867 (4.8%) fewer individuals with obesity, and to reduce incidence of diabetes and cardiovascular disease by 2,857 and 1,915 cases respectively within three years post intervention. The policy would produce an estimated 16,394 additional quality-adjusted life-years and save £218 m in NHS and social care costs over the lifetime of the current population. Greater benefits (e.g. a 37% higher gain in quality-adjusted life-years) were expected to accrue to individuals from the most socioeconomically deprived groups compared to the least deprived.

**Conclusions:**

This analysis suggests that there are considerable potential health and economic gains from restricting the advertisement of high fat, salt and sugar products. The population health and economic impacts of the Transport for London advertising restrictions are likely to have reduced health inequalities in London.

**Supplementary Information:**

The online version contains supplementary material available at 10.1186/s12966-022-01331-y.

## Background

Obesity is an increasingly important global problem. According to the Global Burden of Disease Study, the prevalence of obesity has continually increased in most countries since 1980 [1], and number of deaths attributed to high BMI has more than doubled between 1990 and 2017 [2]. Obesity is associated with higher risk of many diseases including type 2 diabetes, cardiovascular disease (CVD), chronic kidney disease, many cancers, and musculoskeletal problems such as osteoarthritis and chronic back pain [1], in addition to higher risk of poorer outcomes from COVID-19 [3], and impacts on mental health [4]. In England, 27% of men and 29% of women were estimated to be living with obesity in 2019, with a further 41% of men and 31% of women being overweight [5]. Obesity shows extensive socioeconomic patterning, with women in the most deprived quintile of the English population having almost double the rate of obesity compared with women in the least deprived quintile [5]. Socioeconomic differences are even more marked in children, where the most deprived children of primary school age have over double the rate of obesity and 4–5 times the rate of severe obesity than the least deprived [6].

Many different public health interventions have been proposed to tackle rising obesity rates and their consequences, ranging from weight loss interventions targeted specifically at individuals with obesity, to dietary policies that aim to reduce consumption of calories, fat, sugar and/or salt at the population level either through taxation, reformulation or labelling [7]. Another population level approach is to restrict advertising for particular products. It has been demonstrated that children eat more calories after watching advertising for high fat, sugar and salt (HFSS) products [8], and several studies have modelled the potential health benefits for children of restricting television advertising of junk food [9–11], indicating that substantial reductions in childhood obesity may be achieved through such methods.

In 2019, Transport for London (TfL) implemented restrictions on advertisements of HFSS food on its transport network [12]. One of the stated aims was to reduce advertising to children and hence address childhood obesity; however, there was potential for this intervention to also act on adult behaviour. A controlled interrupted time series analysis based on data from 1,970 households in the Kantar Fast Moving Consumer Goods panel showed that the intervention led to changes in household purchases of HFSS food equivalent to a 6.7% (95% CI: 3.2% to 10.1%) reduction in calories compared to the North of England control [13], together with associated reductions in purchases of fat, saturated fat and sugar. The other stated aim was to reduce inequalities in obesity and health outcomes related to obesity, and there was some indication of greater reductions in purchase of HFSS products, and hence potentially reductions in calorie intake, in households of middle or low socioeconomic status compared to those of high socioeconomic status.

Health economic modelling enables medium to long term benefits of health policies to be predicted, providing evidence to inform decisions about future implementation. In this analysis we use a microsimulation modelling approach to assess the potential health benefits, cost savings and equity impacts of the TfL advertising intervention on adults compared to no intervention.

## Methods

### Model background

This analysis was undertaken by adapting a pre-existing health economic model: the School for Public Health Research (SPHR) diabetes prevention model version 4, which has previously been used to evaluate a variety of different diabetes prevention and weight loss interventions [14–17]. The model is an individual patient level microsimulation model built in R software, with annual cycles and a lifetime horizon, which takes an English National Health Service (NHS) and Personal Social Services (PSS) perspective. It simulates the life course of individuals aged 16 and over from Health Survey for England (HSE) 2014 [18], representing the adult population of England. The model is unable to simulate the life course for children aged under 16 due to the differing data requirements for this population, thereby limiting its use to modelling intervention impacts on adults. Over their lifetime, modelled individuals may develop diabetes, hypertension, cardiovascular disease, heart failure, microvascular complications of diabetes, dementia, breast and bowel cancer, osteoarthritis or depression. Disease risk is driven by personalised trajectories of correlated metabolic risk factors including BMI, systolic blood pressure (SBP), cholesterol and HbA1c. Diagnosis and treatment of diseases is associated with costs to the NHS and social care system, and disease health states are associated with reduction in health-related quality of life. A full description of the model methodology, parameters and assumptions can be found in the supplementary materials.

### Population

The analysis estimates effects on the population aged 16 and over of the Greater London area (*n* = 7,149,281 based on UK mid-year estimates for 2020) [19]. The baseline population in the SPHR model is comprised of individuals aged 16 and over from HSE 2014 (*n* = 8077). HSE 2014 contains a set of survey weights that enable the sample to be representative of England by either increasing or reducing the importance of each individual. The modelled population was reweighted to reflect the sociodemographic characteristics of the Greater London area. Sets of survey weights representing each local authority (LA) in England were developed using two dimensional iterative proportional fitting of local data about age, sex, ethnicity and deprivation quintile. Age, sex and ethnicity data came from the 2011 Census [20], whilst deprivation data came from the 2015 English Indices of Deprivation [21]. The greatest possible number of population demographic characteristic breakdowns was used to give the best possible fit to LA populations (16 age groups, two sex groups, three ethnic groups [white, Asian and other] and five deprivation groups [Index of Multiple Deprivation IMD quintiles]). Weights from the 33 local authority areas that make up the Greater London area were summed for each individual, and data normalised to reflect the most recent estimates of population size (7,149,281) [19]. Baseline characteristics for the reweighted model population are summarised in Table 1, with additional obesity-related summary statistics for IMD quintiles shown in Supplementary Table 1.

**Table 1:** Summary statistics for the modelled baseline population, based on sampling of 100,000 individuals aged 16 and over from the Health Survey for England 2014 [18], reweighted to reflect the age, sex, ethnicity and socioeconomic distributions of Greater London

Characteristic	Mean	Standard Deviation
Age (years)	43.1	17.6
Body mass index (kg/m^2^)	27.3	5.6
Systolic blood pressure (mm Hg)	123.5	16.3
Total cholesterol (mmol/L)	5.02	1.07
Glycated Haemoglobin (HbA1c, %)	5.61	0.90
	**Percentage of Population**	**Number Greater London**
Male	48.8%	3,488,206
White ethnicity	63.5%	4,543,225
Asian ethnicity	14.5%	1,035,859
Other ethnicity	18.5%	1,324,190
NS-SEC: High (least deprived)	31.4%	2,245,232
NS-SEC: Mid	51.6%	3,691,817
NS-SEC: Low (most deprived)	17.0%	1,212,232
IMD1 (least deprived)	10.2%	726,510
IMD2	15.8%	1,127,084
IMD3	21.2%	1,515,719
IMD4	30.7%	2,193,542
IMD5 (most deprived)	22.2%	1,586,425
Obese	27.4%	1,957,963
Overweight	38.0%	2,713,470

*NS-SEC* National Statistics Socioeconomic Classification, *IMD* Index of multiple deprivation

### Intervention

The intervention modelled the impact of changes in calorie purchase following advertising restrictions across the TfL network for high fat, salt and sugar (HFSS) products, compared to a scenario in which no advertising restrictions were implemented and no change in purchasing or consumption of calories took place. A previously published study used a controlled interrupted time series analysis of data from the Kantar Fast Moving Consumer Goods panel to assess the impact of the TfL intervention on household purchasing [13]. The study randomly selected 1,970 households from London and the North of England (with households having an average of 2.6 individuals), each of whom recorded all food and drink items purchased and brought into the home. Kantar collects data about number of packs purchased of different food types, and directly measures nutritional data twice per year. Products were scored as HFSS or not according to the Nutrient Profiling Model, which had been used to determine advertising restrictions for the TfL intervention [22]. A two-part logit-generalised linear model was fitted to firstly estimate the probability of purchasing a product, and if so, to determine how much product was purchased. The study found that purchases of HFSS food increased over time in both locations. However; following the TfL intervention in London, relative purchases of energy decreased by an average of 1,001 (95% CI: 456 to 1,546) calories per household each week compared to the counterfactual, which was constructed using the pre-intervention trend in London and incorporating the changes seen in the North of England (to account for seasonal and secular changes common to both areas). As household purchases are made by adults, no information was available about potential differences in HFSS purchase or consumption by children. The findings also showed that reductions in purchased energy tended to be lower if the main food shopper was of high socioeconomic status (SES) (Table 2), with socioeconomic position classified according to the National Readership Survey (NRS) occupational social grade classification (A, B, C1, C2, D, E) [23], and distributed into three groups: high (AB), middle (C1C2) and low (DE).

**Table 2:** Intervention effects applied in the basecase and sensitivity analysis scenarios, based on household weekly calorie reduction from the TfL study [13]

Population		Household reduction in calories per week (data)	Individual reduction in calories per day (model)	Mean individual reduction during first 12 months			
				**BMI (kg/m**^**2**^**)**	**SBP** **(mm Hg)**	**Total Chol (mmol/l)**	**HbA1c (%)**
**Basecase**	NS-SEC: High	586.6	32.2	0.295	0.676	0.0285	0.00273
	NS-SEC: Mid	1139.4	62.6	0.575	1.316	0.0555	0.00524
	NS-SEC: Low	875.5	48.1	0.470	1.068	0.0452	0.00406
	IMD1	N/A	46.8	0.430	0.986	0.0418	0.00407
	IMD2	N/A	47.5	0.438	0.995	0.0424	0.00407
	IMD3	N/A	50.3	0.464	1.058	0.0447	0.00414
	IMD4	N/A	51.2	0.477	1.095	0.0460	0.00430
	IMD5	N/A	54.0	0.503	1.150	0.0484	0.00452
	**Mean**	**1001.0**	**50.6**	**0.469**	**1.073**	**0.0453**	**0.00425**
**SA1**	NS-SEC: High	N/A	55.0	0.483	1.107	0.0467	0.00449
	NS-SEC: Mid	N/A	55.0	0.496	1.133	0.0479	0.00449
	NS-SEC: Low	N/A	55.0	0.506	1.150	0.0486	0.00433
	IMD1	N/A	55.0	0.489	1.117	0.0474	0.00461
	IMD2	N/A	55.0	0.490	1.115	0.0474	0.00454
	IMD3	N/A	55.0	0.491	1.121	0.0474	0.00438
	IMD4	N/A	55.0	0.496	1.137	0.0479	0.00446
	IMD5	N/A	55.0	0.497	1.133	0.0478	0.00444
	**Mean**	**1001.0**	**55.0**	**0.493**	**1.127**	**0.0476**	**0.00446**
**SA2**	**Mean**	**1001.0**	**50.6**	**0.493**	**0**	**0**	**0**
**SA3**	**Mean**	**500.5**	**25.3**	**0.230**	**0.526**	**0.0222**	**0.00208**
**SA4**	**Mean**	**1001.0**	**50.6**	**0.469**	**1.073**	**0.0453**	**0.00425**
**SA5**	**Mean**	**1001.0**	**50.6**	**0.469**	**1.073**	**0.0453**	**0.00425**

*SES* Socioeconomic status, *BMI* Body mass index, *SBP* Systolic blood pressure, *Chol* Cholesterol, *HbA1c* Glycated haemoglobin, *SA* Sensitivity analysis, *SA1* No socioeconomic gradient in calorie input, *SA2* No indirect metabolic effects, *SA3* Half calorie reduction, *SA4* 3 year duration of effect, *SA5* 1 year return to baseline BMI

In our analysis it was assumed that reductions in weekly calorie purchase could be directly equated with reductions in weekly calorie consumption. This assumption was supported by two pieces of evidence. Firstly; the TfL study found no substitutions of HFSS to non HFSS foods suggesting that calories are not compensated for elsewhere [13]. Secondly, surveys of household food wastage suggest that HFSS foods are only infrequently wasted compared to other foods (e.g. only about 5% wastage for confectionary and snacks) [24]. It was further assumed that weekly household calorie reductions were evenly divided between days of the week and the average number of individuals in the household to obtain a per person daily reduction of 55 cal. Whilst trial follow-up was 10 months, the SPHR model used annual cycles, so it was assumed the calorie reduction was maintained for a further two months. Conversion of reduction in calories to change in weight was based upon an approximation of the mathematical model developed by Hall et al. based on the National Institute of Diabetes and Digestive and Kidney Diseases (NIDDKD) Body Weight Planner online tool [25, 26]. This took daily reduction in calories over the 12 month period as an input to estimate change in weight at 12 months. A dataset of 1000 outputs from the Body Weight Planner tool was extracted to estimate the impact of changes in calories, % carbohydrate and sodium after 12 months of a sustained dietary change. An ordinary least squares regression model was specified to describe the changes in calories, percentage carbohydrate, sodium and how these are modified by baseline weight, age and sex. Goodness of fit was assessed using the R^2^ and Akaike Information Criterion. Full methods and regression coefficients can be found in Sect. 11 of the supplementary technical appendix.

For the TfL intervention, per person changes in daily calories for each SES group were converted to change in weight at 12 months, assuming no change in % carbohydrate or sodium intake (Table 2). NRS socioeconomic classifications were not available within HSE and so could not be directly mapped onto the model baseline population. HSE does have National Statistics Socioeconomic Classification (NS-SEC) groupings [27]. These were distributed into three SES groups: high (higher managerial, lower managerial and professional), middle (intermediate occupations, small employers and own account, lower supervisory and technical, semi-routine and other), and low (routine, never worked and long-term unemployed), and these categories were assumed to align with the three NRS groups to enable intervention effectiveness to be stratified by SES.

Each modelled individual has a personal height measurement, enabling 12 month BMI change to be calculated at the individual level. Whilst there was no direct information about changes to SBP, cholesterol or HbA1; in the basecase scenario indirect impacts of BMI on these metabolic factors were included based on a statistical analysis of the Whitehall II cohort (Table 2) [28]. There is no evidence about the long-term effect of the policy. In the basecase scenario it was assumed that the TfL intervention was effective in reducing calorie consumption for the year of the intervention only and that the calorie intake of individuals would return to the level prior to the intervention after that first year. The Body Weight Planner Tool was used to assume that this would result in a return to baseline BMI by four years following the intervention [25], with this time frame also supported by evidence from long-term follow-up of structured weight loss interventions [29]. It was assumed that no costs were incurred as a result of the TfL intervention, as there was not found to be any reduction in advertising revenue as a consequence of the intervention [13], nor were any costs to the NHS or social care incurred in implementing the intervention.

Five sensitivity analyses were carried out to investigate structural uncertainties around intervention effectiveness. In the first sensitivity analysis (SA1), socioeconomic differences in calorie reduction were removed and instead all individuals were given the mean calorie reduction. In SA2, the indirect effects of calorie reduction on SBP, cholesterol and HbA1c were removed so only the direct impacts on BMI would contribute to the outcomes. In SA3, the calorie reduction was halved to represent a lower effectiveness scenario. In SA4, it was assumed that calorie intake and BMI reductions would endure at the same level for three years rather than just one year, before BMI returned to the level prior to intervention over the subsequent four years. In SA5 it was assumed that the BMI reductions would endure one year as in the basecase, but then return to the level prior to intervention in the subsequent year.

### Outcomes

Model outcomes included lifetime costs and quality-adjusted life years (QALYs), new type 2 diabetes cases and cardiovascular disease (CVD) events throughout the 20 years following intervention implementation, and the proportion of the population obese or overweight immediately following intervention implementation. Lifetime net monetary benefit was calculated using both £20,000 and £30,000 as the monetary values of a QALY [30]. Unit health and social care costs were based on 2020 UK pounds (£). Costs and QALYs were discounted at 3.5% as recommended by NICE [30]. Incremental analysis was performed by comparing basecase and sensitivity analysis scenarios against a no intervention control. Results were collected for the entire population of London, and per 100,000 individuals in each IMD quintile. A graphical representation of cross-tabulation between IMD quintiles and the three NS-SEC groups used in the modelling can be seen in Supplementary Fig. 1. An inequalities analysis was carried out to determine whether the intervention had reduced inequity across IMD quintiles, using proportion obese as the outcome of interest, and the slope index of inequalities (which is commonly used to assess public health inequalities in England [31]) as the measure [32].

The model was run using probabilistic sensitivity analysis (PSA) to enable an accurate estimation of mean values, taking model non-linearity into account, and to enable uncertainty around model outcomes to be estimated using 95% credible intervals. For each of the basecase and the four structural sensitivity analysis scenarios, 1,000 PSA loops were run, each comprising 100,000 sampled individuals.

## Results

### Basecase analysis

**Fig. 1:**
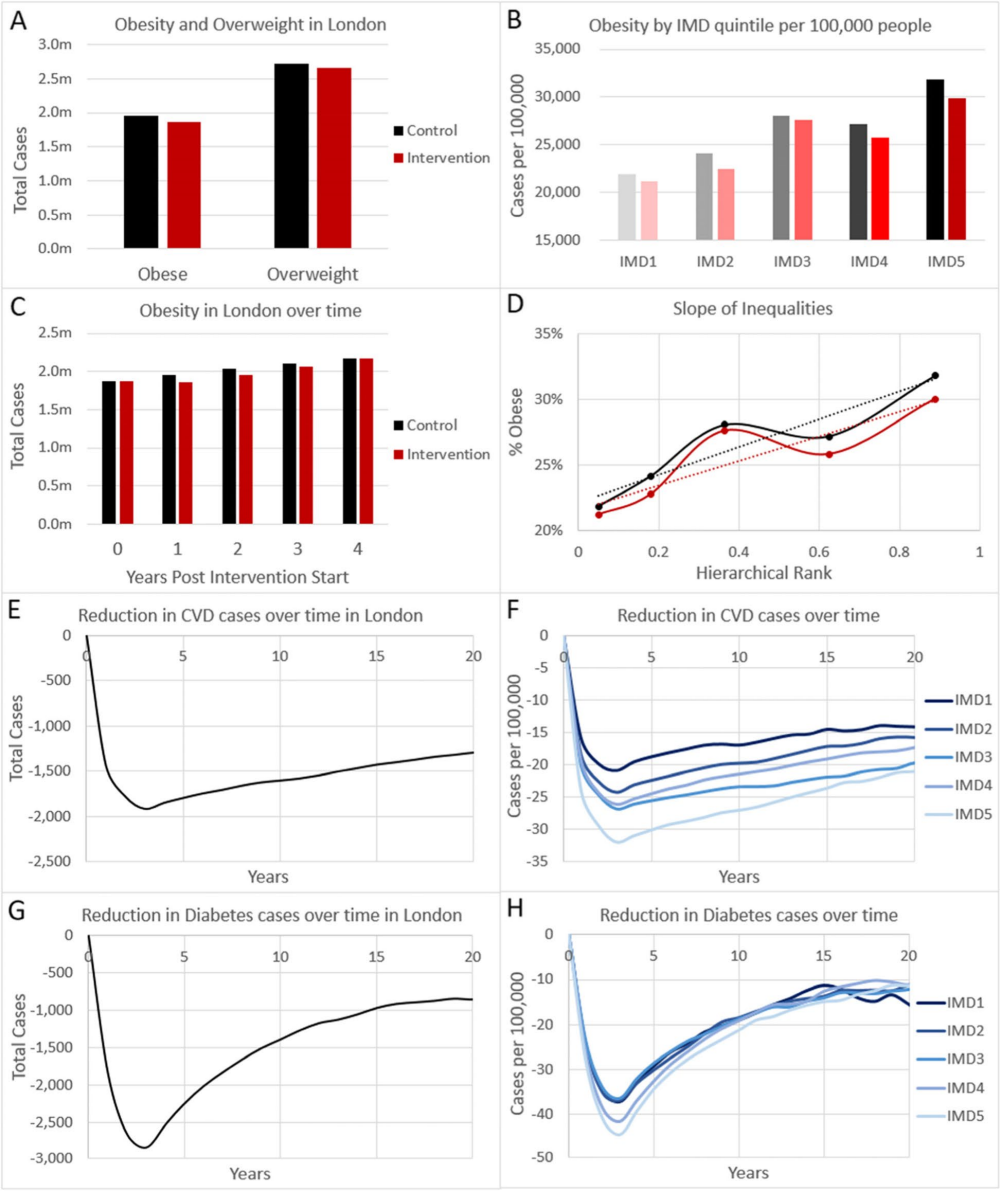
Modelled reduction in disease outcomes expected with the Transport for London intervention. Expected number of cases of obesity and overweight at one year with (red bars) and without (black bars) intervention in **A** the whole of London, **B** per 100,000 people of each IMD quintile. **C** Obesity over time in London with (red) and without (black) intervention. **D** Slope of inequalities for obesity across IMD quintiles for intervention (red) and control (black) scenarios, with data shown as solid line and linear slope shown as dotted line. Expected reduction of **E**–**F**) cardiovascular disease (CVD) cases, or **G**-**H**) new type 2 diabetes cases, in **E**,**G**) the whole of London, **F**,**H**) per 100,000 people of each IMD quintile, over a 20 year time horizon

The modelling suggests that there were 1.96 million obese and 2.71 million overweight individuals in Greater London prior to intervention implementation (Table 1). Twelve months after implementation of the TfL intervention, the model estimates that 94,867 (-4.8%) fewer people would be expected to be obese and 49,145 (-1.8%) fewer people would be expected to be overweight, compared to projected increases in obesity in the no intervention control (Panels A & C, Fig. 1). Each IMD quintile group shows reductions in obesity, and, whilst there is no visual trend in obesity reduction by IMD quintile (Panel B, Fig. 1), the slope of inequalities for proportion of obese was slightly lower in the intervention arm (0.2155) than in the control arm (0.2218 indicating that there was a greater reduction in obesity-among people living in more deprived areas, and hence a small reduction in health inequity due to the intervention.

In the longer term, a reduction in diabetes diagnoses and CVD cases is expected, peaking around three years after policy implementation at 2,857 fewer diabetes cases and 1915 fewer CVD cases in the Greater London area. Subsequently cases are expected to rise as individuals experience delayed onset of these diseases. These health benefits at three years post policy implementation also show a clear socioeconomic gradient with the most deprived IMD quintiles tending to have a more extreme reduction in diabetes and CVD cases than the less deprived quintiles (Panels F & H, Fig. 1). Whilst this socioeconomic gradient persists over time for CVD cases, for diabetes cases it starts to reverse from about ten years following intervention implementation, indicating a greater tendency to experience later onset of diabetes delayed by intervention in the most deprived groups.

It is predicted that the expected reduction in obesity and related diseases due to the TfL intervention would result in a total NHS and PSS cost saving of £218 m (95% CI £49 m-£438 m) over the lifetime of the Greater London population, and a QALY gain of 16,394 (95% CI 990–36,951) (Table 3). The intervention has a high probability of saving costs and gaining QALYs, which can be observed by the distribution of points on the cost-effectiveness plane (Supplementary Fig. 2). Over half of the cost savings come from osteoarthritis and CVD prevention, which account for about 29% and 27% of the total respectively (Supplementary Table 2). The incremental net monetary benefit at the £20,000 value of a QALY was £546 m (95% CI £125 m-£1,088 m). In line with the long-term disease reductions; costs saved, QALYs gained and incremental net monetary benefits tend to show a gradient where they are highest in the most deprived socioeconomic quintiles (Table 3). Note that IMD subgroup results are more uncertain than those for the population average due to the smaller numbers of modelled individuals in each subgroup compared to the total population.

**Table 3:** Incremental cost-effectiveness results for the Transport for London intervention (basecase scenario) compared with no intervention. All outcomes are accumulated over a lifetime horizon. Cost savings are shown as negative values

Outcomes (incremental)		Mean	Lower 95% CI	Upper 95% CI
**TOTAL NHS & PSS COSTS**				
Total Population Greater London		-£218,703,431	-£437,582,367	-£48,711,680
Per 100,000	IMD1 (least deprived)	-£2,485,362	-£8,178,280	£2,745,347
	IMD2	-£2,658,155	-£7,725,675	£2,070,542
	IMD3	-£3,244,109	-£7,773,152	£804,521
	IMD4	-£2,992,482	-£7,084,304	£292,685
	IMD5 (most deprived)	-£3,455,994	-£8,368,168	£833,012
**QALYs**				
Total Population Greater London		16,394	990	36,951
Per 100,000	IMD1 (least deprived)	187	-315	677
	IMD2	199	-203	656
	IMD3	242	-110	646
	IMD4	230	-81	589
	IMD5 (most deprived)	257	-90	673
**NET MONETARY BENEFIT (£20,000 per QALY THRESHOLD)**				
Total Population Greater London		£545,591,744	£125,122,911	£1,088,464,368
Per 100,000	IMD1 (least deprived)	£6,221,094	-£5,151,238	£18,712,669
	IMD2	£6,642,525	-£3,419,224	£17,763,377
	IMD3	£8,088,383	-£559,234	£18,357,648
	IMD4	£7,589,376	-£23,340	£16,927,201
	IMD5 (most deprived)	£8,593,878	-£231,314	£18,975,049
**NET MONETARY BENEFIT (£30,000 per QALY THRESHOLD)**				
Total Population Greater London		£709,535,901	£139,093,456	£1,445,703,565
Per 100,000	IMD1 (least deprived)	£8,088,960	-£8,422,256	£24,890,241
	IMD2	£8,634,710	-£4,968,504	£23,748,141
	IMD3	£10,510,520	-£1,614,656	£24,104,387
	IMD4	£9,887,824	-£784,264	£22,131,660
	IMD5 (most deprived)	£11,162,820	-£1,062,051	£25,375,454

*NHS* National Health Service, *PSS* Personal Social Services, *QALY* Quality-adjusted Life Year, *IMD* Index of Multiple Deprivation, *CI* Credible Interval

### Sensitivity analyses

**Fig. 2:**
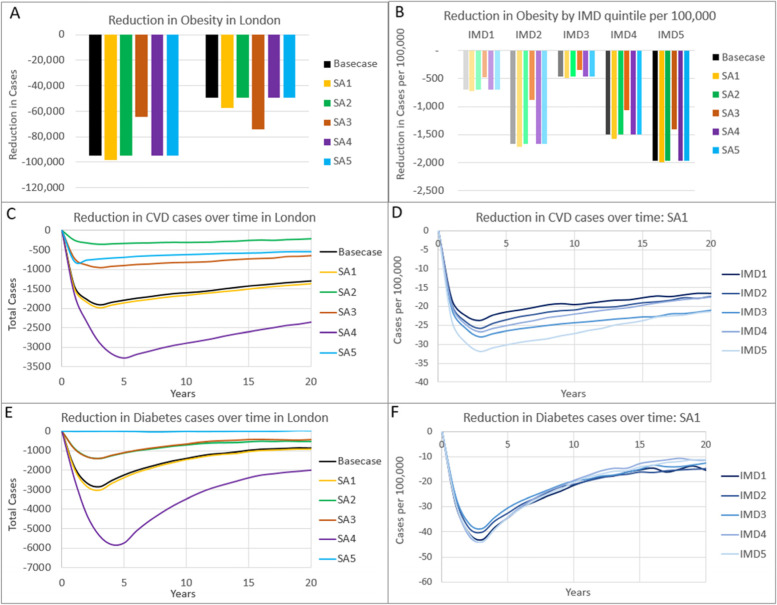
Expected incremental reduction in 12-month obesity (**A**-**B**), cardiovascular disease cases (**C**-**D**) and new type 2 diabetes cases (**E**–**F**), with different sensitivity analyses. **A**,**C**,**E** Results for the whole of London comparing basecase and all four sensitivity analysis scenarios. B) Results per 100,000 people of each IMD quintile with different sensitivity analyses. **D**,**F** Results per 100,000 people of each IMD quintile for sensitivity analysis SA1: no socioeconomic gradient in calorie reduction. SA2 no indirect metabolic effects; SA3 half calorie reduction; SA4 3 year duration of effect; SA5 1 year return to baseline BMI

All four sensitivity analysis results confirm that the TfL intervention is likely to reduce obesity overall and reduce the slope of inequalities for the proportion obese, reduce CVD and diabetes cases, save costs and produce QALYs and net monetary benefit, although the absolute magnitude and statistical significance of these values changes depending upon the sensitivity analysis (Table 4, Fig. 2 and Supplementary Table 3). Overall, SA1: assuming each socioeconomic subgroup experiences the population average, produces slightly greater benefits than the basecase analysis. This is because the average calorie reduction in SA1 is slightly higher than in the basecase because the modelled population has a lower proportion of individuals in the mid NS-SEC category than the intervention study sample. Socioeconomic gradients in long-term outcomes are still apparent in SA1 despite the calorie reduction being identical between subgroups (Table 2), suggesting that a substantial proportion of the difference in long-term outcomes between socioeconomic groups is due to the underlying poorer health and hence greater capacity for improvement within the more deprived subgroups.

**Table 4:** Comparison of incremental cost-effectiveness results for basecase and sensitivity analysis scenarios. All outcomes are accumulated over a lifetime horizon and are for the total population of Greater London. Cost savings are shown as negative values

Outcome	Mean	Lower 95% CI	Upper 95% CI
**TOTAL NHS & PSS COSTS**			
Basecase	-£217,703,431	-£437,582,367	-£48,711,680
SA1	-£226,718,104	-£475,058,585	-£37,773,383
SA2	-£151,531,984	-£333,771,635	-£5,927,436
SA3	-£107,991,545	-£255,351,553	£16,979,751
SA4	-£393,625,146	-£752,241,459	-£138,109,426
SA5	-£79,789,371	-£223,685,840	£40,117,017
**QALYs**			
Basecase	16,394	990	36,951
SA1	17,377	814	40,812
SA2	10,342	-4,268	27,952
SA3	8,026	-2,991	21,643
SA4	29,865	6,060	64,508
SA5	5,997	-5,139	17,036
**NET MONETARY BENEFIT (£20,000 per QALY THRESHOLD)**			
Basecase	£545,591,744	£125,122,911	£1,088,464,368
SA1	£574,259,454	£125,502,640	£1,199,502,240
SA2	£358,372,911	£11,893,203	£840,304,073
SA3	£268,518,915	£3,417,367	£602,086,044
SA4	£990,928,187	£336,178,002	£1,917,914,385
SA5	£199,739,062	-£49,302,909	£462,881,219

*QALY* Quality-adjusted life-year, *NMB* Net monetary benefit, *SA* Sensitivity analysis, *SA1* No socioeconomic gradient in calorie reduction, *SA2* No indirect metabolic effects, *SA3* Half calorie reduction, *SA4* 3 year duration of effect, *SA5* 1 year return to baseline BMI

The impact of removing indirect effects on other metabolic factors (SA2) indicates the relative importance of the intervention in reducing BMI compared to reducing other metabolic factors. Only about 350 CVD cases are prevented at three years post intervention compared with almost 2000 when indirect metabolic impacts are included, indicating the particular importance of blood pressure and cholesterol in CVD (Fig. 2). A reduction in diabetes cases by about 1,400 at three years post intervention is estimated for SA2, compared to 1,857 in the basecase, despite no change in HbA1c. This is because the probability of being tested and diagnosed with diabetes (mediated through the QDiabetes risk equation in the model [33]) is partly conditional upon BMI. However, despite the significantly lower prevention of CVD and diabetes disease cases, 63% of total QALY gains and 70% of cost savings are still accrued compared to the basecase scenario. This is because there are only small differences between scenarios in the costs saved and QALYs gained due to other modelled conditions that are related primarily to obesity and less to other metabolic factors, particularly the 29% of basecase cost savings related to osteoarthritis, but also for depression, breast and bowel cancers.

As expected, smaller calorie reductions (SA3), and shorter duration of effect (SA5) produce smaller reductions in long-term disease, cost-savings and QALY gains (and no significant reduction in diabetes cases estimated with SA5), whilst increasing the duration of effect of the calorie reduction (SA4) produces greater reductions in long-term disease, cost-savings and QALY gains. Whilst net monetary benefit varies in line with the benefits produced, all sensitivity analyses indicate a very high probability of being cost-saving (Table 4).

## Discussion

The TfL HFSS advertising policy is very likely to have reduced obesity and obesity-related disease, saved NHS and PSS costs, and produced QALYs and net monetary benefit compared to the no intervention control. Whilst there is considerable uncertainty within the effectiveness data, particularly in the assumption that calorie purchase equates to calorie consumption; the scenario analyses undertaken suggest that these conclusions are robust to both model parameter uncertainty and a range of different assumptions around magnitude and duration of intervention effect. Whilst other explanations for the difference in calorie purchase between the two study arms cannot be ruled out, the TfL analysis found that the purchase differences were specific for HFSS foods, and that greater effects were observed in people who used public transport regularly, suggesting that the advertising policy was indeed the reason for the difference [13]. Obesity has been increasing over time, and is projected to increase further both in England and elsewhere [34, 35], which is reflected in the modelling results presented here. In line with this, the published TfL evaluation indicated that purchase of HFSS products actually increased in both arms during the intervention period [13]; however, a smaller increase was found in the London arm exposed to the advertising restrictions compared with the North of England unexposed arm. This suggests that whilst advertising restrictions may have some role in slowing the growth in obesity at the population level, other strategies will be required if obesity levels are to be actively reduced.

A stated aim of the TfL advertising restrictions was to reduce inequalities and there was some evidence that this was achieved both in terms of reduction in calorie purchase [13], which is in line with what is known about socioeconomic differences in exposure to HFSS advertising [36], and subsequent modelled reductions in short-term obesity. However, the modelling results suggest that even a uniform reduction in calories across socioeconomic groups would produce significantly greater long-term benefits in the most deprived subgroups, with those who are most unhealthy at baseline likely to benefit most from small reductions in weight that will have less of an impact on healthier, wealthier individuals. Given that lower calorie purchase is likely to be associated with lower household expenditure, this suggests that advertising interventions are likely to be a highly progressive way of reducing obesity.

This is the first modelling study to investigate the potential long-term impacts of outdoor advertising restrictions (i.e. those not in the home); however several other studies have investigated the benefits of restrictions on television advertising in children and estimated that there would be significant reductions in childhood obesity resulting in long-term health benefits and reductions in healthcare costs [9–11]. This study therefore adds to the growing body of evidence that reducing exposure to HFSS advertising may help reduce obesity and overweight and improve long-term health. Reduction of childhood obesity was a particular aim of the TfL intervention; however, currently there is no information on how the intervention may have impacted children beyond the household purchasing data. The modelling presented here has assumed that the reductions in calorie purchase result in reductions in consumption that are evenly spread between all individuals in the household; this should include children of the household as well as adults. Whilst there is good evidence for the link between household purchase and household consumption of food [37], the distribution of intervention impacts within the household is likely to be more complex than modelled, and in particular will not capture impacts of older children or adults choosing HFSS foods for themselves outside the home, which will have likely underestimated total benefits. Furthermore, the model does not actually include individuals aged under 16 and therefore cannot estimate the impact on children and will likely underestimate the potential long-term benefits accrued through the household purchase reduction. Public health interventions acting on complex behaviours also have the potential to have unexpected spill-over effects that can lead to wider benefits, such as changes in social norms and attitudes leading to long-term healthier eating, or reformulation of products to fit within advertising guidelines. Equally, benefits may be mitigated in the longer term if for example industry behaviour shifts to focus investment in other marketing activities not covered by the advertising restrictions, or price decreases to offset reduced demand [38].

This analysis focussed on a policy that reduced advertising of HFSS foods only within the region of Greater London. Extending the policy to other regions of the UK and beyond should be feasible and relatively easy to achieve, although intervention effectiveness might be reduced in areas with lower population density and less well-connected transport networks. Whilst no intervention costs were relevant to this analysis, direct costs of changing or enforcing policy, or indirect costs due to loss of advertising revenue are likely to have been incurred elsewhere. Further research should examine the potential impact of the policy in other regions and other countries, which would enable local and national governments to make decisions about whether or not to implement it based on evidence relevant to their particular context. Using a societal perspective for future analyses would also enable business or local authority costs to be taken into account.

## Conclusions

In conclusion, restricting advertisement of HFSS products on the public transport network in London is likely to have led to considerable health and economic gains, whilst also reducing health inequalities. Out-of-home advertising policies represent an effective tool to help reduce population obesity and its long-term consequence.

## Supplementary Information

**Additional file 1: Supplementary Figure 1.** Graphical representation of sociodemographic cross tabulation between IMD and NS-SEC. A) Proportion of IMD quintiles that are in each NS-SEC group. B) Proportion of NS-SEC groups that are in each IMD quintile. IMD Index of multiple deprivation; SES; socioeconomic status; NS-SEC National Statistics Socioeconomic Classification. **Supplementary Figure 2.** PSA samples and mean costs and QALYs plotted on the cost-effectiveness plane for the basecase and sensitivity analysis scenarios for Greater London. The dotted line represents a cost-effectiveness threshold of £20,000 per QALY. SA1 no socioeconomic gradient in calorie reduction; SA2 no indirect metabolic effects; SA3 half calorie reduction; SA4 3 year duration of effect; SA5 1 year return to baseline BMI. **Supplementary Figure 3.** Expected reduction of cardiovascular disease cases (A-C) and new type 2 diabetes cases (D-F) over a 20 year time horizon, per 100,000 people of each IMD quintile, for sensitivity analyses: SA2 no indirect metabolic effects; SA3 half calorie reduction; SA4 3 year duration of effect; SA5 1 year return to baseline BMI. **Supplementary Table 1.** Additional obesity-related summary statistics by socioeconomic group for the modelled baseline population (adults aged 16 and over), based on sampling of 100,000 individuals from the Health Survey for England 2014, weighted to reflect the age, sex, ethnicity and socioeconomic distributions of Greater London. **Supplementary Table 2.** Breakdown of incremental modelled costs/cost savings by disease area for the basecase scenario compared with the no intervention control. Cost savings are shown as negative values. **Supplementary Table 3.** Incremental cost-effectiveness results by IMD quintile (per 100,000 individuals) for sensitivity analysis scenarios compared with no intervention. All outcomes are accumulated over a lifetime horizon. Cost savings are shown as negative values.

**Additional file 2. **Supplementary Technical Appendix.

## Data Availability

All data generated during this study is either included in the published article and supplementary information files, or available from the corresponding author on reasonable request.

## References

[1] Collaborators GO, Afshin A, Forouzanfar MH, Reitsma MB, Sur P, Estep K (2017). Health effects of overweight and obesity in 195 countries over 25 years. N Engl J Med.

[2] Dai H, Alsalhe TA, Chalghaf N, Ricco M, Bragazzi NL, Wu J (2020). The global burden of disease attributable to high body mass index in 195 countries and territories, 1990–2017: an analysis of the global burden of disease study. PLoS Med.

[3] Excess weight and COVID-19: insights from new evidence. Public Health England. 2020. https://www.gov.uk/government/publications/excess-weight-and-covid-19-insights-from-new-evidence. Accessed 20 May 2022.

[4] Luppino FS, de Wit LM, Bougy PF, Stijnen T, Cuijpers P, Penninx BWJH (2010). Overweight, obesity, and depression: A systematic review and meta-analysis of longitudinal studies. Arch Gen Psychiatry.

[5] Adult excess weight: patterns and trends. Public Health England. 2021. https://www.gov.uk/government/publications/adult-excess-weight-patterns-and-trends. Accessed 20 May 2022.

[6] National Child Measurement Programme, England 2020/21 School Year. NHS Digital. 2021. https://digital.nhs.uk/data-and-information/publications/statistical/national-child-measurement-programme/2020-21-school-year/deprivation. Accessed 20 May 2022.

[7] Emmert-Fees KMF, Karl FM, von Philipsborn P, Rehfuess EA, Laxy M (2021). Simulation modeling for the economic evaluation of population-based dietary policies: a systematic scoping review. Adv Nutr.

[8] Russell SJ, Croker H, Viner RM. The effect of screen advertising on children’s dietary intake: a systematic review and meta-analysis. Obes Rev. 2019;20(4):554–68. 10.1111/obr.12812 PMC6446725 30576057

[9] Brown V, Ananthapavan J, Veerman L, Sacks G, Lal A, Peeters A (2018). The potential cost-effectiveness and equity impacts of restricting television advertising of unhealthy food and beverages to australian children. Nutrients..

[10] Magnus A, Haby MM, Carter R, Swinburn B (2009). The cost-effectiveness of removing television advertising of high-fat and/or high-sugar food and beverages to Australian children. Int J Obes (Lond).

[11] Mytton OT, Boyland E, Adams J, Collins B, O’Connell M, Russell SJ, et al. The potential health impact of restricting less-healthy food and beverage advertising on UK television between 05.30 and 21.00 hours: a modelling study. PLoS Med. 2020;17(10):e1003212. 10.1371/journal.pmed.1003212 PMC7553286 33048922

[12] TfL Ad Policy: Approval Guidance Food and Non-Alcoholic Drink Advertising. Transport for London. 2019. http://content.tfl.gov.uk/policy-guidance-food-and-drink-advertising.pdf. Accessed 20 May 2022.

[13] Yau A, Berger N, Law C, Cornelsen L, Greener R, Adams J, et al. Changes in household food and drink purchases following restrictions on the advertisement of high fat, salt, and sugar products across the Transport for London network: a controlled interrupted time series analysis. PLOS Med. 2021;19(2):e1003915. 10.1371/journal.pmed.1003915. 10.1371/journal.pmed.1003915 PMC8853584 35176022

[14] Breeze P, Thomas C, Thokala P, Lafortune L, Brayne C, Brennan A (2020). The impact of including costs and outcomes of dementia in a health economic model to evaluate lifestyle interventions to prevent diabetes and cardiovascular disease. Med Decis Making.

[15] Breeze PR, Thomas C, Squires H, Brennan A, Greaves C, Diggle P (2017). Cost-effectiveness of population-based, community, workplace and individual policies for diabetes prevention in the UK. Diabet Med.

[16] Breeze PR, Thomas C, Squires H, Brennan A, Greaves C, Diggle PJ (2017). The impact of Type 2 diabetes prevention programmes based on risk-identification and lifestyle intervention intensity strategies: a cost-effectiveness analysis. Diabet Med.

[17] Thomas C, Sadler S, Breeze P, Squires H, Gillett M, Brennan A (2017). Assessing the potential return on investment of the proposed UK NHS diabetes prevention programme in different population subgroups: an economic evaluation. BMJ Open.

[18] Health Survey for England, 2014. NHS Digital. 2015. https://digital.nhs.uk/data-and-information/publications/statistical/health-survey-for-england/health-survey-for-england-2014. Accessed 20 May 2022.

[19] Estimates of the population for the UK, England and Wales, Scotland and Northern Ireland. Office for National Statistics. 2021. https://www.ons.gov.uk/peoplepopulationandcommunity/populationandmigration/populationestimates/datasets/populationestimatesforukenglandandwalesscotlandandnorthernireland. Accessed 20 May 2022.

[20] 2011 Census. Office for National Statistics. 2012. https://www.ons.gov.uk/census/2011census. Accessed 20 May 2022.

[21] English Indices of Deprivation 2015. Ministry of Housing, Communities & Local Government. 2015. https://www.gov.uk/government/statistics/english-indices-of-deprivation-2015. Accessed 20 May 2022.

[22] The Nutrient Profiling Model. Department of Health and Social Care. 2011. https://www.gov.uk/government/publications/the-nutrient-profiling-model. Accessed 20 May 2022.

[23] Lifestyle and Classification Data. National Readership Survey. 2021. http://www.nrs.co.uk/nrs-print/lifestyle-and-classification-data/. Accessed 20 May 2022.

[24] Gillick S, Quested T. Household food waste: restated data for 2007–2015. WRAP. 2018. https://wrap.org.uk/sites/default/files/2021-03/WRAP-Household-food-waste-restated-data-2007-2015_0.pdf. Accessed 20 May 2022.

[25] Body Weight Planner. National Institute of Diabetes and Digestive and Kidney Diseases. 2021. https://www.niddk.nih.gov/bwp. Accessed 20 May 2022.

[26] Hall KD, Sacks G, Chandramohan D, Chow CC, Wang YC, Gortmaker SL (2011). Quantification of the effect of energy imbalance on bodyweight. The Lancet.

[27] The National Statistics Socio-economic classification (NS-SEC). Office for National Statistics. 2021. https://www.ons.gov.uk/methodology/classificationsandstandards/otherclassifications/thenationalstatisticssocioeconomicclassificationnssecrebasedonsoc2010. Accessed 20 May 2022.

[28] Breeze P, Squires H, Chilcott J, Stride C, Diggle PJ, Brunner E (2016). A statistical model to describe longitudinal and correlated metabolic risk factors: the Whitehall II prospective study. J Public Health (Oxf).

[29] Anderson JW, Konz EC, Frederich RC, Wood CL (2001). Long-term weight-loss maintenance: a meta-analysis of US studies. Am J Clin Nutr.

[30] Guide to the methods of technology appraisal. National Institute of Health and Care Excellence (NICE). 2013. https://www.nice.org.uk/process/pmg9/resources/guide-to-the-methods-of-technology-appraisal-2013-pdf-2007975843781. Accessed 20 May 2022. 27905712

[31] Public Health Outcomes Framework Health Inequalities Dashboard. Public Health England. 2021. https://analytics.phe.gov.uk/apps/health-inequalities-dashboard/. Accessed 20 May 2022.

[32] Health Equity Assessment Toolkit: Technical Notes. World Health Organisation. 2017. https://cdn.who.int/media/docs/default-source/gho-documents/health-equity/health-equity-assessment-toolkit/heat_4.0_technical_notes.pdf?sfvrsn=24a0e227. Accessed 20 May 2022.

[33] Hippisley-Cox J, Coupland C (2017). Development and validation of QDiabetes-2018 risk prediction algorithm to estimate future risk of type 2 diabetes: cohort study. BMJ.

[34] Cobiac LJ, Scarborough P (2021). Modelling future trajectories of obesity and body mass index in England. PLoS ONE.

[35] Janssen F, Bardoutsos A, Vidra N (2020). Obesity prevalence in the long-term future in 18 European countries and in the USA. Obes Facts.

[36] Yau A, Adams J, Boyland EJ, Burgoine T, Cornelsen L, de Vocht F (2021). Sociodemographic differences in self-reported exposure to high fat, salt and sugar food and drink advertising: a cross-sectional analysis of 2019 UK panel data. BMJ Open.

[37] Appelhans BM, French SA, Tangney CC, Powell LM, Wang Y. To what extent do food purchases reflect shoppers’ diet quality and nutrient intake? Int J Behav Nutr Phys Act. 2017;14(1):46. 10.1186/s12966-017-0502-2 PMC5387266 28399887

[38] Dubois P, Griffith R, O’Connell M (2018). The effects of banning advertising in junk food markets. Rev Econ Stud.

