# Supplementary material for: The health, cost and equity impacts of restrictions on the advertisement of high fat, salt and sugar products across the transport for London network: a health economic modelling study
Source: Int J Behav Nutr Phys Act. 2022 Jul 27;19:93. doi: 10.1186/s12966-022-01331-y (PMC9326956; doi:10.1186/s12966-022-01331-y)
Supplement: Supplementary file 1 — Additional file 1: Supplementary Figure 1. Graphical representation of sociodemographic cross tabulation between IMD and NS-SEC. A) Proportion of IMD quintiles that are in each NS-SEC group. B) Proportion of NS-SEC groups that are in each IMD quintile. IMD Index of multiple deprivation; SES; socioeconomic status; NS-SEC National Statistics Socioeconomic Classification. Supplementary Figure 2. PSA samples and mean costs and QALYs plotted on the cost-effectiveness plane for the basecase and sensitivity analysis scenarios for Greater London. The dotted line represents a cost-effectiveness threshold of £20,000 per QALY. SA1 no socioeconomic gradient in calorie reduction; SA2 no indirect metabolic effects; SA3 half calorie reduction; SA4 3 year duration of effect; SA5 1 year return to baseline BMI. Supplementary Figure 3. Expected reduction of cardiovascular disease cases (A-C) and new type 2 diabetes cases (D-F) over a 20 year time horizon, per 100,000 people of each IMD quintile, for sensitivity analyses: SA2 no indirect metabolic effects; SA3 half calorie reduction; SA4 3 year duration of effect; SA5 1 year return to baseline BMI. Supplementary Table 1. Additional obesity-related summary statistics by socioeconomic group for the modelled baseline population (adults aged 16 and over), based on sampling of 100,000 individuals from the Health Survey for England 2014, weighted to reflect the age, sex, ethnicity and socioeconomic distributions of Greater London. Supplementary Table 2. Breakdown of incremental modelled costs/cost savings by disease area for the basecase scenario compared with the no intervention control. Cost savings are shown as negative values. Supplementary Table 3. Incremental cost-effectiveness results by IMD quintile (per 100,000 individuals) for sensitivity analysis scenarios compared with no intervention. All outcomes are accumulated over a lifetime horizon. Cost savings are shown as negative values. [file 12966_2022_1331_MOESM1_ESM.docx]

Supplementary Figure 1. Graphical representation of sociodemographic cross tabulation between IMD and NS-SEC. A) Proportion of IMD quintiles that are in each NS-SEC group. B) Proportion of NS-SEC groups that are in each IMD quintile. IMD Index of multiple deprivation; SES; socioeconomic status; NS-SEC National Statistics Socioeconomic Classification.

Supplementary Figure 2. PSA samples and mean costs and QALYs plotted on the cost-effectiveness plane for the basecase and sensitivity analysis scenarios for Greater London. The dotted line represents a cost-effectiveness threshold of £20,000 per QALY. SA1 no socioeconomic gradient in calorie reduction; SA2 no indirect metabolic effects; SA3 half calorie reduction; SA4 3 year duration of effect; SA5 1 year return to baseline BMI.

Supplementary Figure 3. Expected reduction of cardiovascular disease cases (A-C) and new type 2 diabetes cases (D-F) over a 20 year time horizon, per 100,000 people of each IMD quintile, for sensitivity analyses: SA2 no indirect metabolic effects; SA3 half calorie reduction; SA4 3 year duration of effect; SA5 1 year return to baseline BMI.

Supplementary Table 1. Additional obesity-related summary statistics by socioeconomic group for the modelled baseline population (adults aged 16 and over), based on sampling of 100,000 individuals from the Health Survey for England 2014, weighted to reflect the age, sex, ethnicity and socioeconomic distributions of Greater London.

Supplementary Table 2. Breakdown of incremental modelled costs/cost savings by disease area for the basecase scenario compared with the no intervention control. Cost savings are shown as negative values.

Supplementary Table 3. Incremental cost-effectiveness results by IMD quintile (per 100,000 individuals) for sensitivity analysis scenarios compared with no intervention. All outcomes are accumulated over a lifetime horizon. Cost savings are shown as negative values.
