# Supplementary material for: The health, cost and equity impacts of restrictions on the advertisement of high fat, salt and sugar products across the transport for London network: a health economic modelling study
Source: Int J Behav Nutr Phys Act. 2022 Jul 27;19:93. doi: 10.1186/s12966-022-01331-y (PMC9326956; doi:10.1186/s12966-022-01331-y)
Supplement: Supplementary file 2 — Additional file 2. Supplementary Technical Appendix. [file 12966_2022_1331_MOESM2_ESM.docx]

File S1: Supplementary Technical Appendix

The health, cost and equity impacts of restrictions on the advertisement of high fat, salt and sugar products across the Transport for London network: A modelling study

Authors: Thomas, C. Breeze, P. Cummins, S. Cornelsen, L. Yau, A. Brennan, A.

# Contents

[1. Contents 2](#_Toc90565771)

[2. Developing the Conceptual Model 2](#_Toc90565772)

[3. Model Structure 2](#_Toc90565773)

[4. Baseline Population Characteristics: Health Survey for England 2](#_Toc90565774)

[Exclusion Criteria 2](#_Toc90565775)

[Re-weighting HSE 2014 data to Greater London characteristics 2](#_Toc90565776)

[HSE 2014 Missing data imputation 2](#_Toc90565777)

[5. GP Attendance in the General Population 2](#_Toc90565778)

[6. Longitudinal Trajectories of Metabolic Risk Factors 2](#_Toc90565779)

[Whitehall II Data Analysis 2](#_Toc90565780)

[ELSA Data Analysis 2](#_Toc90565781)

[BMI Trajectory 2](#_Toc90565782)

[Glycaemic Trajectory in Non-Diabetics/undiagnosed Diabetes 2](#_Toc90565783)

[HbA1c trajectory in type 2 diagnosed diabetics 2](#_Toc90565784)

[Total Cholesterol and HDL Cholesterol Trajectories in Individuals not receiving Statins 2](#_Toc90565785)

[Total Cholesterol and HDL Cholesterol Trajectories in Individuals receiving Statins 2](#_Toc90565786)

[SBP Trajectories in Individuals Not receiving Anti-hypertensive treatment 2](#_Toc90565787)

[SBP Trajectories in Individuals receiving anti-hypertensive treatment 2](#_Toc90565788)

[Metabolic Risk factor screening 2](#_Toc90565789)

[Diagnosis and Treatment Initiation 2](#_Toc90565790)

[Diabetes Diagnosis 2](#_Toc90565791)

[7. Comorbid Outcomes and Mortality 2](#_Toc90565792)

[Cardiovascular Disease 2](#_Toc90565793)

[First Cardiovascular event 2](#_Toc90565794)

[First Cardiovascular event calibration 2](#_Toc90565795)

[First Cardiovascular event inputs 2](#_Toc90565796)

[Subsequent Cardiovascular events 2](#_Toc90565797)

[Congestive Heart Failure 2](#_Toc90565798)

[Microvascular Complications 2](#_Toc90565799)

[Cancer 2](#_Toc90565800)

[Breast cancer 2](#_Toc90565801)

[Colorectal cancer 2](#_Toc90565802)

[Osteoarthritis 2](#_Toc90565803)

[Depression 2](#_Toc90565804)

[Dementia 2](#_Toc90565805)

[Dementia Diagnosis 2](#_Toc90565806)

[Disease Progression 2](#_Toc90565807)

[Mortality 2](#_Toc90565808)

[Cardiovascular Mortality 2](#_Toc90565809)

[Cancer Mortality 2](#_Toc90565810)

[Other cause Mortality (including diabetes and Dementia risk) 2](#_Toc90565811)

[8. Direct Health Care Costs 2](#_Toc90565812)

[GP attendance 2](#_Toc90565813)

[Diabetes 2](#_Toc90565814)

[Metformin Monotherapy 2](#_Toc90565815)

[Metformin plus Gliptins 2](#_Toc90565816)

[Insulin plus Oral Anti-diabetics 2](#_Toc90565817)

[Statins 2](#_Toc90565818)

[Anti-hypertensives 2](#_Toc90565819)

[Cardiovascular Events 2](#_Toc90565820)

[Renal Failure 2](#_Toc90565821)

[Foot Ulcers 2](#_Toc90565822)

[Amputation 2](#_Toc90565823)

[Blindness 2](#_Toc90565824)

[Cancer 2](#_Toc90565825)

[Osteoarthritis 2](#_Toc90565826)

[Depression 2](#_Toc90565827)

[Dementia 2](#_Toc90565828)

[Cost of Diagnosis 2](#_Toc90565829)

[Ongoing healthcare costs 2](#_Toc90565830)

[9. Social Care costs 2](#_Toc90565831)

[Stroke 2](#_Toc90565832)

[Dementia 2](#_Toc90565833)

[10. Utilities 2](#_Toc90565834)

[Baseline Utility 2](#_Toc90565835)

[Utility Decrements 2](#_Toc90565836)

[11. Intervention Effectiveness 2](#_Toc90565837)

[Intervention Effectiveness 2](#_Toc90565838)

[Duration of Intervention Effect 2](#_Toc90565839)

[12. Probabilistic Sensitivity Analysis 2](#_Toc90565840)

[13. Model Validation 2](#_Toc90565841)

[14. References 2](#_Toc90565842)

# Developing the Conceptual Model

The conceptual model was developed according to a new conceptual modeling framework for complex public health models (1). In line with this framework the conceptual model was developed in collaboration with a project stakeholder group comprising health economists, public health specialists, research collaborators from other SPHR groups, diabetologists, local commissioners and lay members. The initial broad scope for the conceptual model was based on the structure of previous diabetes prevention models used for National Institute for Health and Care Excellence public health guidance (2, 3) and discussions with experts in diabetes prevention modeling. The model was further extended to include Dementia as a possible health outcome for individuals aged over 60 years in the model.

# Model Structure

We developed an individual patient simulation that estimates individuals’ health in yearly cycles until death. The simulation draws baseline demographic and clinical status for individuals sampled from the Health Survey for England (HSE) 2014 (4). The simulation estimates yearly changes in metabolic risk factors based upon the individuals’ baseline characteristics. Within each annual cycle the individuals may be screened for hypertension, dyslipidaemia or diabetes during a visit to the General Practitioner (GP). Opportunistic screening is used to determine diabetes diagnosis or the initiation of anti-hypertensive treatment or statins. Baseline characteristics and metabolic risk factors determine the individuals’ probability of cardiovascular events, diabetes microvascular complications, cancer, osteoarthritis and depression. Individuals within the model may die in any cycle as a result of cardiovascular disease, cancer or from other causes.

Figure 1 illustrates the sequence of updating clinical characteristics and clinical events that are estimated within a cycle of the model. This sequence is repeated for every annual cycle of the model.

The first stage of the sequence updates the age of the individual. The second stage estimates how many times the individual attends the GP. The third stage estimates the change in BMI of the individual from the previous period. In the fourth stage, if the individual has not been diagnosed as diabetic (Diabetes_Dx=0) their change in glycaemia is estimated using the Whitehall II model. If they are diabetic (Diabetes_Dx=1), it is estimated using the UKPDS model. In stages five and six the individual’s blood pressure and cholesterol are updated using the Whitehall II model if the individual is not identified as hypertensive or receiving statins. In stage seven, the individual may undergo assessment for diabetes, hypertension and dyslipidaemia during a GP consultation. From stage eight onwards the individual may experience cardiovascular outcomes, diabetes related complications, cancer, osteoarthritis, depression, dementia, and updated cognitive decline associated with dementia diagnosis. If the individual has a history of cardiovascular disease (CVD history=1), they follow a different pathway in stage eight to those without a history of cardiovascular disease (CVD history=0). Individuals with HbA1c greater than 48 mmol/mol (6.5%) are assumed to be at risk of diabetes related complications. Individuals who do not have a history of cancer (Cancer history=0) are at risk of cancer diagnosis, whereas those with a diagnosis of cancer (Cancer history=1) are at risk of mortality due to cancer. Individuals without a history of osteoarthritis or depression may develop these conditions in stages 12 and 13. In stage 14 individuals with dementia have their cognitive status updated and those individuals aged over 60 without a diagnosis of dementia may receive a diagnosis. Finally, all individuals are at risk of dying due non cardiovascular or cancer mortality. Death from renal disease is included in the estimate of other-cause mortality.

The modeling structure and cycle sequence is explained in more detail using a hypothetical patient below:

Consider a white male aged 53 sampled from the baseline population, referred to hereafter as Mr X. Mr X has a series of baseline demographics informed by the baseline population dataset, or imputation if missing. These characteristics influence his future health outcomes in the model. In the first cycle of the model the age of Mr X is 53. In this cycle Mr X’s attendance at the GP is generated and recorded within the model dependent upon his age and gender. In the first cycle of the model Mr X’s BMI is extracted from his baseline data. The effect of an intervention on BMI in the first 12 months is applied here if required. Similarly baseline values for HbA1c, systolic blood pressure (SBP), total cholesterol, and HDL cholesterol are extracted from his baseline dataset and modified for treatment effect if necessary. If Mr X has attended the GP in this cycle he may receive opportunistic screening for diabetes, hypertension or high cardiovascular risk if he meets certain risk criteria, agreed by the stakeholder group. If he is diagnosed with any of these conditions/risks, treatments are initiated according to current guidelines in the UK for diabetes diagnosis, anti-hypertensive treatment and statin treatment. If Mr X receives any of these treatments his HbA1c, SBP and/or total cholesterol are reduced accordingly.

Having established Mr X’s metabolic risk profile the model determines if Mr X experiences any major health events in this first cycle. If Mr X does not have a history of cardiovascular disease (CVD) the model estimates the probability that he has a fatal or non-fatal cardiovascular event in this cycle. The event is determined using a Bernoulli trial. If Mr X has a history of CVD his probability of a progressing to unstable angina, MI, stroke or a fatal event is determined. If Mr X has HbA1c greater than 48 mmol/mol (6.5%) or a diagnosis of diabetes, the probability of foot ulcer, renal disease, amputation and blindness are calculated and evaluated using a Bernoulli trial. If Mr X does not have diabetes he is not at risk of these complications in this cycle.

In the next stage of the cycle Mr X may develop breast or colon cancer if he has not already got a history of cancer. The probability of these complications is generated and evaluated in a Bernoulli trial. If Mr X has a history of cancer, he is at risk of mortality due to cancer in this cycle. If Mr X does not have osteoarthritis the probability of developing this complication is evaluated in this cycle and a diagnosis is given according to a Bernoulli trial. If Mr X has a diagnosis of osteoarthritis his health status for this complication remains unchanged. Similarly, if Mr X does not have depression the probability of developing it is evaluated in this cycle and a diagnosis is given according to a Bernoulli trial. If Mr X has a diagnosis of depression his health status for this complication remains unchanged. If Mr X is over 60 years old and does not have a diagnosis of dementia, he may receive a new diagnosis according to a Bernoulli trial using his current probability of a dementia diagnosis. If Mr X has an existing diagnosis of dementia his MMSE score will be updated to reflect any deterioration in memory and disease severity.

Finally, assuming Mr X has not experienced a fatal event due to CVD or cancer, the probability of death is calculated and evaluated in a Bernoulli trial based on Office of National Statistics life tables combined with hazard ratios for dementia and diagnosis (5). If Mr X remains alive he proceeds to the next cycle. If Mr X dies his health status, costs and QALYs are stored and he is removed from the model.

In the second and subsequent cycles, the model proceeds through a similar sequence of events. However, Mr X firstly ages by the cycle length of one year. A new number of GP visits within the cycle is generated. BMI will increase or decrease according to a trajectory assigned to Mr X at baseline, and intervention effect maintenance if relevant. Similarly, HbA1c, SBP, total cholesterol, and HDL cholesterol all change in this period on a prespecified trajectory and intervention effect. Mr X may undergo opportunistic screening as specified in year one. The sequence of evaluations to determine health events and complications experienced by Mr X in this cycle is the same as described above, however Mr X’s metabolic risk factors, treatments, and history are updated with the changes described above.

Figure 1: Model Schematic

2. GP visits

.

3.

BMI

.

4.a. Glucose

.

Diabetes_Dx

=1

Diabetes_Dx

=0

1

. Age

4.a. HbA1c+treatment

.

5.a. Blood pressure

.

5.b. Blood pressure

.

Hypertenson

=0

Hypertenson

=1

6.a. Cholesterol

6.a. Cholesterol

Statin=0

Statin=1

7. Screening

.

8.a. CVD events

8.b. CVD events

CVD history=0

CVD history=1

9. CVD

Mortality

10. Renal failure, ulcer,

amputation and blind

HbA

<6.5

HBA>6.5

11.a. Cancer events

11.b. Cancer events

Cancer history=0

Cancer history=1

11.b. Cancer

Mortality

Osteo

history=1

12.

Osteo

events

Osteo

history=0

13. Depression

Depression=0

Depression=1

15. All cause

mortality

Dementia=0

Dementia=1

14.b Update MMSE

14.a Dementia

# Baseline Population Characteristics: Health Survey for England

The model required demographic, anthropometric and metabolic characteristics that would be representative of the UK general population. The Heath Survey for England (HSE) was suggested by the stakeholder group because it collects up-to-date cross-sectional data on the characteristics of all ages of the English population. It also benefits from being a reasonably good representation of the socioeconomic profile of England. A major advantage of this dataset is that includes important clinical risk factors such as HbA1c, SBP, and cholesterol. The characteristics of individuals included in the cost-effectiveness model were based sampled from the HSE 2014 dataset (4). The whole dataset was obtained from the UK Data Service.

## Exclusion Criteria

The total sample size of the HSE 2014 was 10,080. Individuals who were younger than 16 years (N=2003) were excluded from the sample. This left a final sample size of 8,077 individuals. Summary statistics for the data extracted from the HSE2014 dataset are reported in Table 1.

Table 1: Characteristics of final sample from HSE 2014 (N=8,077)

| Variable name (description) | Mean | Median | SD | Missing (N) |
| --- | --- | --- | --- | --- |
| Age | 50.02 | 49.00 | 18.63 | 0 |
| Income | 33810 | 24700 | 29246 | 1567 |
| Weight | 77.61 | 75.80 | 17.26 | 990 |
| Height | 167.7 | 167.3 | 9.74 | 938 |
| BMI | 27.52 | 26.66 | 5.48 | 1132 |
| Waist | 93.10 | 92.55 | 14.39 | 2818 |
| Hip | 105.6 | 104.1 | 15.95 | 2813 |
| Waist-Hip ratio | 0.881 | 0.879 | 0.092 | 2832 |
| Total Cholesterol | 5.194 | 5.1 | 1.104 | 4176 |
| HDL Cholesterol | 1.545 | 1.5 | 0.452 | 4175 |
| HbA1c | 5.615 | 5.4 | 0.785 | 4183 |
| SBP | 126.2 | 124.5 | 17.22 | 3208 |
| DBP | 72.75 | 72.5 | 11.07 | 3408 |
| Total units alcohol | 12.6 | 6.04 | 21.55 | 177 |
| Minute vigorous exercise | 65.45 | 30.0 | 107.66 | 1170 |
| Minutes walking | 89.45 | 60.00 | 114.86 | 1238 |
| EQ-5D | 0.8767 | 1 | 0.189 | 187 |

Table 2: Summary data for categorical (N=8,077)

| Variable name (description) | Category | N | % |
| --- | --- | --- | --- |
| Sex | Male | 3588 | 44.4% |
|  | Female | 4489 | 55.6% |
|  | Missing | 0 | 0% |
| Origin | White British | 6653 | 82.4% |
|  | White Irish | 76 | 0.9% |
|  | White other | 421 | 5% |
|  | White and Black Caribbean | 32 | 0.3% |
|  | White and Black African | 11 | 0.1% |
|  | White and Asian | 17 | 0.2% |
|  | Other mixed | 44 | 0.5% |
|  | Indian | 198 | 2% |
|  | Pakistani | 146 | 2% |
|  | Bangladeshi | 47 | 0.6% |
|  | Chinese | 44 | 0.5% |
|  | Other Asian | 91 | 1% |
|  | African | 105 | 1.3% |
|  | Caribbean | 73 | 0.9% |
|  | Other Black | 18 | 0.2% |
|  | Arab | 25 | 0.3% |
|  | Other | 44 | 0.5% |
|  | Missing | 32 | 0.4% |
| QIMD | 0.53-8.49 (least deprived) | 1777 | 22% |
|  | 8.49-13.79 | 1611 | 20.0% |
|  | 13.79-21.35 | 1557 | 19.3% |
|  | 21.35-34.17 | 1602 | 19.8% |
|  | 34.17-87.80 (most deprived) | 1530 | 18.9% |
| Smoking group | Current | 1444 | 17.9% |
|  | Ex-smoker | 2033 | 25.2% |
|  | Never smoke | 4535 | 56.1% |
|  | Missing | 65 | 0.8% |
| Smoking level | Low smoker | 534 | 6.6% |
|  | Moderate smoker | 627 | 7.8% |
|  | Heavy smoker | 276 | 3.4% |
|  | Don’t know | 7 | 0.1% |
|  | Non-smoker | 6570 | 81.3% |
|  | Missing | 63 | 0.8% |
| Hypertensive treatment | Yes | 1492 | 18.5% |
|  | No | 478 | 5.9% |
|  | Missing | 6107 | 75.6% |
| Statins | No | 4545 | 56.3% |
|  | Yes | 946 | 11.7% |
|  | Missing | 2586 | 32.0% |
| Long term illness 1 | 15:Stroke | 32 | 0.4% |
|  | 16:Heart attack/angina | 67 | 0.8% |
|  | 17:Other heart | 148 | 2% |
|  | 34:Arthritis/rheumatism | 396 | 4.9% |
|  | Missing | 4683 | 58.0% |
| Long term illness 2 | 15:Stroke | 14 | 0.2% |
|  | 16:Heart attack/angina | 56 | 0.7% |
|  | 17:Other heart | 88 | 1.1% |
|  | 34:Arthritis/rheumatism | 166 | 2% |
|  | Missing | 6275 | 77.7% |
| Long term illness 3 | 15:Stroke | 13 | 0.2% |
|  | 16:Heart attack/angina | 8 | 0.1% |
|  | 17:Other heart | 30 | 0.4% |
|  | 34:Arthritis/rheumatism | 89 | 1.1% |
|  | Missing | 7176 | 88.8% |
| Long term illness 4 | 15:Stroke | 6 | 0.1% |
|  | 16:Heart attack/angina | 8 | 0.1% |
|  | 17:Other heart | 13 | 0.2% |
|  | 34:Arthritis/rheumatism | 42 | 0.5% |
|  | Missing | 7623 | 94.4% |
| Long term illness 5 | 15:Stroke | 8 | 0.1% |
|  | 16:Heart attack/angina | 3 | 0.04% |
|  | 17:Other heart | 13 | 0.2% |
|  | 34:Arthritis/rheumatism | 15 | 0.2% |
|  | Missing | 7860 | 97.3% |
| Long term illness 6 | 15:Stroke | 1 | 0.01% |
|  | 16:Heart attack/angina | 2 | 0.02% |
|  | 17:Other heart | 2 | 0.02% |
|  | 34:Arthritis/rheumatism | 13 | 0.2% |
|  | Missing | 7975 | 98.7% |
| Diabetes (including undiagnosed) | Yes | 588 | 6.7% |
|  | No | 3595 | 44.9% |
|  | Missing | 3866 | 48.3% |
| Depression | Yes self reported diagnosis | 1107 | 13.7% |
|  | Yes not self reported diagnosis | 401 | 5.0% |
|  | No | 3977 | 49.2% |
|  | Missing | 2592 | 32.1% |
| Dementia | Yes self reported diagnosis | 11 | 0.1% |
|  | Yes not self reported diagnosis | 7 | 0.1% |
|  | No | 5467 | 67.7% |
|  | Missing | 2592 | 32.1% |
| Alcohol Problem | Yes | 67 | 0.8% |
|  | No | 61 | 0.8 % |
|  | Missing | 7949 | 98% |

A complete dataset was required for all individuals at baseline. However, no measurements for Fasting Plasma Glucose (FPG) or 2 hour glucose were obtained for the HSE 2014 cohort. In addition, the questionnaire did not collect information about individual family history of diabetes or family history of CVD. These variables were imputed from the Whitehall II dataset (see below) (6).

Many individuals were lacking responses to some questions but had data for others. One way of dealing with this was to exclude all individuals with incomplete data from the sample. However, this would have reduced the sample size and representativeness dramatically, which would have been detrimental to the analysis. It was decided that it would be better to make use of all the data available to represent a broad range of individuals within the UK population. With this in mind, we decided to use assumptions and imputation models to estimate missing data.

## Re-weighting HSE 2014 data to Greater London characteristics

The HSE 2014 contains survey weights, which enable the sample to represent the population of England by either increasing (weight > 1) or reducing (weight < 1) the importance of each individual (4). This works well for a national model, but needs adaptation to make a locally useful model, so a set of local weights were developed.

The local weights were generated through a calibration weighting approach using iterative proportional fitting (7), using local data about age, sex, ethnic group and deprivation quintile. To adjust these survey weights to local level, two datasets were used (Table 3).

Table 3: Local Population Demographic Data Used.

| Population Characteristic | Data source |
| --- | --- |
| Age/Sex profile | 2011 Census (8) |
| Ethnic group | 2011 Census (8) |
| Deprivation quintile | 2015 English Indices of Deprivation (9) |

The greatest possible number of population demographic characteristic breakdowns was used to give the best possible fit to local authority populations.The 2011 census provided population data at Lower super output area (LSOA) level with breakdowns for age and ethnicity (table LC2109EWLS), and at local authority level by age groups, sex and ethnicity (table DC2101EW) (8). Data was processed in order to obtain the size of each local population for each age group (16 groups: 16-17, 18-19, 20-24, 25-29, 30-34, 35-39, 40-44, 45-49, 50-54, 55-59, 60-64, 65-69, 70-74, 75-79, 80-84 and 85+), ethnic group (3 groups: White, Asian and other) and sex (males and females), cross referencing between the characteristics.

The Index of Multiple Deprivation 2015 (IMD 2015) was obtained from the English Indices of Deprivation 2015271, which contains the ranks and quintiles for the IMD at Lower-layer Super Output Area (LSOA) level (9). This was first processed to align deprivation quintiles with the HSE 2014 (1 = least deprived and 5 = most deprived) (4). Modelling simply age/sex/ethnicity versus IMD assumes no relationship between ethnicity and deprivation. This was thought to be a weak assumption and to overcome it, data by age and ethnicity were obtained at LSOA level and combined with LSOA-level IMD to generate individual IMD target cross-tabs for each ethnic group. This allowed a local authority-level variation in patterns of deprivation across ethnic groups.

Given that the estimated population of each locality has grown since 2011/2015, all data was normalised to reflect the current total population estimates (for Greater London this was 7,149,281 individuals based on UK mid-year estimates for 2020) (10). To do this, it was assumed that the population had grown in the same proportion across all demographics.

Inputs to the IPF algorithm were column (age/sex/ethnicity) and row (IMD/ethnicity) totals for local data (as described above) and HSE 2014 data (4). There was a maximum of 96 possible columns (age, sex, ethnic characteristics: 16 x 2 x 3). However, where a given column contained no individuals in the HSE 2014 (one column only: females, ethnicity ‘Asian’, aged 80-85) this was combined with neighbouring columns (ages 85+) which contained at least one respondent, giving a total of 95 columns. There was a maximum of 15 possible rows (ethnicity/IMD: 3 x 5). There was at least one individual in each of these rows in the HSE 2014, meaning no rows had to be combined and a total of 15 were included. Equally, in some local areas, there were no individuals in a given cell (this was true particularly for the y variables as there were some local areas where only two or three quintiles of deprivation were represented). Where this was the case, these cells were set to a very small number rather than zero, as zeros cause problems with IPF.

Two dimensional IPF was used to estimate cross-tab data between the population demographics and the IMD for each local area. This produced a matrix for each local area showing the numbers of people with each of the demographic characteristics. HSE 2014 data was used to produce a similar matrix. Survey weights for each local area were then calculated as follows:

Wij = (1/nijs) x nijp

Where wij is the weight applying to an individual in group ij (row i, column j), nijs is the number of people group ij in the sample (HSE 2014) and nijp is the number of people in group ij in the population (given local authority).

Survey weights for all HSE 2014 individuals in a given local authority add up to the total population of that local authority, and can be used as a multiplier for per-person model outputs to develop local authority-level outputs. Weights from the 33 local authority areas that make up the Greater London area ref were summed for each individual to obtain Greater London weights. The re-weighted Health Survey for England was sampled with replacement to get a final simulation population of 100,000.

## HSE 2014 Missing data imputation

Missing data were imputed using the techniques described below.

#### Ethnicity

Only a small number of individuals had missing data for ethnicity. In the QRISK2 algorithm the indicator for white included individuals for whom ethnicity is not recorded. In order to be consistent with the QRISK2 algorithm we assumed that individuals with missing ethnicity data were white.

#### Anthropometric data

Data were imputed using multiple imputation methods from the mice package in R. Predictive mean methods imputation methods were used to impute missing data based on age, socioeconomic status, anti-hypertensive treatment, diabetes diagnosis, anthropometric, metabolic data and EQ-5D within the dataset. Limiting the number of relationships to include in the imputation method was necessary to reduce the computational time required by the imputation.

#### Metabolic data

Data were imputed using multiple imputation methods from the mice package in R. Predictive mean methods imputation methods were used to impute missing data based on age, socioeconomic status, anti-hypertensive treatment, diabetes diagnosis, anthropometric, metabolic data and EQ-5D within the dataset. Limiting the number of relationships to include in the imputation method was necessary to reduce the computational time required by the imputation.

#### Treatment for Hypertension and Statins

A large proportion of individuals had missing data for questions relating to whether they received treatment for hypertension or high cholesterol. The majority of non-responses to these questions were coded to suggest that the question was not applicable to the individual. As a consequence it was assumed that individuals with missing treatment data were not taking these medications.

#### Anxiety/Depression

Most individuals who had missing data for anxiety and depression did so because the question was not applicable. A small sample N=69 refused to answer the question. We assumed that individuals with missing data for anxiety and depression did not have severe anxiety/depression.

#### Smoking

Individuals with missing data for smoking status were assumed to be non-smokers, without a history of smoking.

#### Rheumatoid Arthritis

Indiivduals reporting existing arthritis/rheumatism were assigned to a history rheumatoid arthritis.

Atrial Fibrillation

Individuals reporting “other heart conditions” in response to questions about long-standing illnesses were assumed to have a history of Atrial Fibrillation.

#### Family history of diabetes

No questions in the HSE referred to the individual having a family history of diabetes, so this data had to be imputed. It was important that data was correlated with other risk factors for diabetes, such as HbA1c and ethnicity. We analysed a cross-section of the Whitehall II dataset to generate a logistic regression to describe the probability that an individual has a history of diabetes conditional on their HbA1c and ethnic origin. The model is described in Table 4.

Table 4: Imputation model for history of diabetes

|  | Coefficient | Standard error |
| --- | --- | --- |
| Intercept | -3.29077 | 0.4430 |
| HbA1c | 0.28960 | 0.0840 |
| HDL Cholesterol | 0.81940 | 0.1388 |

#### History of Cardiovascular disease

Individuals with a history of cardiovascular disease were assigned to a health status of either stable angina, unstable angina, myocardial infarction, or stroke based on responses to health survey for England responses to long standing conditions. Individuals reporting stroke were assigned to stroke, heart attak/angina to unstable angina and MI at random using distributions estimated in the statins HTA (11).

#### Baseline EQ-5D

Data were imputed using multiple imputation methods from the mice package in R. Predictive mean methods imputation methods were used to impute missing data based on age, socioeconomic status, anti-hypertensive treatment, diabetes diagnosis, anthropometric, metabolic data and EQ-5D within the dataset. Limiting the number of relationships to include in the imputation method was necessary to reduce the computational time required by the imputation.

# GP Attendance in the General Population

GP visit frequency was simulated in the dataset for two reasons; firstly to estimate healthcare utilisation for the general population; secondly to predict the likelihood that individuals participate in opportunistic screening for diabetes and vascular risks. It was useful to develop a model of GP attendance to be conditional on characteristics in the cost-effectiveness model that are known to be associated with GP attendance, such as age and comorbidities. A negative binomial model was used to generate count data and a skewed distribution as observed in the dataset.

|  | $\mu_{i}=exp(x_{i}\beta)$ |  |
| --- | --- | --- |

The dispersion parameter of the Negative Binomial distribution $v_{i}$ was sampled from a gamma distribution with mean 1 and variance $\alpha$ based on estimates reported in Table 6. The dose was estimated from the Poisson function.

|  | $p\left( Y=y \vert y>0,x \right)=\frac{\left( v_{i}\mu_{i} \right)^{y}e^{-\left( v_{i}\mu_{i} \right)}}{y!}$ |  |
| --- | --- | --- |

The HSE 2014 did not collect data on GP attendance frequency, therefore an alternative UK survey was sought. The South Yorkshire cohort collected data about the frequency of GP attendance in the past 3 months from a representative cross-section of individuals in South Yorkshire (12). All individuals in the cohort were included in the analysis, including those with diabetes. The characteristics of the study population are reported in Table 5.

Table 5: Characteristics of the first wave of the South Yorkshire Cohort (N=27,806)

|  | Number | Percentage |  |
| --- | --- | --- | --- |
| Male | 12,155 | 43.7 |  |
| White | 26,419 | 95.0 |  |
| Non-smoker | 23,158 | 83.3 |  |
| Employed (inc. self-employed) | 18,502 | 66.5 |  |
| Long-standing illness (any) | 16,664 | 60.0 |  |
| Diabetes | 2,000 | 7.2 |  |
| Cardiovascular disease | 2,438 | 8.8 |  |
| Hypertension | 5,653 | 20.3 |  |
|  | Mean | Standard deviation | Median |
| Age | 54.45 | 17.25 | 57.00 |
| BMI | 26.46 | 5.05 | 25.68 |
| EQ-5D (TTO) | 0.803 | 0.253 | 0.848 |
| GP attendances in past 3 months | 2.03 | 1.83 | 1.00 |
| BMI Body Mass Index; EQ-5D 5 dimensions Euroqol (health related quality of life index) | | | |

The coefficients of the Negative Binomial model described in Table 6 were used to estimate the first parameter of the Negative Binomial distribution$\mu_{i}$. Analysis of the South Yorkshire cohort (Table 6) was used to describe GP attendance conditional on age, sex, BMI, ethnicity, and health outcomes. The estimated number of GP visits was multiplied by 4 to reflect the annual number of visits per year. In the probabilistic sensitivity analysis the parameters of the South Yorkshire negative binomial model are sampled from a multivariate normal distribution, using the mean estimates described in Table 6 and covariance matrix in Table 7.

Table 6: GP attendance reported in the South Yorkshire Cohort (N= 18,437)

|  | Mean | Standard error | Uncertainty Distribution |
| --- | --- | --- | --- |
| Age | 0.0076 | 0.0005 | MULTIVARIATE NORMAL |
| Male | -0.1495 | 0.0159 | MULTIVARIATE NORMAL |
| BMI | 0.0110 | 0.0015 | MULTIVARIATE NORMAL |
| Ethnicity (Non-white) | 0.2620 | 0.0375 | MULTIVARIATE NORMAL |
| Heart Disease | 0.2533 | 0.0289 | MULTIVARIATE NORMAL |
| Depression | 0.6127 | 0.0224 | MULTIVARIATE NORMAL |
| Osteoarthritis | 0.2641 | 0.0238 | MULTIVARIATE NORMAL |
| Diabetes | 0.2702 | 0.0278 | MULTIVARIATE NORMAL |
| Stroke | 0.1659 | 0.0474 | MULTIVARIATE NORMAL |
| Cancer | 0.2672 | 0.0414 | MULTIVARIATE NORMAL |
| Intercept | -0.5014 | 0.0468 | MULTIVARIATE NORMAL |
| Alpha | 0.3423 | 0.0108 | MULTIVARIATE NORMAL |

Table 7: Variance-covariance matrix for GP attendance regression

|  | Age | Male | BMI | Ethnicity (Non-white) | Heart Disease | Depression | Osteoarthritis | Diabetes | Stroke | Cancer | Intercept | Alpha |
| --- | --- | --- | --- | --- | --- | --- | --- | --- | --- | --- | --- | --- |
| Age | 0.0000 |  |  |  |  |  |  |  |  |  |  |  |
| Male | 0.0000 | 0.0003 |  |  |  |  |  |  |  |  |  |  |
| BMI | 0.0000 | 0.0000 | 0.0000 |  |  |  |  |  |  |  |  |  |
| Ethnicity (Non-white) | 0.0000 | 0.0000 | 0.0000 | 0.0014 |  |  |  |  |  |  |  |  |
| Heart Disease | 0.0000 | 0.0000 | 0.0000 | 0.0000 | 0.0008 |  |  |  |  |  |  |  |
| Depression | 0.0000 | 0.0000 | 0.0000 | 0.0000 | 0.0000 | 0.0005 |  |  |  |  |  |  |
| Osteoarthritis | 0.0000 | 0.0000 | 0.0000 | 0.0000 | 0.0000 | 0.0000 | 0.0006 |  |  |  |  |  |
| Diabetes | 0.0000 | 0.0000 | 0.0000 | 0.0000 | -0.0001 | 0.0000 | 0.0000 | 0.0008 |  |  |  |  |
| Stroke | 0.0000 | 0.0000 | 0.0000 | 0.0000 | -0.0002 | -0.0001 | 0.0000 | -0.0001 | 0.0022 |  |  |  |
| Cancer | 0.0000 | 0.0000 | 0.0000 | 0.0000 | 0.0000 | 0.0000 | 0.0000 | 0.0000 | -0.0001 | 0.0017 |  |  |
| Intercept | 0.0000 | 0.0000 | -0.0001 | -0.0002 | 0.0002 | 0.0000 | 0.0002 | 0.0003 | 0.0000 | 0.0001 | 0.0022 |  |
| Alpha | 0.0000 | 0.0000 | 0.0000 | 0.0000 | 0.0000 | 0.0000 | 0.0000 | 0.0000 | 0.0000 | 0.0000 | 0.0000 | 0.0010 |

# Longitudinal Trajectories of Metabolic Risk Factors

Two separate sets of statistical analyses of longitudinal cohort studies were used to describe metabolic trajectories for individuals in the model. An analysis of the Whitehall II cohort study (6) was developed to describe correlated longitudinal changes in metabolic risk factors for individuals aged 60 years and younger. An analysis of the Englished Longitudinal Study of Ageing was used to describe trajectories for individuals aged 61 and over. The transition point of 61 years was found to be the age at which there were more data observations for participants in ELSA compared with Whitehall. A summary for each set of metabolic trajectory models are provided below.

## Whitehall II Data Analysis

Changes in BMI, latent blood glucose, total cholesterol, HDL cholesterol and SBP were estimated from statistical analysis of the Whitehall II cohort. The growth factors for all 5 risk factors were estimated using parallel latent growth modelling. This enabled the growth factors for BMI to be implemented as covariates for the growth processes of glycaemia, systolic blood pressure, and total cholesterol^[[1]](#footnote-1)^. The structural assumptions of the analysis are described in more detail below.

In the Whitehall II data analysis it was assumed that individuals have an underlying level of glycaemia, which cannot be observed but can be measured by HbA1c, FPG, and 2-hour glucose. This underlying propensity for diabetes is referred to as latent glycaemia. The statistical model estimated the unobservable latent glycaemia, and from this identified associations with test results for HbA1c, FPG, and 2-hour glucose. The longitudinal changes in BMI, glycaemia, SBP, total cholesterol and HDL cholesterol could then be estimated through statistical analysis.

These growth factors are conditional on several individual characteristics including age, sex, ethnicity, smoking, family history of CVD, and family history of type 2 diabetes. We related the effect of changes in BMI to changes in glycaemia, SBP and total cholesterol. However, if an intervention is known to be effective in reducing BMI and the other metabolic risk factors, the Whitehall II model is adjusted to temporarily remove the indirect effect of the intervention through BMI. This ensures that the effectiveness of the intervention is not over-estimated. Unobservable heterogeneity between individual growth factors not explained by patient characteristics was incorporated into the growth models as random error terms. Correlation between the random error terms for glycaemia, total cholesterol, HDL cholesterol and systolic blood pressure was estimated from the Whitehall II cohort. This means that in the simulation, an individual with a higher growth rate for glycaemia was more likely to have a higher growth rate of total cholesterol and SBP.

An advantage of this parallel growth analysis is that it was able to estimate the effect of growth in BMI on the other metabolic risk factors. The statistical analysis also described the correlation between changes in glycaemia, SBP, total cholesterol and HDL cholesterol. As a consequence, the growth factor random error terms were not assumed to be independent and were sampled from a multivariate normal distribution$\boldsymbol{\upsilon}\sim N(0,\Omega)$. Estimates for the covariance matrix are derived from the covariance estimates reported in the statistical analysis.

The baseline observations for BMI, HbA1c, SBP, cholesterol and HDL cholesterol were extracted from the Health Survey for England 2014 in order to simulate a representative sample of the UK population. The predicted intercept for these metabolic risk factors was estimated using the Whitehall II analysis to give population estimates of the individuals’ starting values, conditional on their characteristics. The difference between the simulated and observed baseline risk factors was taken to estimate the individuals’ random deviation from the population expectation. The individual random error in the slope trajectory was sampled from a conditional multivariate normal distribution to allow correlation between the intercept and slope random errors.

Figure 2: Path analysis of final statistical analysis of the Whitehall II cohort

## ELSA Data Analysis

Changes in BMI, HbA1c, systolic blood pressure, total cholesterol and HDL cholesterol were estimated from a statistical analysis of the ELSA cohort. The changes with age were estimated using independently estimate random coefficient growth models in Stata 13. The growth trajectory models for the metabolic risk factor were estimated under the statistical framework of growth curve modelling (GCM) (13). GCM is an approach to using longitudinal data to estimate shape and rate of change over time. GCM was chosen because it can allow modelling of variability in participants fixed and slope parameters. The growth factors for the metabolic risk factors were assumed to vary between individuals to allow unobservable random effects to describe the heterogeneity in intercept and slope parameters. Assessment of the data indicated that there was significant variance in the intercept (risk factor starting value) and slope (change in risk factor over time) for all metabolic risks. The growth factor models without covariates were specified as.

$$Y_{ij}=\left( \beta_{1}+\zeta_{1j} \right)+\left( \beta_{2}+\zeta_{2j} \right)t_{ij}+\epsilon_{ij}$$

Where Y describes the observed metabolic risk factor for individual i at time j, $\beta_{1}$ is the population mean intercept, and $\beta_{2}$ population mean slope. The random factors $\zeta_{1j}$ and $\zeta_{2j}$ describe the random variability across individuals in the intercept and slopes respectively. The statistical models were weighted for selection bias using nurse visit weights for BMI and systolic blood pressure and blood sample weights for Total Cholesterol, HDL Cholesterol and HbA1c supplied by the ELSA dataset to improve the representativeness of the analysis for an English population.

The model assumed that BMI growth was quadratic with age, due to trends observed in the data and in other cohorts (14). HbA1c, systolic blood pressure, total cholesterol and HDL cholesterol were assumed to be linear with time. All model intercepts and slopes were adjusted for sex, smoking status, deprivation and ethnicity. Anti-hypertensive treatment was included as an additional covariate in the systolic blood pressure model. Unfortunately data on statin were not available for any wave except wave 6, therefore we did not include this as a covariate in the analysis. Covariates were included in the final model if the variable was statistical significant with a p-value less than 0.1.

## BMI Trajectory

At baseline, BMI estimates from the HSE determine an individual’s BMI. If the individual is aged 60 years or less annual changes in BMI are calculated from the Whitehall II study based on population average changes for the individual and a sampled random coefficient factor. From ages 61 and over the ELSA BMI statistical model is used estimate their older age trajectory in BMI. New random coefficient growth factors are estimated based on the covariance structure of the ELSA random intercept and slope. As a consequence, current BMI status is informative in determining the future trajectory of BMI.

## Glycaemic Trajectory in Non-Diabetics/undiagnosed Diabetes

At baseline, HbA1c estimates for HbA1c are used to determine an individual’s HbA1c and glycaemic status. For individuals aged 60 years or less the Whitehall II study is used to estimate annual changes in HbA1c, and through latent glycaemia FPG, and 2-hr glucose observations. In the Whitehall II analysis we assume that changes in latent glycaemia have a quadratic relationship with time. The Whitehall II models allow random coefficient factors for growth in glycaemia for an individual and measurement error in test results according to estimated parameters from the Whitehall II analysis. For individuals aged 61 and over the ELSA statistical model is used to estimate an individuals linear changes in HbA1c every year. Random coefficient growth factors are re-estimated using the bivariate covariance structure from the ELSA HbA1c growth model. It is not possible to estimate FPG and 2-hr glucose using the ELSA statistical models.

## HbA1c trajectory in type 2 diagnosed diabetics

Following a diagnosis of diabetes in the simulation all individuals experience an initial fall in HbA1c due to changes in diet and lifestyle as observed in the UKPDS trial (15). We have estimated the expected change in HbA1c conditional on HbA1c at diagnosis by fitting a simple linear regression to three aggregate outcomes reported in the study. These showed that the change in HbA1c increases for higher HbA1c scores at diagnosis. The regression parameters to estimate change in HbA1c are reported in Table 8.

Table 8: Estimated change in HbA1c following diabetes diagnosis

|  | Mean | Standard error |
| --- | --- | --- |
| Change in HbA1c Intercept | -2.99 | .048 |
| HbA1c at baseline | 0.55 | 0.40 |

After this initial reduction in HbA1c the longitudinal trajectory of HbA1c is estimated using the UKPDS outcomes model (15) rather than the Whitehall II statistical analysis. The UKPDS dataset is made up of a newly diagnosed diabetic population. As part of the UKPDS Outcomes model, longitudinal trial data were analysed using a random effects model. The coefficients of the model are reported in Table 9.

Table 9: Coefficient estimates for HbA1c estimated from UKPDS data

|  | Mean Coefficient | Coefficient standard error |
| --- | --- | --- |
| Intercept | -0.024 | 0.017 |
| Log transformation of year since diagnosis | 0.144 | 0.009 |
| Binary variable for year after diagnosis | -0.333 | 0.05 |
| HbA1c score in last period | 0.759 | 0.004 |
| HbA1c score at diagnosis | 0.085 | 0.004 |

The model can be used to predict HbA1c over time from the point of diagnosis. The model suggests that HbA1c increases with time. A graph illustrating change in HbA1c over time from two different HbA1c levels at diagnosis is illustrated in Figure 3.

Figure 3: Trajectory of HbA1c estimated from UKPDS longitudinal model

##

## Total Cholesterol and HDL Cholesterol Trajectories in Individuals not receiving Statins

At baseline, an individual’s total and HDL cholesterol is determined from the HSE 2014 data. In the simulation, individuals aged 60 years and younger have annual changes in total and HDL cholesterol according to the estimates from the statistical analysis of the Whitehall II cohort. The slope of total and HDL cholesterol are assumed to be linear with time. These growth factors are estimated in the model to be conditional on cholesterol at baseline, age at baseline, sex, and an error parameter to reflect unobservable variability in growth trajectories between individuals. As with latent glycaemia, changes in total cholesterol are also influenced by the trajectory of BMI. For individuals aged 61 and over the ELSA statistical models for Total and HDL cholesterol are used to estimate annual change in cholesterol. As individuals transition between the trajectory models the random coefficient factors are updated allowing current observations to inform the trajectories in Total and HDL cholesterol.

## Total Cholesterol and HDL Cholesterol Trajectories in Individuals receiving Statins

During the simulation process, individuals are prescribed statins to reduce their risk of cardiovascular disease. It is assumed within the model that the statins are effective in reducing an individual’s total cholesterol, and an average effect is applied to all patients receiving statins. A recent HTA reviewed the literature on the effectiveness and cost-effectiveness of statins in individuals with acute coronary syndrome (16). This report estimated the change in LDL cholesterol for four statin treatments and doses compared with placebo from a Bayesian meta-analysis. The analysis estimated a reduction in LDL cholesterol of -1.45 for simvastatin. This estimate was used to describe the effect of statins in reducing total cholesterol. It was assumed that the effect was instantaneous upon receiving statins and maintained as long as the individual receives statins. It was also assumed that individuals receiving statins no longer experienced annual changes in total cholesterol. HDL cholesterol was assumed constant over time if patients receive statins.

Non-adherence to statin treatment is a common problem. Two recent HTAs reviewed the literature on continuation and compliance with statin treatment. They both concluded that there was a lack of adequate reporting, but that the proportion of patients fully compliant with treatment appears to decrease with time, particularly in the first 12 months after initiating treatment, and can fall below 60% after five years (11, 16). Although a certain amount of non-compliance is included within trial data, clinical trials are not considered to be representative of continuation and compliance in general practice. A yearly reduction in statin compliance used in the HTA analysis is reported in Table 10. It is based on the published estimate of compliance for the first five years of statin treatment for primary prevention in general clinical practice (16). Compliance declines to a minimum of 65% after five years of treatment. It is assumed that there is no further drop after five years.

Table 10: Proportion of patients assumed to be compliant with statin treatment, derived from Table 62 in (16)

| Year after statin initiation | 1 | 2 | 3 | 4 | 5 |
| --- | --- | --- | --- | --- | --- |
| Proportion compliant | 0.8 | 0.7 | 0.68 | 0.65 | 0.65 |

In the simulation, we assume in the base case that only 65% of individuals initiate statins when they are deemed eligible. However those that initiate statins remain on statins for their lifetime. Those who refuse statins may be prescribed them again at a later date.

## SBP Trajectories in Individuals Not receiving Anti-hypertensive treatment

At baseline an individual’s SBP is determined from the HSE 2014 data. In the simulation, individuals’ aged 60 and younger experience SBP changes every year according to the estimates from the statistical analysis of the Whitehall II cohort. The annual change in SBP is assumed to be linear with time. The growth factors are estimated in the model to be conditional on SBP at baseline, age at baseline, sex, ethnicity, family history of cardiovascular disease, smoking and an error parameter to reflect unobservable variability in growth trajectories between individuals. From ages 61 onwards the ELSA statistical model for systolic blood pressure is used to estimate annual changes. The random coefficient factors are updated using the bivariate covariance matrix for intercept and slope factors.

## SBP Trajectories in Individuals receiving anti-hypertensive treatment

During the simulation process, if individuals are identified as having SBP higher than 160mm Hg, or SBP higher than 140mm Hg with comorbid diabetes, cardiovascular disease, or 10 year risk of cardiovascular disease greater than 20%, they will be prescribed anti-hypertensive treatment in line with the National Institute for Health and Care Excellence (NICE) guidelines (17). The change in SBP following initiation of calcium channel blockers was estimated in a meta-analysis of anti-hypertensive treatments (18). This study identified an average change in SBP of -8.4 for monotherapy with calcium channel blockers. In the simulation model it is assumed that this reduction in SBP is maintained for as long as the individual receives anti-hypertensive treatment. Once an individual is receiving anti-hypertensive treatment it is assumed that their SBP is stable and does not change over time, which implicitly assumes that patients continue to be well managed for their hypertension. For simplicity we do not explicitly simulate treatment switching. The assumed zero flat trajectory in systolic blood pressure whilst receiving anti-hypertensives is supported by the analysis of the ELSA dataset in which self-reported use of anti-hypertensives was included as a covariate for age-related change in systolic blood pressure. The analysis found that most of the observed changes in systolic blood pressure were removed if individuals were taking anti-hypertensives.

## Metabolic Risk factor screening

We assume that individuals eligible for anti-hypertensive treatment or statins will be identified through opportunistic screening if they meet certain criteria and attend the GP for at least one visit in the simulation period.

1. Individuals with a history of cardiovascular disease;
2. Individuals with a major microvascular event (foot ulcer, blindness, renal failure or amputation);
3. Individuals with diagnosed diabetes;
4. Individuals identified with Impaired Glucose Regulation;
5. Individuals with systolic blood pressure greater than 160mmHg.

The base case has been designed to represent a health system with moderate levels of screening for hypertension, and dyslipidaemia. Alternative assumptions for more or less intensive opportunistic screening can be assumed.

## Diagnosis and Treatment Initiation

It is assumed that there are three, non-mutually exclusive outcomes from the vascular checks or opportunistic screening. Firstly, that the patient receives statins to reduce cardiovascular risk. Secondly, that the patient has high blood pressure and should be treated with anti-hypertensive medication. The following threshold estimates were used to determine these outcomes.

1. Statins are initiated if the individual has greater than or equal to 20% 10 year CVD risk estimated from the QRISK2 2012 algorithm (19).
2. Anti-hypertensive treatment is initiated if systolic blood pressure is greater than 160. If the individual has a history of CVD, diabetes or a CVD risk >20%, the threshold for systolic blood pressure is 140 (17).

## Diabetes Diagnosis

The HSE repeatedly demonstrates that a large proportion of the English population have a HbA1c test score above 6.5%, and do not report a diagnosis of diabetes. Therefore, it is important to allow some undiagnosed cases of diabetes to persist in the model. The process for diagnosing diabetes in the model utilises data from the QDiabetes 2018 risk algorithm (20) and the HbA1c longtiduinal trajectory models. In each cyle an individual is diagnosed with Diabetes if they are assessed to experience a diabetes diagnosis in the next 10 years using the QDiabetes alrogithm and have two HbA1c tests scores above 6.5 in that period.

The model estimates the QDiabetes score, with HBA1c covariates, for undiagnosed individuals within the population in each annual cycle. This score describe the individuals 10 year probability of being diagnosed with diabetes conditional on demographic, socioeconomic, ethnicity, medical history and HbA1c score. The QDiabetes 2018 model was chosen to describe individuals risk of diabetes in the model to describe differential risks across individuals.

The QDiabetes 2018 risk equation can be used to calculate the probability of a cardiovascular event including: coronary heart disease (angina or myocardial infarction), stroke, or transient ischaemic attacks, fatality due to cardiovascular disease. The equation estimates the probability of a cardiovascular event in the next period conditional on the coefficients listed in

Table 12. The equation for the probability of an event in the next period is calculated as

$$p\left( Y=1 \right)=1-{S(1)}^{\theta}$$

$$\theta=\sum\beta X$$

The probability of an event is calculated from the survival function at 1 year raised to the power of $\boldsymbol{\theta}$, where $\boldsymbol{\theta}$ is the sum product of the coefficients reported in

Table 11

Table 12 multiplied by the individual’s characteristics. Underlying survival curves for men and women were extracted from the QDiabetes 2018 open source file. Mean estimates for the continuous variables were also reported in the open source files.

Table 11: Coefficients from the 2018 QDiabetes risk equation and estimated standard errors

| Estimated coefficients adjusting for individual characteristics | | | | | | | | | |
| --- | --- | --- | --- | --- | --- | --- | --- | --- | --- |
|  | Women | | Men | |  | Women | | Men | |
| Covariates | Mean | Standard error | Mean | Standard error | Interaction terms | Mean | Standard error | Mean | Standard error |
| White | 0.0000 |  | 0.0000 |  | Age1*former smoker | -0.8125 | 0.035 | -1.0013 | 0.776 |
| Indian | 0.5991 |  | 0.6757 |  | Age1*light smoker | -0.9085 | 0.066 | -0.8916 | 3.341 |
| Pakistani | 0.7832 |  | 0.8315 |  | Age1*moderate smoker | -1.8558 | 0.231 | -1.7075 | 3.075 |
| Bangladeshi | 1.1947 |  | 1.0969 |  | Age1*Heavy smoker | 0.6023 | 0.308 | 0.4507 | 3.529 |
| Other Asian | 0.7142 |  | 0.7682 |  | Age1*AF | -0.0345 | 0.922 | -0.1085 | 1.406 |
| Caribbean | 0.1195 |  | 0.2090 |  | Age1*renal disease | -0.2728 | 0.528 | -0.6141 | 3.403 |
| Black African | 0.0137 |  | 0.3809 |  | Age1*hypertension | 25.4412 | 0.450 | 27.6706 | 6.793 |
| Chinese | 0.5709 |  | 0.3424 |  | Age1*Diabetes | -6.8076 | 0.369 | -7.4006 | 2.558 |
| Other | 0.1709 |  | 0.2205 |  | Age1*BMI | 0.0005 | 0.617 | 0.0002 | 0.654 |
| Non-smoker | 0.0000 |  | 0.0000 |  | Age1*family history CVD | 0.0009 | 0.050 | 0.0007 | 3.584 |
| Former smoker | 0.0658 |  | 0.1159 |  | Age1*SBP | 0.0023 | 0.003 | 0.0014 | 0.030 |
| Light smoker | 0.1458 |  | 0.1462 |  | Age1*Townsend | -0.0043 | 0.007 | -0.0012 | 0.510 |
| Moderate smoker | 0.1526 |  | 0.1078 |  | Age2*former smoker | 0.0001 |  | 0.0002 |  |
| Heavy smoker | 0.3079 |  | 0.1985 |  | Age2*light smoker | 0.0004 |  | 0.0005 |  |
| Age 1* | 3.5655 |  | 4.0193 |  | Age2*moderate smoker | -0.0523 |  | -0.0592 |  |
| Age 2* | -0.0056 |  | -0.0048 |  | Age2*Heavy smoker | 0.0141 |  | 0.0156 |  |
| BMI* | 2.5043 |  | 0.8183 |  | Age2*AF | -0.8125 |  | -1.0013 |  |
| Ratio Total / HDL chol | -0.0429 |  | -0.1256 |  | Age2*renal disease | -0.9085 |  | -0.8916 |  |
| SBP | 8.7368 |  | 8.0512 |  | Age2*hypertension | -1.8558 |  | -1.7075 |  |
| Townsend | -0.0782 |  | -0.1465 |  | Age2*Diabetes | 0.6023 |  | 0.4507 |  |
| AF | 0.0359 |  | 0.0252 |  | Age2*BMI | -0.0345 |  | -0.1085 |  |
| Rheumatoid arthritis | 0.5498 |  | 0.4554 |  | Age2*family history CVD | -0.2728 |  | -0.6141 |  |
| Renal disease | 0.1687 |  | 0.1382 |  | Age2*SBP | 25.4412 |  | 27.6706 |  |
| Hypertension | 0.1644 |  | 0.1455 |  | Age2*Townsend | -6.8076 |  | -7.4006 |  |
| Diabetes | 1.1250 |  | 0.2596 |  |  |  |  |  |  |
| Family history of CVD | 0.2891 |  | 0.2852 |  |  |  |  |  |  |
| AF Atrial Fibrillation CVD Cardiovascular disease SBP systolic blood pressure * covariates transformed with fractional polynomials | | | | | | | | | |

#

# Comorbid Outcomes and Mortality

In every model cycle individuals within the model are evaluated to determine whether they have a clinical event, including mortality, within the cycle period. In each case the simulation estimates the probability that an individual has the event and uses a random number draw to determine whether the event occurred.

## Cardiovascular Disease

### First Cardiovascular event

Several statistical models for cardiovascular events were identified in a review of economic evaluations for diabetes prevention (21). The UKPDS outcomes model (22), Framingham risk equation (23) and QRISK2 (24) have all been used in previous models to estimate cardiovascular events. The Framingham risk equation was not adopted because, unlike the QRISK2 model, it is not estimated from a UK population. The UKPDS outcomes model would be ideally suited to estimate the risk of cardiovascular disease in a population diagnosed with type 2 diabetes. Whilst this is an important outcome of the cost-effectiveness model, there was concern that it would not be representative of individuals with normal glucose tolerance or impaired glucose regulation. Recent analyses show that the UKPDS over-predicts cardiovascular outcomes in newly diagnosed diabetes patients (25). It was important that reductions in cardiovascular disease risk in these populations were represented to capture the population-wide benefits of public health interventions. The QRISK2 model was selected for use in the cost-effectiveness model because it is a validated model of cardiovascular risk in a up to date UK population and could be used to generate probabilities for diabetic and non-diabetic populations. We considered using the UKPDS outcomes model specifically to estimate cardiovascular risk in patients with type 2 diabetes. However, it would not be possible to control for shifts in absolute risk generated by the different risk scores due to different baselines and covariates. This would lead to some individuals experiencing counterintuitive and favourable shifts in risk after onset of type 2 diabetes. Therefore, we decided to use diabetes as a covariate adjustment to the QRISK2 model to ensure that the change in individual status was consistent across individuals.

The probability of the first cardiovascular event is estimated from the QRISK2 predicted model of cardiovascular disease (20). The QRISK2 is a validated risk prediction algorithm to identify individuals at high risk of cardiovascular disease. The algorithm was developed from UK data and incorporates social deprivation and ethnicity. We accessed the coefficients and algorithm from the online QRISK website (26). The QRISK2 equation estimates the probability of a cardiovascular event in the next 10 years conditional on ethnicity, smoking status, age, BMI, ratio of total/HDL cholesterol, Townsend score, atrial fibrillation, rheumatoid arthritis, renal disease, hypertension, diabetes, and family history of cardiovascular disease. Data on all these variables was available from the HSE 2014.

### First Cardiovascular event calibration

In version 4 of the model the QRISK2 algorithm was updated with 2014 risk coefficients. The 2014 version did not report the 1 year survival function. For the 2014 version we estimated the 1 year parameter using a simple calibration process to match the CVD incidence from a randomized controlled trial of a weight loss programme (the Weight-loss programme Referrals for Adults in Primary care [WRAP] trial) after 5 years of follow-up, to the simulated incidence of CVD events (27). A simple calibration approach was taken to identify a parameter estimate for the 1 year survival parameter for men and women that would simulate the estimated CVD incidence reported from the 5 year follow-up data within a 2% margin or error. This data was based on cardiovascular events to include, MI, percutaneous coronary intervention (PCI), Experienced bypass (CABG), invasive cardiovascular procedure, Transient Ischaemic Attack or stroke reported in GP records.

At 5 years follow-up GP records for CVD history were available for for 859 women, and 408 men. Of those 14 and 18 had experienced a CVD event respectively. For the calibration were identified survival parameters for men and women that would generate an incidence of 3.26 per 1000 person years for women and 8.824 per 1000 person years for men in the brief intervention simulation. These parameters were estimated with a simple iterative process simulating 20,000 randomly selected indiviudals. The final estimates were tested against other random samples. Starting values were selected from using the survival parameters reported in the QRISK 2012 source code.

### First Cardiovascular event inputs

Table 12 reports the coefficient estimates for the QRISK2 algorithm. The standard errors were not reported within the open source code. Where possible, standard errors were imputed from a previous publication of the risk equation (28). Coefficients that were not reported in this publication were assumed to have standard errors of 20%.

Table 12: Coefficients from the 2014 QRISK2 risk equation and estimate standard errors

|  | | Estimated coefficients adjusting for individual characteristics | | | | | | | | | | | | |
| --- | --- | --- | --- | --- | --- | --- | --- | --- | --- | --- | --- | --- | --- | --- |
|  | | | Women | | | Men | |  | | Women | | | Men | |
| Covariates | Mean | | | Standard error | Mean | | Mean | Interaction terms | Mean | | Standard error | Mean | | Standard error |
| White | 0.0000 | | | 0.0000 | 0.0000 | | 0.0000 | Age1*smoke1 | 0.6891 | | 0.035 | 0.9244 | | 0.776 |
| Indian | 0.2672 | | | 0.0537 | 0.2785 | | 0.0425 | Age1*smoke2 | 0.6943 | | 0.066 | 1.9598 | | 3.341 |
| Pakistani | 0.7148 | | | 0.0698 | 0.6068 | | 0.0547 | Age1*smoke3 | -1.6952 | | 0.231 | 2.9994 | | 3.075 |
| Bangladeshi | 0.3703 | | | 0.1073 | 0.7104 | | 0.0727 | Age1*smoke4 | -1.2150 | | 0.308 | 5.0371 | | 3.529 |
| Other Asian | 0.2074 | | | 0.1071 | 0.8626 | | 0.0845 | Age1*AF | -3.5855 | | 0.922 | 8.2354 | | 1.406 |
| Caribbean | -0.1744 | | | 0.0619 | 3.8735 | | 0.0641 | Age1*renal disease | -3.0767 | | 0.528 | -3.9747 | | 3.403 |
| Black African | -0.3272 | | | 0.1275 | 0.1347 | | 0.1094 | Age1*hypertension | -4.0295 | | 0.450 | 7.8738 | | 6.793 |
| Chinese | -0.2201 | | | 0.1721 | -0.1558 | | 0.1538 | Age1*Diabetes | -3.3145 | | 0.369 | 5.0624 | | 2.558 |
| Other | -0.2090 | | | 0.0793 | -3.7728 | | 0.0734 | Age1*BMI 1 | -5.5934 | | 0.617 | 33.5438 | | 0.654 |
| Non-smoker | 0.0000 | | | 0.0000 | 0.1526 | | 0.0000 | Age1*BMI 2 | 64.3636 | | 0.050 | -129.9767 | | 3.584 |
| Former smoker | 0.1947 | | | 0.0152 | 0.0132 | | 0.0108 | Age1*family history CVD | 0.8605 | | 0.050 | 1.9280 | | 3.584 |
| Light smoker | 0.6229 | | | 0.0220 | 0.0644 | | 0.0166 | Age1*SBP | -0.0509 | | 0.003 | 0.0523 | | 0.030 |
| Moderate smoker | 0.7406 | | | 0.0178 | 1.4235 | | 0.0148 | Age1*Townsend | 0.1519 | | 0.007 | -0.1731 | | 0.510 |
| Heavy smoker | 0.9134 | | | 0.0194 | 0.3021 | | 0.0143 | Age2*smoke1 | -0.1765 | | 0.001 | -0.0034 | | 1.594 |
| Age 1 | 3.8735 | | |  | -17.6226 | |  | Age2*smoke2 | -0.2324 | | 0.000 | -0.0051 | | 4.737 |
| Age 2 | 0.1347 | | |  | 0.0242 | |  | Age2*smoke3 | 0.2734 | | 0.002 | 0.0003 | | 4.627 |
| BMI 1* | -0.1558 | | | 0.0423 | 1.7320 | | 0.0299 | Age2*smoke4 | 0.1433 | | 0.003 | 0.0031 | | 5.373 |
| BMI2 * | -3.7728 | | |  | -7.2312 | |  | Age2*AF | 0.4987 | | 0.010 | 0.0073 | | 2.890 |
| Ratio Total / HDL chol | 0.1526 | | | 0.0044 | 0.1751 | | 0.0022 | Age2*renal disease | 0.4393 | | 0.007 | -0.0262 | | 5.654 |
| SBP | 0.0132 | | | 0.0045 | 0.0102 | | 0.0046 | Age2*hypertension | 0.6904 | | 0.005 | 0.0086 | | 3.763 |
| Townsend | 0.0644 | | | 0.0068 | 0.0298 | | 0.0048 | Age2*Diabetes | 0.4865 | | 0.004 | -0.0002 | | 0.193 |
| AF | 1.4235 | | | 0.0310 | 0.9891 | | 0.1018 | Age2*BMI 1 | 1.5223 | | 0.007 | 0.0812 | | 2.110 |
| Rheumatoid arthritis | 0.3021 | | | 0.0319 | 0.2542 | | 0.0445 | Age2*BMI 2 | -12.7413 | |  | -0.2559 | |  |
| Renal disease | 0.8615 | | | 0.0639 | 0.7950 | | 0.0702 | Age2*family history CVD | -0.2757 | | 0.001 | -0.0057 | | 5.321 |
| Hypertension | 0.5889 | | | 0.0115 | 0.6229 | | 0.0112 | Age2*SBP | 0.0074 | | 0.000 | -0.0001 | | 0.058 |
| Diabetes | 1.1350 | | | 0.0199 | 0.9373 | | 0.0175 | Age2*Townsend | -0.0487 | | 0.000 | -0.0011 | | 0.601 |
| Family history of CVD | 0.5134 | | | 0.0122 | 0.5923 | | 0.0111 |  |  | |  |  | |  |
| AF Atrial Fibrillation CVD Cardiovascular disease SBP systolic blood pressure * covariates transformed with fractional polynomials | | | | | | | | | | | | | | |

The QRISK2 risk equation can be used to calculate the probability of a cardiovascular event including: coronary heart disease (angina or myocardial infarction), stroke, or transient ischaemic attacks, fatality due to cardiovascular disease. The equation estimates the probability of a cardiovascular event in the next period conditional on the coefficients listed in

Table 12. The equation for the probability of an event in the next period is calculated as

$$p\left( Y=1 \right)=1-{S(1)}^{\theta}$$

$$\theta=\sum\beta X$$

The probability of an event is calculated from the survival function at 1 year raised to the power of $\boldsymbol{\theta}$, where $\boldsymbol{\theta}$ is the sum product of the coefficients reported in

Table 12 multiplied by the individual’s characteristics. Underlying survival curves for men and women were extracted from the QRISK2 open source file. Mean estimates for the continuous variables were also reported in the open source files.

We modified the QRISK2 assumptions regarding the relationship between IGR, diabetes and cardiovascular disease. Firstly, we assumed that individuals with HbA1c>6.5 have an increased risk of cardiovascular disease even if they have not received a formal diagnosis. Secondly, risk of cardiovascular disease was assumed to increase with HbA1c for test results greater than 6.5 to reflect observations from the UKPDS that HbA1c increases the risk of MI and Stroke (22). Thirdly, prior to type 2 diabetes (HbA1c>6.5) HbA1c is linearly associated with cardiovascular disease. A study from the EPIC Cohort has found that a unit increase in HbA1c increases the risk of coronary heart disease by a hazard ratio of 1.25, after adjustment for other risk factors (29). We apply this risk ratio to linearly increase risk above the mean HBA1c observed in the HSE 2011 cohort. A linear risk reduction was applied at HbA1c levels below the HSE mean.

The QRISK2 algorithm identifies which individuals experience a cardiovascular event but does not specify the nature of the event. The nature of the cardiovascular event was determined independently. A targeted search of recent Health Technology appraisals of cardiovascular disease was performed to identify a model for the progression of cardiovascular disease following a first event. A Health Technology Assessment (HTA) assessing statins gives age and sex specific distributions of CVD, which were used to assign all QRISK2 events (11). Table 13 reports the probability of cardiovascular outcomes by age and gender. Stakeholders suggested that there may be different relationships between the risk factors and the different types of CVD (e.g. hypertension is more of a risk factor for stroke). However, we decided not to incorporate these differential factors in evaluating the risk of cardiovascular event types into the model due to a lack of evidence.

Table 13: The probability distribution of cardiovascular events by age and gender

|  | Age | Stable angina | Unstable angina | MI rate | Fatal CHD | TIA | Stroke | Fatal CVD |
| --- | --- | --- | --- | --- | --- | --- | --- | --- |
| Men | 45-54 | 0.307 | 0.107 | 0.295 | 0.071 | 0.060 | 0.129 | 0.030 |
|  | 55-64 | 0.328 | 0.071 | 0.172 | 0.086 | 0.089 | 0.206 | 0.048 |
|  | 65-74 | 0.214 | 0.083 | 0.173 | 0.097 | 0.100 | 0.270 | 0.063 |
|  | 75-84 | 0.191 | 0.081 | 0.161 | 0.063 | 0.080 | 0.343 | 0.080 |
|  | 85+ | 0.214 | 0.096 | 0.186 | 0.055 | 0.016 | 0.351 | 0.082 |
| Women | 45-54 | 0.325 | 0.117 | 0.080 | 0.037 | 0.160 | 0.229 | 0.054 |
|  | 55-64 | 0.346 | 0.073 | 0.092 | 0.039 | 0.095 | 0.288 | 0.067 |
|  | 65-74 | 0.202 | 0.052 | 0.121 | 0.081 | 0.073 | 0.382 | 0.090 |
|  | 75-84 | 0.149 | 0.034 | 0.102 | 0.043 | 0.098 | 0.464 | 0.109 |
|  | 85+ | 0.136 | 0.029 | 0.100 | 0.030 | 0.087 | 0.501 | 0.117 |

### Subsequent Cardiovascular events

After an individual has experienced a cardiovascular event, it is not possible to predict the transition to subsequent cardiovascular events using QRISK2. As with assigning first CVD events, the probability of subsequent events was estimated from the HTA evaluating statins (11). This study reported the probability of future events conditional on the nature of the previous event. Table 14 reports an example of the probabilities within a year of transitioning from stable angina, unstable angina, myocardial infarction (MI), transient ischemic attack (TIA) or stroke for individuals by age group.

Table 14: Probability of cardiovascular event conditional on age and status of previous event (column1)

|  | Stable angina | Unstable angina 1 | Unstable angina 2 | MI 1 | MI 2 | TIA | Stroke 1 | Stroke 2 | CHD death | CVD death |
| --- | --- | --- | --- | --- | --- | --- | --- | --- | --- | --- |
| Age 45 |  |  |  |  |  |  |  |  |  |  |
| Stable angina | 0.9946 | 0.0013 | 0 | 0.0032 | 0 | 0 | 0 | 0 | 0.0009 | 0 |
| Unstable angina (1^st^ yr) | 0 | 0 | 0.9127 | 0.0495 | 0 | 0 | 0 | 0 | 0.0362 | 0.0016 |
| Unstable angina (subsequent) | 0 | 0 | 0.9729 | 0.0186 | 0 | 0 | 0 | 0 | 0.0081 | 0.0004 |
| MI (1^st^ yr) | 0 | 0 | 0 | 0.128 | 0.8531 | 0 | 0.0015 | 0 | 0.0167 | 0.0007 |
| MI (subsequent) | 0 | 0 | 0 | 0.0162 | 0.978 | 0 | 0.0004 | 0 | 0.0052 | 0.0002 |
| TIA | 0 | 0 | 0 | 0.0016 | 0 | 0.9912 | 0.0035 | 0 | 0.0024 | 0.0013 |
| Stroke (1^st^ yr) | 0 | 0 | 0 | 0.0016 | 0 | 0 | 0.0431 | 0.9461 | 0.0046 | 0.0046 |
| Stroke (subsequent) | 0 | 0 | 0 | 0.0016 | 0 | 0 | 0.0144 | 0.9798 | 0.0021 | 0.0021 |
| Age 55 |  |  |  |  |  |  |  |  |  |  |
| Stable angina | 0.9874 | 0.0029 | 0 | 0.0062 | 0 | 0 | 0 | 0 | 0.0035 | 0 |
| Unstable angina (1^st^ yr) | 0 | 0 | 0.8859 | 0.0497 | 0 | 0 | 0 | 0 | 0.0617 | 0.0027 |
| Unstable angina (subsequent) | 0 | 0 | 0.9548 | 0.0348 | 0 | 0 | 0 | 0 | 0.01 | 0.0004 |
| MI (1^st^ yr) | 0 | 0 | 0 | 0.1152 | 0.8483 | 0 | 0.0032 | 0 | 0.0319 | 0.0014 |
| MI (subsequent) | 0 | 0 | 0 | 0.0179 | 0.9716 | 0 | 0.001 | 0 | 0.0091 | 0.0004 |
| TIA | 0 | 0 | 0 | 0.0031 | 0 | 0.9626 | 0.0181 | 0 | 0.0092 | 0.007 |
| Stroke (1^st^ yr) | 0 | 0 | 0 | 0.0031 | 0 | 0 | 0.0459 | 0.9288 | 0.0111 | 0.0111 |
| Stroke (subsequent) | 0 | 0 | 0 | 0.0031 | 0 | 0 | 0.0186 | 0.9685 | 0.0049 | 0.0049 |
| Age 65 |  |  |  |  |  |  |  |  |  |  |
| Stable angina | 0.976 | 0.006 | 0 | 0.011 | 0 | 0 | 0 | 0 | 0.007 | 0 |
| Unstable angina (1^st^ yr) | 0 | 0 | 0.8435 | 0.0488 | 0 | 0 | 0 | 0 | 0.1031 | 0.0046 |
| Unstable angina (subsequent) | 0 | 0 | 0.9244 | 0.0632 | 0 | 0 | 0 | 0 | 0.0119 | 0.0005 |
| MI (1^st^ yr) | 0 | 0 | 0 | 0.1019 | 0.8287 | 0 | 0.0068 | 0 | 0.0599 | 0.0027 |
| MI (subsequent) | 0 | 0 | 0 | 0.0185 | 0.9634 | 0 | 0.0022 | 0 | 0.0152 | 0.0007 |
| TIA | 0 | 0 | 0 | 0.0055 | 0 | 0.9174 | 0.0423 | 0 | 0.0185 | 0.0163 |
| Stroke (1^st^ yr) | 0 | 0 | 0 | 0.0055 | 0 | 0 | 0.0481 | 0.8944 | 0.026 | 0.026 |
| Stroke (subsequent) | 0 | 0 | 0 | 0.0055 | 0 | 0 | 0.0223 | 0.9514 | 0.0104 | 0.0104 |
| Age 75 |  |  |  |  |  |  |  |  |  |  |
| Stable angina | 0.9681 | 0.0091 | 0 | 0.0158 | 0 | 0 | 0 | 0 | 0.007 | 0 |
| Unstable angina (1^st^ yr) | 0 | 0 | 0.7789 | 0.0466 | 0 | 0 | 0 | 0 | 0.1671 | 0.0074 |
| Unstable angina (subsequent) | 0 | 0 | 0.8733 | 0.1122 | 0 | 0 | 0 | 0 | 0.0139 | 0.0006 |
| MI (1^st^ yr) | 0 | 0 | 0 | 0.0874 | 0.7849 | 0 | 0.0141 | 0 | 0.1088 | 0.0048 |
| MI (subsequent) | 0 | 0 | 0 | 0.0178 | 0.953 | 0 | 0.0047 | 0 | 0.0235 | 0.001 |
| TIA | 0 | 0 | 0 | 0.008 | 0 | 0.8588 | 0.0828 | 0 | 0.0185 | 0.0319 |
| Stroke (1^st^ yr) | 0 | 0 | 0 | 0.008 | 0 | 0 | 0.0446 | 0.8302 | 0.0586 | 0.0586 |
| Stroke (subsequent) | 0 | 0 | 0 | 0.008 | 0 | 0 | 0.0246 | 0.9262 | 0.0206 | 0.0206 |
| Age 85 |  |  |  |  |  |  |  |  |  |  |
| Stable angina | 0.9601 | 0.0122 | 0 | 0.0207 | 0 | 0 | 0 | 0 | 0.007 | 0 |
| Unstable angina (1^st^ yr) | 0 | 0 | 0.6873 | 0.0425 | 0 | 0 | 0 | 0 | 0.2587 | 0.0115 |
| Unstable angina (subsequent) | 0 | 0 | 0.7878 | 0.1955 | 0 | 0 | 0 | 0 | 0.016 | 0.0007 |
| MI (1^st^ yr) | 0 | 0 | 0 | 0.0711 | 0.7053 | 0 | 0.0278 | 0 | 0.1875 | 0.0083 |
| MI (subsequent) | 0 | 0 | 0 | 0.016 | 0.9394 | 0 | 0.0091 | 0 | 0.034 | 0.0015 |
| TIA | 0 | 0 | 0 | 0.0104 | 0 | 0.838 | 0.0961 | 0 | 0.0185 | 0.037 |
| Stroke (1^st^ yr) | 0 | 0 | 0 | 0.0104 | 0 | 0 | 0.0446 | 0.702 | 0.1215 | 0.1215 |
| Stroke (subsequent) | 0 | 0 | 0 | 0.0104 | 0 | 0 | 0.0252 | 0.8894 | 0.0375 | 0.0375 |

### Congestive Heart Failure

The review of previous economic evaluations of diabetes prevention cost-effectiveness studies found that only a small number of models had included congestive heart failure as a separate outcome. Discussion with the stakeholder group identified that the UKPDS Outcomes model would be an appropriate risk model for congestive heart failure in type 2 diabetes patients. However, it was suggested that this would not be an appropriate risk equation for individuals with normal glucose tolerance or impaired glucose tolerance. The Framingham risk equation was suggested as an alternative. As described above, switching from the framgingam risk score to the UKPDS was not possible due to differences in covariate selection. The main limitations of this equation is that it is quite old, based on a non-UK population, and include diabetes as a discrete health state rather than on a continuous scale.

Congestive heart failure was included as a separate cardiovascular event because it was not included as an outcome of the QRISK2. The Framingham Heart Study has reported logistic regressions to estimate the 4 year probability of congestive heart failure for men and women (30). The equations included age, diabetes diagnosis, BMI and systolic blood pressure to adjust risk based on individual characteristics. We used this risk equation to estimate the probability of congestive heart failure in the SPHR diabetes prevention model. Table 15 describes the covariates for the logit models to estimate the probability of congestive heart failure in men and women.

Table 15: Logistic regression coefficients to estimate the 4-year probability of congestive heart failure from the Framingham study

| Variables | Units | Regression  Coefficient | OR (95% CI) | P |
| --- | --- | --- | --- | --- |
| Men | | | | |
| Intercept |  | -9.2087 |  |  |
| Age | 10 y | 0.0412 | 1.51 (1.31-1.74) | <.001 |
| Left ventricular hypertrophy | Yes/no | 0.9026 | 2.47 (1.31-3.77) | <.001 |
| Heart rate | 10 bpm | 0.0166 | 1.18 (1.08-1.29) | <.001 |
| Systolic blood pressure | 20 mm Hg | 0.00804 | 1.17 (1.04-1.32) | 0.007 |
| Congenital heart disease | Yes/no | 1.6079 | 4.99 (3.80-6.55) | <.001 |
| Valve disease | Yes/no | 0.9714 | 2.64 (1.89-3.69) | <.001 |
| Diabetes | Yes/no | 0.2244 | 1.25 (0.89-1.76) | 0.2 |
| Women | | | | |
| Intercept |  | -10.7988 |  |  |
| Age | 10 y | 0.0503 | 1.65 (1.42-1.93) | <.001 |
| left ventricular hypertrophy | Yes/no | 1.3402 | 3.82 (2.50-5.83) | <.001 |
| Heart rate | 100 cL | 0.0105 | 1.11 (1.01-1.23) | 0.03 |
| Systolic blood pressure | 10 bpm | 0.00337 | 1.07 (0.96-1.20) | 0.24 |
| congenital heart disease | 20 mm Hg | 1.5549 | 4.74 (3.49-6.42) | <.001 |
| Valve disease | Yes/no | 1.3929 | 4.03 (2.86-5.67) | <.001 |
| Diabetes | Yes/no | 1.3857 | 4.00 (2.78-5.74) | <.001 |
| BMI | kg/m2 | 0.0578 | 1.06 (1.03-1.09) | <.001 |
| Valve disease and diabetes | Yes/no | -0.986 | 0.37 (0.18-0.78) | 0.009 |
| *OR indicates odds ratio; CI, confidence interval; LVH, left ventricular hypertrophy; CHD, congenital heart disease; and BMI, body mass index. Predicted probability of heart failure can be calculated as: p = 1/(1+exp(-xbeta)), where xbeta = Intercept + Sum (of regression coefficient*value of risk factor) | | | | |

Many of the risk factors included in this risk equation were not simulated in the diabetes model, therefore they could not be included in the model to predict CHD. We adjusted the baseline odds of CHD to reflect the expected prevalence of these symptoms in a UK population.

The proportion of the UK population with left ventricular hypertrophy was assumed to be 5% in line with previous analyses of the Whitehall II cohort (31). The heart rate for men was assumed to be 63.0bpm and for women 65.6bpm based on data from previous Whitehall II cohort analyses (32). The prevalence of congenital heart disease was estimated from an epidemiology study in the North of England. The study reports the prevalence of congenital heart disease among live births which was used to estimate the adult prevalence (33). This may over-estimate the prevalence, because the life expectancy of births with congenital heart disease is reduced compared with the general population. However, given the low prevalence it is unlikely to impact on the results. The prevalence of valve disease was estimated from the Echocardiographic Heart of England Screening study (34).

Using the estimated population values we adjusted the intercept values to account for the population risk in men and women. This resulted in a risk equation with age, systolic blood pressure, diabetes (diabetes diagnosis or HbA1c>6.5), and BMI in women to describe the risk of congestive heart failure for the policy analysis model.

## Microvascular Complications

The review of previous economic evaluations identified that the UKPDS data was commonly used to estimate the incidence of microvascular complications (21). This data has the advantage of being estimated from a UK diabetic population. Given that the events described in the UKPDS outcomes model are indicative of late stage microvascular complications, we did not believe it was necessary to seek an alternative model that would be representative of an impaired glucose tolerance population.

We adopted a simple approach to modelling microvascular complications. We used both versions of the UKPDS Outcomes model to estimate the occurrence of major events relating to these complications, including renal failure, amputation, foot ulcer, and blindness (22). These have the greatest cost and utility impact compared with earlier stages of microvascular complications, so are more likely to have an impact on the SPHR diabetes prevention outcomes.

As a consequence, we assumed that microvascular complications only occur in individuals with HbA1c>48 mmol/mol (6.5%). Whilst some individuals with hyperglycaemia (HbA1c>42 mmol/mol [6.0%]) may be at risk of developing microvascular complications, it is unlikely that they will progress to renal failure, amputation or blindness before a diagnosis of diabetes. Importantly, we did not assume that only individuals who have a formal diagnosis of diabetes are at risk of these complications. This allows us to incorporate the costs of undetected diabetes into the simulation.

The UKPDS includes four statistical models to predict foot ulcers, amputation with no prior ulcer, amputation with prior ulcer and a second amputation (22). In order to simplify the simulation of neuropathy outcomes we consolidated the models for first amputation with and without prior ulcer into a single equation. The parametric survival models were used to generate estimates of the cumulative hazard in the current and previous period. From which the probability of organ damage being diagnosed was estimated.

|  | $p\left( Death \right)=1-exp(H\left( t \right)-H\left( t-1 \right))$ |  |
| --- | --- | --- |

The functional form for the microvascular models included exponential and Weibull.

#### Retinopathy

We used the UKPDS outcomes model v2 to estimate the incidence of blindness in individuals with HbA1c>48 mmol/mol (6.5%) (22). The exponential model assumes a baseline hazard $\lambda$, which can be calculated from the model coefficients reported in Table 16 and the individual characteristics for $\boldsymbol{X}$.

$$\lambda=exp\left( \beta_{0}\mathbf{+}\boldsymbol{X}\boldsymbol{\beta}_{\boldsymbol{k}} \right)$$

Table 16: Parameters of the UKPDS2 Exponential Blindness survival model

|  | Mean coefficient | Standard error | Modified mean coefficient |
| --- | --- | --- | --- |
| Lambda | -11.607 | 0.759 | -10.967 |
| Age at diagnosis | 0.047 | 0.009 | 0.047 |
| HbA1c | 0.171 | 0.032 | 0.171 |
| Heart rate | 0.080 | 0.039 |  |
| SBP | 0.068 | 0.032 | 0.068 |
| White Blood Count | 0.052 | 0.019 |  |
| CHF History | 0.841 | 0.287 | 0.841 |
| IHD History | 0.0610 | 0.208 | 0.061 |
| SBP Systolic Blood Pressure; CHF Congestive Heart Failure; IHD Ischaemic Heart Disease | | | |

The age at diagnosis coefficient was multiplied by age in the current year if the individual had not been diagnosed with diabetes, and by the age at diagnosis if the individual had received a diagnosis.

The expected values for the risk factors not included in the SPHR model (heart rate and white blood count) were taken from Figure 3 of the UKPDS publication in which these are described (22). Assuming these mean values, it was possible to modify the baseline risk without simulating heart rate and white blood cell count.

#### Neuropathy

We used the UKPDS outcomes model v2 to estimate the incidence of ulcer and amputation in individuals with HbA1c>48 mmol/mol (6.5%) (22). The parameters of the ulcer and first amputation models are reported in Table 17.

Table 17: Parameters of the UKPDS2 Exponential model for Ulcer, Weibull model for first amputation with no prior ulcer and exponential model for 1^st^ amputation with prior ulcer

|  | Ulcer | | 1^st^ Amputation no prior ulcer | | 1^st^ Amputation prior ulcer | | 2^nd^ Amputation | |
| --- | --- | --- | --- | --- | --- | --- | --- | --- |
|  | Logistic | | Weibull | | Exponential | | Exponential | |
|  | Mean | Standard error | Mean | Standard error | Mean | Standard error | Mean | Standard error |
| Lambda | -11.295 | 1.130 | -14.844 | 1.205 | -0.881 | 1.39 | -3.455 | 0.565 |
| Rho |  |  | 2.067 | 0.193 |  |  |  |  |
| Age at diagnosis | 0.043 | 0.014 | 0.023 | 0.011 | -0.065 | 0.027 |  |  |
| Female | -0.962 | 0.255 | -0.0445 | 0.189 |  |  |  |  |
| Atrial fibrillation |  |  | 1.088 | 0.398 |  |  |  |  |
| BMI | 0.053 | 0.019 |  |  |  |  |  |  |
| HbA1c | 0.160 | 0.056 | 0.248 | 0.042 |  |  | 0.127 | 0.06 |
| HDL |  |  | -0.059 | 0.032 |  |  |  |  |
| Heart rate |  |  | 0.098 | 0.050 |  |  |  |  |
| MMALB |  |  | 0.602 | 0.180 |  |  |  |  |
| PVD | 0.968 | 0.258 | 1.010 | 0.189 | 1.769 | 0.449 |  |  |
| SBP |  |  | 0.086 | 0.043 |  |  |  |  |
| WBC |  |  | 0.040 | 0.017 |  |  |  |  |
| Stroke History |  |  | 1.299 | 0.245 |  |  |  |  |

The exponential model assumes a baseline hazard $\lambda$, which can be calculated from the model coefficients reported in Table 17 and the individual characteristics for $\boldsymbol{X}$.

$$\lambda=exp\left( \beta_{0}\mathbf{+}\boldsymbol{X\beta} \right)$$

The Weibull model for amputation assumes a baseline hazard:

$$h\left( t \right)=\rho t^{\rho-1}exp(\lambda)$$

where $\lambda$is also conditional on the coefficients and individual characteristics at time t.

The logistic model for ulcer is described below.

$$\Pr\left( y=1 | \mathbf{X} \right)=\frac{exp(\boldsymbol{X\beta})}{1+exp(\boldsymbol{X\beta}))}$$

The ulcer and amputation models include a number of covariates that were not included in the simulation. As such it was necessary to adjust the statistical models to account for these measures. We estimated a value for the missing covariates and added the value multiplied by the coefficient to the baseline hazard.

The expected values for the risk factors not included in the SPHR model (heart rate, white blood count, micro-/macroalbuminurea, peripheral vascular disease and atrial fibrillation) were taken from Figure 3 of the UKPDS publication in which these are described (22). In the ulcer model we assumed that 2% of the population had peripheral vascular disease.

The amputation risk model with a history of ulcer was not included in the simulation, but was used to estimate an additional log hazard ratio to append onto the amputation model without a history of ulcer. The log hazard was estimated for each model assuming the same values for other covariates. The difference in the log hazard between the two models was used to approximate the log hazard ratio for a history of ulcer in the amputation model (10.241). The final model specifications are reported in Table 18.

Table 18: Coefficients estimates for Ulcer and 1^st^ Amputation

|  | Ulcer | | 1^st^ Amputation | | 2^nd^ Amputation | |
| --- | --- | --- | --- | --- | --- | --- |
|  | Logistic | | Weibull | | Exponential | |
|  | Mean | Standard error | Mean | Standard error | Mean | Standard error |
| Lambda | -11.276 | 1.13 | -13.954 | 1.205 | -3.455 | 0.565 |
| Rho |  |  | 2.067 | 0.193 |  |  |
| Age at Diagnosis | 0.043 | 0.014 | 0.023 | 0.011 |  |  |
| Female | -0.962 | 0.255 | -0.445 | 0.189 |  |  |
| BMI | 0.053 | 0.019 |  |  |  |  |
| HbA1c | 0.160 | 0056 | 0.248 | 0.042 | 0.127 | 0.06 |
| HDL |  |  | -0.059 | 0.032 |  |  |
| Stroke |  |  | 1.299 | 0.245 |  |  |
| Foot Ulcer |  |  | 10.241 |  |  |  |

#### Nephropathy

We used the UKPDS outcomes model v1 to estimate the incidence of renal failure in individuals with HbA1c>48 mmol/mol (6.5%) (15). Early validation analyses identified that the UKPDS v2 model substantially overestimated the incidence of renal failure in the SPHR model. The Weibull model for renal failure assumes a baseline hazard:

$$h\left( t \right)=\rho t^{\rho-1}exp(\lambda)$$

where $\lambda$is also conditional on the coefficients and individual characteristics at time t. The parameters of the renal failure risk model are reported in Table 19.

Table 19: Parameters of the UKPDS2 Weibull renal failure survival model

|  | Mean | Standard error |
| --- | --- | --- |
| Lambda | -10.016 | 0.939 |
| Shape parameter | 1.865 | 0.387 |
| SBP | 0.404 | 0.106 |
| BLIND History | 2.082 | 0.551 |

##

## Cancer

The conceptual model identified breast cancer and colorectal cancer risk as being related to BMI. However, these outcomes were not frequently included in previous cost-effectiveness models for diabetes prevention. Discussion with stakeholders identified the EPIC Norfolk epidemiology cohort study as a key source of information about cancer risk in a UK population. Therefore, we searched publications from this cohort to identify studies reporting the incidence of these risks. In order to obtain the best quality evidence for the relationship between BMI and cancer risk we searched for a recent systematic review and meta-analysis using key terms ‘Body Mass Index’ and ‘Cancer’, filtering for meta-analysis studies.

### Breast cancer

Incidence rates for breast cancer in the UK were estimated from the European Prospective Investigation of Cancer (EPIC) cohort. This is a large multi-centre cohort study looking at diet and cancer. In 2004 the UK incidence of breast cancer by menopausal status was reported in a paper from this study investigating the relationship between body size and breast cancer (35). The estimates of the breast cancer incidence in the UK are reported in Table 20.

Table 20: UK breast cancer incidence

|  | Number of Cases | Person Years | Mean BMI | Incidence Rate of per person-year | Standard error | Reference |
| --- | --- | --- | --- | --- | --- | --- |
| UK pre-menopause | 102 | 103114.6 | 24 | 0.00099 | 0.00009 | (35) |
| UK post-menopause | 238 | 84214.6 | 24 | 0.00283 | 0.00004 | (35) |

A large meta-analysis that included 221 prospective observational studies has reported relative risks of cancers per unit increase in BMI, including breast cancer by menopausal status (36). We included a risk adjustment in the model so that individuals with higher BMI have a higher probability of pre-and post-menopausal breast cancer (36). In the simulation we adjusted the probability of breast cancer according to the difference in the individual’s BMI and the average BMI reported in the EPIC cohort. The relative risk and confidence intervals per 5mg/m^2^ increase in BMI are reported in Table 21.

Table 21: Relative risk of Breast cancer by BMI

|  | Mean Relative risk | 2.5^th^ Confidence Interval | 97.5^th^ Confidence Interval | Reference |
| --- | --- | --- | --- | --- |
| UK pre-menopause | 0.89 | 0.84 | 0.94 | (36) |
| UK post-menopause | 1.09 | 1.04 | 1.14 | (36) |

### Colorectal cancer

Incidence rates for colorectal cancer in the UK were reported from the European Prospective Investigation of Cancer (EPIC) cohort. The UK incidence of colorectal cancer is reported by gender in a paper from this study investigating the relationship between body size and colon and rectal cancer (37). The estimates of the colorectal cancer incidence are reported in Table 22.

Table 22: UK colorectal cancer incidence

|  | Number of Cases | Person Years | Mean Age | Mean BMI | Incidence Rate of per person-year | Standard error | Reference |
| --- | --- | --- | --- | --- | --- | --- | --- |
| Male | 125 | 118468 | 53.1 | 25.4 | 0.00106 | 0.0001 | (37) |
| Female | 145 | 277133 | 47.7 | 24.5 | 0.00052 | 0.0002 | (37) |

The risk of colorectal cancer has been linked to obesity. We included a risk adjustment in the model to reflect observations that the incidence of breast cancer is increased in individuals with higher BMI. A large meta-analysis that included 221 prospective observational studies has reported relative risks of BMI and cancers, including colon cancer by gender (36). We selected linear relative risk estimates estimated from pooled European and Australian populations. In the simulation we adjusted the incidence of colorectal cancer by adjusting the probability of colorectal cancer by the difference in the individual’s BMI and the average BMI reported in the EPIC cohort. The relative risk and confidence intervals per 5mg/m^2^ increase in BMI are reported in Table 23.

Table 23: Relative risk of colon cancer by BMI

|  | Mean Relative risk | 2.5^th^ Confidence Interval | 97.5^th^ Confidence Interval | Reference |
| --- | --- | --- | --- | --- |
| Male | 1.21 | 1.18 | 1.24 | (36) |
| Female | 1.04 | 1.00 | 1.07 | (36) |

##

## Osteoarthritis

Stakeholders suggested that diabetes and BMI should be included as independent risk factors for osteoarthritis. Osteoarthritis had not been included as a health state in previous cost-effectiveness models. The stakeholder group requested that BMI and diabetes be included as risk factors for osteoarthritis based on recent evidence (38). A search for studies using key words ‘Diabetes’, ‘Osteoarthritis’ and ‘Cohort Studies’ did not identify a UK based study with diabetes and body mass index included as independent covariates in the risk model. Therefore, the Italian study was used in the model.

A study from the Bruneck cohort, a longitudinal study of inhabitants of a town in Italy reported diabetes and BMI as independent risk factors for osteoarthritis (38).

The cohort may not be representative of a UK cohort. However, the individuals are from a European country, the study has a large sample size and has estimated the independent effects of BMI and diabetes on the risk of osteoarthritis. No UK based studies identified in our searches met these requirements. The data used to estimate the incidence of osteoarthritis is reported in Table 24. We did not identify any studies that described diabetes risk on a continuous scale.

Table 24: Incidence of osteoarthritis and estimated risk factors

|  | No cases | Person years | Mean BMI | Incidence rate | Standard error | Reference |
| --- | --- | --- | --- | --- | --- | --- |
| No diabetes | 73 | 13835 | 24.8 | 0.0053 | 0.0006 | (38) |
|  | Hazard ratio | 2.5th | 97.5th |  |  | Reference |
| HR Diabetes | 2.06 | 1.11 | 3.84 |  |  | (38) |
| HR BMI | 1.076 | 1.023 | 1.133 |  |  | (38) Personal communication |

## Depression

Depression was not included as a health state in previous cost-effectiveness models for diabetes prevention. However, a member of the stakeholder group identified that a relationship between diabetes and depression was included in the CORE diabetes treatment model (39). Therefore, the references used in this model were used.

Depression was included as a health state in the model. However, the severity of depression was not modelled. Some individuals enter the simulation with depression at baseline according to individual responses in the Health Survey for England 2014 questionnaire. Depression is described in the simulation as a chronic state from which individuals do not completely remit. We did not estimate the effect of depression on the longitudinal changes for BMI, glycaemia, SBP and cholesterol. As a consequence, it was not possible to relate the impact of depression to the incidence of diabetes and cardiovascular risk.

In the simulation, individuals can develop depression in any cycle of the model. The baseline incidence of depression among all individuals without a history of depression was estimated from a study examining the bidirectional association between depressive symptoms and type 2 diabetes (40). Although the study was not from a UK population, the US cohort included ethnically diverse men and women aged 45 to 84 years. We assumed that diagnosis of diabetes and/or CVD increased the incidence of depression in individuals who do not have depression at baseline. We identified a method for inflating risk of depression for individuals with diabetes from the US cohort study described above (40). The risk of depression in individuals who have had a stroke was also inflated according to a US cohort study (41). Odds of depression and odds ratios for inflated risk of depression due to diabetes or stroke are presented in Table 25.

Table 25: Baseline incidence of depression

| Baseline Risk of depression | | | |
| --- | --- | --- | --- |
|  | Mean | Standard error |  |
| Depression cases in NGT | 336 |  |  |
| Person years | 9139 |  |  |
| Odds of depression | 0.0382 | 0.002 |  |
| Log odds of depression | -3.266 |  |  |
| Inflated risk for Diabetes | | | |
|  | Mean | 2.5th CI | 97.5th CI |
| Odds ratio of diabetes | 1.52 | 1.09 | 2.12 |
| Log odds ratio of diabetes | 0.419 |  |  |
| Inflate risk of stroke | | | |
| Odds ratio of stroke | 6.3 | 1.7 | 23.2 |
| Log odds ratio stroke | 1.8406 |  |  |
| NGT Normal Glucose Tolerance | | | |

## Dementia

The risk dementia diagnosis is estimated from risk models estimated from the THIN database (42). The THIN dementia risk score uses data from The Health Improvement Network (THIN) database from across the UK. Routinely collected data was used to predict 5-year risk of recorded diagnosis of Dementia for those aged 60-79 and 80+. The sample size is large and the risk scores are representative of the United Kingdom and diagnosis practices between 2000-2011. The disadvantage of these risk scores are the relatively short follow-up of patients, the low predictive power of the older risk score, and narrow scope to predict dementia diagnosis but not dementia onset.

The parameters for the THIN 60-79 year old and 80-99 risk models are reported in Table 26.

Table 26: THIN dementia risk models

| THIN 60-79 Risk Score | | | THIN 80-99 Risk Score | | |
| --- | --- | --- | --- | --- | --- |
| Parameter label | mean | Standard error | Parameter label | mean | Standard error |
| Baseline hazard | 0.9969 |  | Baseline hazard | -0.9277 |  |
| Age | 0.2092 | 0.0047 | Age | 0.055 | 0.0041 |
| Age^2^ | -0.0034 | 0.0003 | Age^2^ | -0.005 | 0.0010 |
| Female | 0.1285 | 0.0278 | Female | 0.16 | 0.0286 |
| Calendar Year | 0.0448 | 0.0050 | Calendar Year | 0.074 | 0.0056 |
| Townsend quintile 2 | 0.0134 | 0.0390 | BMI | -0.05 | 0.0066 |
| Townsend quintile 3 | 0.1179 | 0.0392 | Anti-hypertensives | -0.249 | 0.0265 |
| Townsend quintile 4 | 0.2018 | 0.0402 | Systolic Blood Pressure | -0.006 | 0.0010 |
| Townsend quintile 5 | 0.2255 | 0.0447 | Lipid ratio | 0.042 | 0.0495 |
| BMI | -0.0616 | 0.0038 | Past Smoker | -0.178 | 0.0281 |
| BMI^2^ | 0.0025 | 0.0003 | Smoker | -0.134 | 0.0485 |
| Anti-hypertensives | -0.1320 | 0.0296 | Alcohol Porblems | 0.256 | 0.1352 |
| Past Smoker | -0.0679 | 0.0301 | Diabetes | 0.183 | 0.0413 |
| Smoker | -0.0866 | 0.0415 | Stroke | 0.242 | 0.0332 |
| Alcohol problems | 0.4435 | 0.0799 | Atrial Fibrillation | 0.057 | 0.0383 |
| Diabetes | 0.2867 | 0.0417 | Depression | 0.4 | 0.0332 |
| Depression | 0.8336 | 0.0325 | Anxiety | 0.136 | 0.0520 |
| Stroke | 0.5772 | 0.0394 | NSAIDs use | -0.157 | 0.0408 |
| Atrial Fibrillation | 0.2207 | 0.0514 | Aspirin use | 0.092 | 0.0281 |
| Aspirin use | 0.2528 | 0.0326 |  |  |  |

For the SPHR prevention model the 5-year Dementia risk was transformed into 1 year individual probabilities. The Dementia risk scores include fixed and time-varying patient characteristics. As a consequence, it is not possible to use standard methods of transforming probabilities over different time-horizons (43). We used a simple calibration technique to modify the baseline hazard to reflect simulated changes in the populations risk profile over 5 years. We calibrate the simulated 5 year incidence of dementia against the predicted incidence for each age group in the THIN database .

For each risk model we simulated 20,000 randomly sampled patients aged 60-79 and 80-95 in 50 model runs. For each sample we repeated simulations multiple times, in each simulation the baseline hazard was adjusted until the incidence of Dementia equalled the THIN risk score prediction based on baseline characteristics. The baseline hazard adjustment was estimated by averaging the adjustments needed for each of the 20 simulation runs to match the THIN prediction. The calibration was designed to calibrate to the predicted incidence, rather than the reported incidence from the THIN dataset to account for any differences in the baseline characteristics of the THIN data and HSE sample. For example, the mean age for the development cohort of the 60-79 model were 65.6, whereas the mean simulated ages was 70. Age is an important predictor of Dementia incidence so it is important to adjust for differences in baseline age between the observed and simulated data.

The THIN database reports a crude incidence of 1.88 per 1,000 persons years for 60-79 and 16.53 per 1,000 person years for ages 80-99 (column 2 Table 27). The 5 year risk score for individuals sampled from the Health Survey for England reports a 5 year incidence of 0.00255 and 0.01523 (column 3 Table 27). Using the adjustment factor identified by calibration (column 5 Table 27), we simulated a 5 year incidence of 0.00255 and 0.01510 (column 4 Table 27). The adjustment factor is applied to the baseline hazards of the THIN dementia risk scores.

Table 27: Dementia incidence rates used to derive the adjustment factor

|  | 5 year crude incidence from QResearch | 5 year risk score (SD) for Health Survey for England population | 5 year simulated incidence (SD) with adjustment factor | Adjustment factor (SE) |
| --- | --- | --- | --- | --- |
| THIN risk model 60-79 | 0.00188 | 0.00255 | 0.00255 | 7.628 (0.104) |
| THIN risk model 80+ | 0.01653 | 0.01523 | 0.01510 | 4.557 (0.020) |

### Dementia Diagnosis

A Swedish registry reporting MMSE scores at dementia diagnosis was identified (44). This study reported a mean MMSE score at diagnosis of 21.2 (SD 5.2). This data was used to generate MMSE scores at diagnosis in the model because it reflects cognitive function in a cohort diagnosed in routine care. In order to generate heterogeneity in cognitive function at diagnosis a Gamma distribution was fitted to this mean and standard distribution and patients MMSE score at diagnosed was sampled from this distribution. Sampled value outside the limits of the MMSE score, assuming a maximum score at diagnosis of 25, were re-sampled from a uniform distribution within these limits. The resulting distribution was compared against summary data from CFAS for an incident cohort to validate the simulated MMSE scores against this dataset (45).


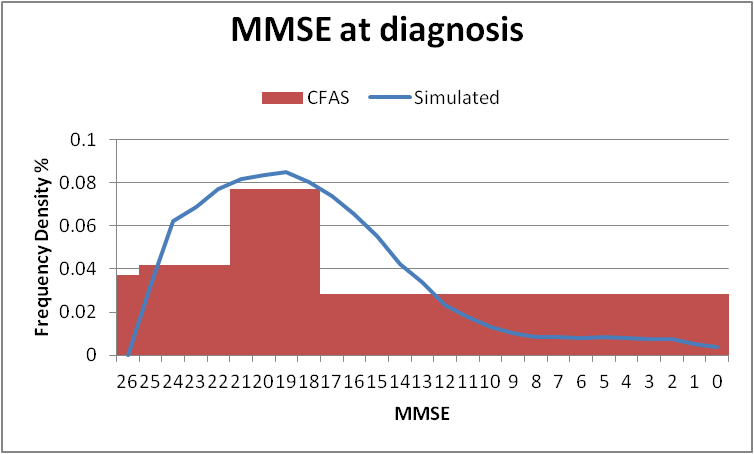


### Disease Progression

Dementia disease progression was characterized by a deterioration in MMSE score. This relatively simple characterization of the disease is sufficient to capture major cost escalations and quality of life deterioration. A more complex structure that explicity models insitutionalisation was considered (46). However, it was concluded that the data on insitutionalisation was out of date. Therefore, the modelling structure was aligned to the most up to date cost estimates for dementia (47).

Changes in MMSE score over time were estimated using data from a recent cost-effectiveness model for Donepezil (48). Although the data are from Canada and are relatively old, the sample size is large and model specification allows for detailed characterization of MMSE decline. The regression allows rate of change in MMSE to be conditional on age at baseline and includes splines to describe a different rate of change at different levels of MMSE. For example, the decline in MMSE slows as the score declines below 9.

## Mortality

### Cardiovascular Mortality

Cardiovascular mortality is included as an event within the QRISK2 (24) and the probability of subsequent cardiovascular events obtained from an HTA assessing statins (11), as described in the Cardiovascular disease section above.

### Cancer Mortality

Cancer mortality rates were obtained from the Office of National statistics (49). The ONS report one and five year net survival rates for various cancer types, by age group and gender. Net survival was an estimate of the probability of survival from the cancer alone. It can be interpreted as the survival of cancer patients after taking into account the background mortality that the patients would have experienced if they had not had cancer.

The age-adjusted 5-year survival rate for breast cancer and colorectal cancer were used to estimate an annual risk of mortality assuming a constant rate of mortality. We assume that the mortality rate does not increase due to cancer beyond 5 years after cancer diagnosis. The five year survival rate for breast cancer is 84.3%, which translated into a 3.37% annual probability of death from breast cancer. The five year survival rate for persons with colorectal cancer is 55.3%, which translated into a 11.16% annual probability of death from colorectal cancer.

### Other cause Mortality (including diabetes and Dementia risk)

Other cause mortality describes the risk of death from any cause except CVD, and cancer. All-cause mortality rates by age and sex were extracted from the 2014 Office of National Statistics life tables (5;7). The mortality statistics report the number of deaths by ICD codes for 5-year age groups. We subtracted the number of cardiovascular disease,diabetes, dementia, breast and colorectal cancer related deaths from the all-cause mortality total to estimate other cause mortality rates by age and sex (Table 23).

Table 28: All cause and derived other cause mortality from the Office of National statistics

|  | All cause | All cause | Other cause | Other cause |  | All cause | All cause | Other cause | Other cause |
| --- | --- | --- | --- | --- | --- | --- | --- | --- | --- |
|  | Men | Women | Men | Women |  | Men | Women | Men | Women |
| 1 | 0.0003 | 0.0003 | 0.0003 | 0.0003 | 51 | 0.0030 | 0.0021 | 0.0022 | 0.0015 |
| 2 | 0.0002 | 0.0001 | 0.0002 | 0.0001 | 52 | 0.0030 | 0.0021 | 0.0022 | 0.0015 |
| 3 | 0.0001 | 0.0001 | 0.0001 | 0.0001 | 53 | 0.0030 | 0.0021 | 0.0022 | 0.0015 |
| 4 | 0.0001 | 0.0001 | 0.0001 | 0.0001 | 54 | 0.0030 | 0.0021 | 0.0022 | 0.0015 |
| 5 | 0.0001 | 0.0001 | 0.0001 | 0.0001 | 55 | 0.0030 | 0.0021 | 0.0022 | 0.0015 |
| 6 | 0.0001 | 0.0001 | 0.0001 | 0.0001 | 56 | 0.0030 | 0.0021 | 0.0022 | 0.0015 |
| 7 | 0.0001 | 0.0001 | 0.0001 | 0.0001 | 57 | 0.0030 | 0.0021 | 0.0022 | 0.0015 |
| 8 | 0.0001 | 0.0001 | 0.0001 | 0.0001 | 58 | 0.0030 | 0.0021 | 0.0022 | 0.0015 |
| 9 | 0.0001 | 0.0001 | 0.0001 | 0.0001 | 59 | 0.0030 | 0.0021 | 0.0022 | 0.0015 |
| 10 | 0.0001 | 0.0001 | 0.0001 | 0.0001 | 60 | 0.0030 | 0.0021 | 0.0022 | 0.0015 |
| 11 | 0.0001 | 0.0001 | 0.0001 | 0.0001 | 61 | 0.0030 | 0.0021 | 0.0022 | 0.0015 |
| 12 | 0.0001 | 0.0001 | 0.0001 | 0.0001 | 62 | 0.0030 | 0.0021 | 0.0022 | 0.0015 |
| 13 | 0.0001 | 0.0001 | 0.0001 | 0.0001 | 63 | 0.0030 | 0.0021 | 0.0022 | 0.0015 |
| 14 | 0.0001 | 0.0001 | 0.0001 | 0.0001 | 64 | 0.0030 | 0.0021 | 0.0022 | 0.0015 |
| 15 | 0.0001 | 0.0001 | 0.0001 | 0.0001 | 65 | 0.0030 | 0.0021 | 0.0022 | 0.0015 |
| 16 | 0.0002 | 0.0001 | 0.0002 | 0.0001 | 66 | 0.0030 | 0.0021 | 0.0022 | 0.0015 |
| 17 | 0.0003 | 0.0001 | 0.0003 | 0.0001 | 67 | 0.0030 | 0.0021 | 0.0022 | 0.0015 |
| 18 | 0.0004 | 0.0002 | 0.0004 | 0.0002 | 68 | 0.0030 | 0.0021 | 0.0022 | 0.0015 |
| 19 | 0.0005 | 0.0002 | 0.0004 | 0.0002 | 69 | 0.0030 | 0.0021 | 0.0022 | 0.0015 |
| 20 | 0.0004 | 0.0002 | 0.0004 | 0.0002 | 70 | 0.0030 | 0.0021 | 0.0022 | 0.0015 |
| 21 | 0.0004 | 0.0002 | 0.0004 | 0.0002 | 71 | 0.0030 | 0.0021 | 0.0022 | 0.0015 |
| 22 | 0.0004 | 0.0002 | 0.0004 | 0.0002 | 72 | 0.0030 | 0.0021 | 0.0022 | 0.0015 |
| 23 | 0.0005 | 0.0002 | 0.0005 | 0.0002 | 73 | 0.0030 | 0.0021 | 0.0022 | 0.0015 |
| 24 | 0.0005 | 0.0002 | 0.0005 | 0.0002 | 74 | 0.0030 | 0.0021 | 0.0022 | 0.0015 |
| 25 | 0.0005 | 0.0002 | 0.0005 | 0.0002 | 75 | 0.0030 | 0.0021 | 0.0022 | 0.0015 |
| 26 | 0.0006 | 0.0002 | 0.0006 | 0.0002 | 76 | 0.0030 | 0.0021 | 0.0022 | 0.0015 |
| 27 | 0.0006 | 0.0003 | 0.0006 | 0.0002 | 77 | 0.0030 | 0.0021 | 0.0022 | 0.0015 |
| 28 | 0.0006 | 0.0003 | 0.0006 | 0.0003 | 78 | 0.0030 | 0.0021 | 0.0022 | 0.0015 |
| 29 | 0.0006 | 0.0003 | 0.0006 | 0.0003 | 79 | 0.0030 | 0.0021 | 0.0022 | 0.0015 |
| 30 | 0.0007 | 0.0003 | 0.0006 | 0.0003 | 80 | 0.0030 | 0.0021 | 0.0022 | 0.0015 |
| 31 | 0.0007 | 0.0004 | 0.0007 | 0.0003 | 81 | 0.0030 | 0.0021 | 0.0022 | 0.0015 |
| 32 | 0.0007 | 0.0004 | 0.0007 | 0.0003 | 82 | 0.0030 | 0.0021 | 0.0022 | 0.0015 |
| 33 | 0.0008 | 0.0005 | 0.0007 | 0.0004 | 83 | 0.0030 | 0.0021 | 0.0022 | 0.0015 |
| 34 | 0.0008 | 0.0005 | 0.0008 | 0.0004 | 84 | 0.0030 | 0.0021 | 0.0022 | 0.0015 |
| 35 | 0.0010 | 0.0005 | 0.0009 | 0.0004 | 85 | 0.0030 | 0.0021 | 0.0022 | 0.0015 |
| 36 | 0.0010 | 0.0006 | 0.0009 | 0.0005 | 86 | 0.0030 | 0.0021 | 0.0022 | 0.0015 |
| 37 | 0.0011 | 0.0006 | 0.0010 | 0.0005 | 87 | 0.0030 | 0.0021 | 0.0022 | 0.0015 |
| 38 | 0.0012 | 0.0007 | 0.0011 | 0.0006 | 88 | 0.0030 | 0.0021 | 0.0022 | 0.0015 |
| 39 | 0.0013 | 0.0008 | 0.0012 | 0.0006 | 89 | 0.0030 | 0.0021 | 0.0022 | 0.0015 |
| 40 | 0.0015 | 0.0008 | 0.0012 | 0.0006 | 90 | 0.0030 | 0.0021 | 0.0022 | 0.0015 |
| 41 | 0.0016 | 0.0009 | 0.0013 | 0.0007 | 91 | 0.0030 | 0.0021 | 0.0022 | 0.0015 |
| 42 | 0.0016 | 0.0010 | 0.0013 | 0.0008 | 92 | 0.0030 | 0.0021 | 0.0022 | 0.0015 |
| 43 | 0.0018 | 0.0011 | 0.0015 | 0.0008 | 93 | 0.0030 | 0.0021 | 0.0022 | 0.0015 |
| 44 | 0.0019 | 0.0012 | 0.0016 | 0.0009 | 94 | 0.0030 | 0.0021 | 0.0022 | 0.0015 |
| 45 | 0.0022 | 0.0013 | 0.0017 | 0.0010 | 95 | 0.0030 | 0.0021 | 0.0022 | 0.0015 |
| 46 | 0.0022 | 0.0014 | 0.0018 | 0.0010 | 96 | 0.0030 | 0.0021 | 0.0022 | 0.0015 |
| 47 | 0.0024 | 0.0016 | 0.0019 | 0.0011 | 97 | 0.0030 | 0.0021 | 0.0022 | 0.0015 |
| 48 | 0.0025 | 0.0017 | 0.0020 | 0.0012 | 98 | 0.0030 | 0.0021 | 0.0022 | 0.0015 |
| 49 | 0.0028 | 0.0018 | 0.0023 | 0.0013 | 99 | 0.0030 | 0.0021 | 0.0022 | 0.0015 |
| 50 | 0.0030 | 0.0021 | 0.0022 | 0.0015 | 100 | 0.0030 | 0.0021 | 0.0022 | 0.0015 |

The rate of other cause mortality by age and sex was treated as the baseline hazard. Following input from stakeholders, an increased risk of mortality was assigned to individuals with diabetes using data from a published meta-analysis (50). This study used data from 820,900 people from 97 prospective studies to calculate hazard ratios for cause-specific death, according to baseline diabetes status (50). Cause of death was separated into vascular disease, cancer and other cause mortality. From this study we estimated that individuals with a diagnosis of diabetes have a fixed increased risk of other cause mortality (Hazard ratio 1.8 (95% CI 1.71-1.9)). The estimates reported in the meta-analysis include increased risk of death from renal disease, therefore mortality from renal disease was not simulated separately to avoid double counting of benefits.

Mortality risk increases with the onset of dementia. As a consequence, all cause mortality was inflated after diagnosis of dementia. The hazard ratio of mortality was estimated from analysis of two United States cohorts (51). Participants were recruited to the studies without known dementia at baseline and received annual clinical evaluation and brain donation at death. The analysis included 2566 persons over 8 years and found a hazard ratio of death with all dementia of 4.54 (CI 3.54-5.83) for ages 75-84 and 2.77 (CI 2.37-3.23) for ages 85 and older . These hazard ratios were applied in the model to all cause mortality to describe mortality at younger ages 60-84 and older 85+ ages.

Mortality rates for individuals with diabetes and undiagnosed diabetes are also at increased risk of mortality, which is applied to all cause mortality. Given the correlation between risk factors of diabetes and dementia, and the high prevalence of multi-comorbidities in later life, it is necessary to adjust mortality risk for individuals with both dementia and diabetes. We believe that applying both mortality hazard ratios would over-estimate the mortality burden in these patients. In the model the higher hazard ratio for Dementia is applied.

# Direct Health Care Costs

At any given time period of the model individuals can have multiple health complications that incur direct healthcare costs. Some of the health states are mutually exclusive; however an individual can accrue multiple complications within the model. Each health state is associated with an average cost, which is accrued by all individuals for every time period for which the state is indicated. Resource use for each comorbidity is added together and no savings are assumed to be made from the use of the same resources for two or more comorbidities for an individual.

In some instances we have adopted costs and prices from old studies. We have inflated all prices and costs to 2020/21 prices using HCHS and NHHCII inflation indices reported in the Personal Social Services Research Unit (PSSRU) (52).

Primary care and community care costs were sought from the Personal Social Services Research Unit (PSSRU) (52), and secondary care costs from UK reference costs (53). Drug costs were obtained from the British National Formulary (54). In most instances costs for long term health outcomes were sought from recent Health Technology Appraisals as this was thought to be the best source of evidence for costs and resource use by disease area in the UK. If an HTA appraisal was not identified, searches for good quality cost-effectiveness analyses for the relevant disease area were conducted to identify the appropriate UK costs.

## GP attendance

The costs of each visit to a General Practitioner were estimated at £39 from the Personal Social Services Research Unit (PSSRU) (52).

Diabetes diagnosis incurred a cost of £16.22 in line with costs used for a previous evaluation of a Diabetes Prevention Programme (3).

Recent guidelines for hypertension have recommended that hypertension be confirmed with ambulatory blood pressure monitoring (ABPM) (17). The cost of ABPM assessment is included in the cost of diagnosis (£45.34) (55), however, we assume that the test does not alter the initial diagnosis.

The cost of identifying individuals to receive statins is assumed to be negligible because cases are detected using existing cardiovascular risk programmes used by the GP.

## Diabetes

We were advised by stakeholders to model a simplified diabetes treatment pathway. It was recommended that a single annual cost of prescriptions be applied to all patients diagnosed with diabetes. Initially we explored this as an option but concluded that the timing of more costly treatments for type 2 diabetes is important because treatment costs will be discounted. The model assesses interventions that lower HbA1c and so have the potential to impact on the level of treatment required.

We decided to implement a three stage treatment regimen as a trade-off between model simplicity and capturing key cost differences between the interventions. At diagnosis all patients are prescribed low cost treatments, such as Metformin and Sulfonylurea. We chose Metformin, 500mg/day to describe the average cost of these medications. If HbA1c increases above a threshold the individual is prescribed the more expensive Gliptins in addition to Metformin. The individual continues to receive Metformin plus Gliptins for a period of time until they require insulin. A summary of unit costs used for diabetes maintenance is detailed in Table 29.

Table 29: Unit costs used for diabetes maintenance

| Resource | Unit cost | Standard error | Source |
| --- | --- | --- | --- |
| Nurse at GP (1 hour) | £44 | 4.4 | (52) |
| Health care assistant (1 hour) | £33 | 3.3 | (52) |
| Urine sample | £1 | 0.1 | (53) |
| Eye screening | £21.71 | NA | (56) |
| HbA1c | £3 | 0.3 | (53) |
| Lipids | £1 | 0.1 | (53) |
| Liver function | £1 | 0.1 | (53) |
| B12 | £1 | 0.1 | (53) |

### Metformin Monotherapy

Cost estimates from the British National Formulary indicate that the cost of Metformin is approximately £25.19 per annum, using a combination of standard and modified release tablets (54). The use of blood glucose self-monitoring strips was described in a recent UK based study in which 36% of patients used monitoring strips at a mean weekly consumption of 3.1 (57) for individuals prescribed Metformin only, at a cost of 18p per strip as reported in the BNF.

Other resource use costs and resource utilisation assumptions for diabetics receiving Metformin monotherapy are detailed in Table 30.

Table 30: Drug costs and resource utilisation costs for low cost diabetes monotherapy

| Resource | Assumption for costs | Unit cost | Source | Inflation | Annual utilisation | Source | Cost per year |
| --- | --- | --- | --- | --- | --- | --- | --- |
| Metformin | 500mg *bid* standard (85% of patients) or modified release (15%) tablets | £25.19 per annum | (54) | 1 | 1 | Assumption | £25.19 |
| Nurse at GP | Nurse advanced per surgery consultation with qualifications | £22 | (52) | 1 | 1 | Stakeholder workshop | £22 |
| Health care assistant | Clinical support worker patient work 10 mins | £5.50 | (52) | 1 | 1 | Stakeholder workshop | £5.50 |
| Urine sample | Biochemistry | £1 | (48) | 1.09 | 1 | Stakeholder workshop | £1.09 |
| Eye screening | Optometrist test 2006 price | £21.71 | (56) | 1 | 1 | Stakeholder workshop | £21.71 |
| HbA1c | Haematology | £3 | (53) | 1.09 | 1 | Stakeholder workshop | £3.27 |
| Lipids | Chemistry | £1 | (53) | 1.09 | 1 | Stakeholder workshop | £1.09 |
| Liver function | Chemistry | £1 | (53) | 1.09 | 1 | Stakeholder workshop | £1.09 |
| B12 | Chemistry | £1 | (53) | 1.09 | 1 | Stakeholder workshop | £1.09 |
|  | | | | | | | £82.02 |

The cost of diabetes in the year after diagnosis is assumed to be greater than subsequent years because the individual will receive more contact time whilst their diabetes is being controlled. The additional costs of diabetes in the year after diagnosis are reported in Table 31.

Table 31: Drug costs and resource utilisation costs for the first year after diabetes diagnosis

| Resource | Assumption for costs | Unit cost | Source | Inflation | Annual utilisation | Source | Cost per year |
| --- | --- | --- | --- | --- | --- | --- | --- |
| Nurse at GP | Nurse advanced with qualifications 30 mins | £22 | (52) | 1 | 2 | Stakeholder workshop | £44 |
| Health care assistant | Clinical support worker patient work 10 mins | £5.50 | (52) | 1 | 2 | Stakeholder workshop | £11.00 |
| Urine sample | Biochemistry | £1 | (48) | 1.09 | 2 | Stakeholder workshop | £2.18 |
| B12 | Chemistry | £1 | (53) | 1.09 | 2 | Stakeholder workshop | £2.18 |
| HbA1c | Haematology | £3 | (53) | 1.09 | 2 | Stakeholder workshop | £6.54 |
| Lipids | Chemistry | £1 | (53) | 1.09 | 2 | Stakeholder workshop | £2.18 |
| Liver function | Chemistry | £1 | (53) | 1.09 | 2 | Stakeholder workshop | £2.18 |
| Smoking Cessation | Nicotine replacement therapy | £128 | (52) | 1 | 0.3* | Stakeholder workshop | £38.40 |
|  | | | | | | | £108.66 |
| * Assumed 20% smoking prevalence and 50% uptake of smoking cessation services | | | | | | |  |

### Metformin plus Gliptins

Simulated individuals experience an annual increase in HbA1c. Gillett et al. (2012) assume that individuals switch to dual treatment if HbA1c increases above 7.4% (58). Within the model, the individual is switched to a dual treatment in the first annual cycle in which HbA1c exceeds 7.4%. For costing purposes the second drug to be added to Metformin was Sitagliptin, which is reported in the British National Formulary to cost £1.19 per day (54). Belsey et al. (2009) report that 48% of patients used monitoring strips at a mean weekly consumption of 3.3 (57). Table 32 reports the other resource use costs and utilisation assumptions for diabetics receiving Metformin plus Gliptins.

Table 32: Drug costs and resource utilisation costs for Metformin and Gliptins

| Resource | Assumption for costs | Unit cost | Source | Inflation | Annual utilisation | Source | Cost per year |
| --- | --- | --- | --- | --- | --- | --- | --- |
| Sitagliptin | 100mg per day | £1.19 | (54) | 1 | 365 | Assumption | £433.57 |
| Metformin | 500mg *bid* standard (85% of patients) or modified release (15%) tablets | £25.19 per annum | (54) | 1 | 1 | Assumption | £25.19 |
| Self-monitoring strips | 50 strip pack Active® | £0.18 | (54) | 1 | 82.20 | (57) | £14.78 |
| Nurse at GP | Nurse advanced per surgery consultation with qualifications | £22 | (52) | 1 | 1 | Stakeholder workshop | £22 |
| Health care assistant | Clinical support worker patient work 10 mins | £5.50 | (52) | 1 | 1 | Stakeholder workshop | £5.50 |
| Urine sample | Biochemistry | £1 | (48) | 1.09 | 1 | Stakeholder workshop | £1.09 |
| Eye screening | Optometrist test 2006 price | £21.71 | (56) | 1 | 1 | Stakeholder workshop | £21.71 |
| HbA1c | Haematology | £3 | (53) | 1.09 | 1 | Stakeholder workshop | £3.27 |
| Lipids | Chemistry | £1 | (53) | 1.09 | 1 | Stakeholder workshop | £1.09 |
| Liver function | Chemistry | £1 | (53) | 1.09 | 1 | Stakeholder workshop | £1.09 |
| B12 | Chemistry | £1 | (53) | 1.09 | 1 | Stakeholder workshop | £1.09 |
|  | | | | | | | £530.37 |

### Insulin plus Oral Anti-diabetics

The second major treatment change is assumed to be initiation of insulin. Gillett et al. (2012) assumed that individuals switch to insulin if HbA1c increases above 8.5% (58). Within the model the individual is switched to insulin in the first annual cycle at which HbA1c exceeds 8.5%. The insulin Glargine was chosen to represent insulin treatment in the UK and is consistent with Gillett et al. (2012) (58). The total resource use and costs of this health state are reported in Table 33 & Table 34..

Table 33: Costs of insulin treatment

|  | Price | Source |
| --- | --- | --- |
| Glargine | £628.44 | (59) (2006 prices) |
| Oral anti-diabetics | £43.68 | (59) (2006 prices) |
| Reagent test strips | £221.43 | (59) (2006 prices) |
| Hypoglycaemic rescue | £23.43 | (59) (2006 prices) |
| Pen delivery devices | £54.79 | (59) (2006 prices) |
| Sharps | £68.82 | (59) (2006 prices) |
| Total cost per year | £1,013.51 | 2006 |
| Inflated cost | £1,328.02 | 2020 |

Table 34: Drug costs and resource utilisation costs for insulin and oral anti-diabetics

| Resource | Assumption for costs | Unit cost | Source | Inflation (2013) | Annual utilisation | Source | Cost per year |
| --- | --- | --- | --- | --- | --- | --- | --- |
| Insulin treatment costs | Total annual cost | £1,013.51 | (59) | 1.31 | NA | N/A | £1328 |
| Nurse at GP | Nurse advanced per surgery consultation with qualifications | £22 | (52) | 1 | 3 | Stakeholder workshop | £66 |
| Health care assistant | Clinical support worker patient work 10 mins | £5.50 | (52) | 1 | 3 | Stakeholder workshop | £16.50 |
| Urine sample | Biochemistry | £1 | (53) | 1.09 | 3 | Stakeholder workshop | £3.27 |
| Eye screening | Optometrist test 2006 price | £21.71 | (56) | 1 | 1 | Stakeholder workshop | £21.71 |
| HbA1c | Haematology | £3 | (53) | 1.09 | 3 | Stakeholder workshop | £9.81 |
| Lipids | Chemistry | £1 | (53) | 1.09 | 3 | Stakeholder workshop | £3.27 |
| Liver function | Chemistry | £1 | (53) | 1.09 | 3 | Stakeholder workshop | £3.27 |
| B12 | Chemistry | £1 | (53) | 1.09 | 3 | Stakeholder workshop | £3.27 |
|  | | | | | | | £1503 |

## Statins

We assumed that individuals who are prescribed statins receive a daily dose of 40mg of generic Simvastatin. The British National Formulary reports a cost of approximately 7p per day (54). The individual remains on statins for the rest of their life. Table 35 reports the derived annual costs for statins. We assumed that individual’s cholesterol is monitored whilst on statins and patients receive two lipid tests per year. The cost of GP attendance was not included in the cost of statins to avoid double counting of GP attendance.

Table 35: Annual treatment costs of statins

|  | Assumption for costs | Unit cost | Source | Inflation | Annual utilisation | Cost per year |
| --- | --- | --- | --- | --- | --- | --- |
| Statins | Simvastatin 20mg | £0.068 | (54) | 1 | 360 | £25.03 |
| Statins | Lipid tests | £1 | (53) | 1.09 | 2 | £2.18 |
|  |  |  |  |  |  | £27.21 |

## Anti-hypertensives

A search of the literature did not identify any recent publications of anti-hypertensive prescriptions in the UK. As a consequence the best estimates of cost of anti-hypertensive treatment dated from 2004. These were inflated to current prices (52). Due to the number of different anti-hypertensive treatments available and possibilities for combination therapies, using the cost from this study of prescriptions was preferred to using costs directly from the BNF.

Table 36: Annual cost of anti-hypertensive prescription expenditure per patient

|  | Price | Inflation | Cost per year | Standard error | Source |
| --- | --- | --- | --- | --- | --- |
| Anti-hypertensive prescriptions | £144 | 1.359 | £195.66 | 19.57 | (60) |

## Cardiovascular Events

Costs for coronary heart disease disease were obtained from a 2009 HTA for high dose lipid-lowering therapy unless otherwise stated (16). The costs of stroke and fatal CVD were obtained from a 2016 study (61). The costs of congestive heart failure were estimated from the UKPDS costing study for complications related to diabetes (62). Table 37 describes the costs and resource use assumptions that were used for this study. It also reports the health states to which we have applied each cost in the model.

Table 37: Resources use assumptions and costs for cardiovascular outcomes

|  | Resource assumptions | Cost (2020) | Health States applied |
| --- | --- | --- | --- |
| Unstable Angina year 1 | Secondary care costs: 100% hospitalisation, 50% revascularisation procedure, three outpatient appointments).  Primary care costs (three GP visits) and medications. | £3,456 | UANG1 |
| MI year 1 | Secondary care costs: 100% hospitalisation,  50% revascularisation procedure, three outpatient appointments).  Primary care costs (three GP visits) and medications. | £3,866 | MI1 |
| Subsequent ACS care costs | Secondary care costs (one outpatient appointment).  Primary care costs (three GP visits) and medications. | £327 | SANG, UANG, MI |
| Stroke year 1 | Costs of first year post stroke (61) | £9,643 | STRO1 |
| Stroke subsequent costs | Average costs in years 2-5 following stroke (61) | £2,077 | STRO2 |
| Transient Ischemic Attack | Hopsital costs from 5 year study | £327 | TIA |
| Fatal CHD | Walker et al. 2016 (61) | £2,400 |  |
| Fatal non cardio- vascular event | Walker et al. 2016 (61) | £2,013 |  |
| Congestive heart failure year 1 | UKPDS (62) | £1,964 |  |
| Congestive heart failure subsequent years | UKPDS (62) | £2,787 |  |

## Renal Failure

The cost of renal failure was estimated for the UK using relevant published studies. A recent costing study reported the costs of dialysis types (63). The prevalence of dialysis and transplants were taken from a second study reporting the prevalence of renal failure in the UK in 2008 (64). The cost of renal transplantation was taken from a costing study investigating the cost-effectiveness of renal transplantation (65). The overall cost was estimated as a weighted average of the treatment outcomes. All costs were inflated to 2020 prices.

Table 38: Unit costs for renal failure

|  | Cost (£) | Source | Inflation | Cost (2020) | Standard error | Proportion |
| --- | --- | --- | --- | --- | --- | --- |
| Haemodialysis with overheads | 34,236 | (63) | 1.226 | £42,250 | 4225.0 | 0.469 |
| Automated peritoneal dialysis (APD) | 22,160 | (63) | 1. 226 | £26,547 | 2654.7 | 0.045* |
| Continuous ambulatory peritoneal dialysis (CAPD) | 16,074 | (63) | 1. 226 | £19,087 | 1908.7 | 0.045* |
| Transplant | 13,088 | (65) | 1 | £13,088 | 1308.8 | 0.442 |
| Immunosuppressants annual cost | 5000 | (65) | 1. 456 | £7,280 | 728.0 |  |
| * Assumed 50% split of peritoneal dialysis types | | | | | | |

## Foot Ulcers

A search of the literature did not identify any studies for foot ulcer for the UK or a health system comparable to the UK. The cost of foot ulcers was estimated from a US Cost of Illness study (66). We acknowledge that this is a limitation of the analysis, because US costs may not be representative of care in the UK. The costs were converted from dollars to pounds using Purchasing Power Parities reported by the OECD (67). The costs were also inflated to UK 2020 prices.

Table 39: Estimated cost of foot ulcers

| Resource component | Not Infected | With Cellulitis | With Osteomyelitis |
| --- | --- | --- | --- |
| Prevalence | 0.874 | 0.09 | 0.036 |
| Mean cost per patient | $178.97 | $472.73 | $876.52 |
| Mean cost per patient (2020 £) | £197 | £520 | £964 |
| Standard error | 19.7 | 52.0 | 96.4 |
| Total Cost PPP (2020 £) | | | £401.95 |

## Amputation

The cost of amputation in the first year of surgery and subsequent years has been reported in a UKPDS costing study (68). The costs were extracted and inflated to 2020 prices. The cost of amputation in the first year was £10,075 and in subsequent years was £3,877.

## Blindness

The cost of blindness in the first year of surgery and subsequent years has been reported In a UKPDS costing study (68). The costs were extracted and inflated to 2020 prices. The cost of blindness in the first year was £2,226 and in subsequent years was £1,357.

## Cancer

The cost of breast and colorectal cancer is estimated as a one-off fixed cost at diagnosis in the model. This simplifying assumption means that the cost of cancer treatment is independent of survival. We acknowlegde that this assumption will affect the timing of costs because all costs are imposed in the first year and subject to less discounting. However, we anticipate that the impact on overall outcomes will not be substantial. A large proportion of costs are will be incurred in the first year of treatment (surgery, chemotherapy, radiotherapy). Costs in subsequent years will be lower for patients who achieve remission and survival will be short in patients who relapse. Therefore, the costs are likely to be skewed to the early years post diagnosis.

A recent appraisal for cancer screening estimated the overall cost of breast cancer as a weighted average depending on the prognosis at diagnosis as £13,036 when inflated to 2020 prices (69).

The cost of colorectal cancer was taken from a screening appraisal which reported the lifetime costs of colorectal cancer according to the Dukes stage of the tumour (70). The appraisal also reported the proportion of cancers identified at each stage, which allowed us to estimate the weighted average cost of colorectal cancer. Table 40 reports the overall cost of colorectal cancer by stage of disease at diagnosis.

Table 40: Estimated cost of colorectal cancer

| Resource component | Dukes’ Stage A | Dukes’ Stage B | Dukes’ Stage C | Stage D |
| --- | --- | --- | --- | --- |
| Number of patients | 3241.92 | 9,431.04 | 7,662.72 | 8,841.60 |
| Prevalence | 0.111 | 0.323 | 0.263 | 0.303 |
| Mean cost per patient | £7,250.84 | £12,441.41 | £19,076.90 | £11,945.78 |
| Price Inflation | | | | 1.409 |
| Mean cost per patient (2020) | £11,694 | £17,530 | £26,880 | £16,831 |
| Total Cost (2020) | | | | £19,126 |

## Osteoarthritis

The annual cost of osteoarthritis were estimated in a report in 2010 (71). In this report the authors estimated the expected cost of osteoarthritis from three previous costing studies. The costs include GP attendance, nurse consultations, replacement surgery, help at home and prescription medications. The estimated annual cost of osteoarthritis was £783 in 2008. In the study 93% of the costs were attributable to direct medical costs and 7% to social care. Therefore, cost of direct medical care in 2020 prices was £895.

## Depression

Depression is modelled as a chronically recurrent disorder, with patients experiencing further depressive episodes after remission. In the model it is assumed that patients continue to incur costs of depression following an initial diagnosis. These costs reflect ongoing resource use to deal with relapse and prevention of relapse.

A recent trial to prevent secondary depressive episodes collected comprehensive cost data from a sample of individuals with depression (72). The resource uses identified in the control arm were extracted to estimate the costs of depression. The costs from this data (inflated to 2020 prices) were not implemented directly into the SPHR diabetes prevention model as this would have over-estimated the number of GP visits. The model already accounts for GP attendance due to depression. Therefore, a revised estimate of the cost of depression, excluding GP consultation was estimated using updated unit costs. The resource use estimates and revised unit cost estimates used to generate a cost of depression excluding GP utilisation are reported in Table 41.

Table 41: Depression utilisation of services and total estimated cost

|  | Assumption for costs | Unit cost | Source | Inflation | Annual utilisation | Source | Cost per year |
| --- | --- | --- | --- | --- | --- | --- | --- |
| Practice nurse at surgery | GP nurse face to face assume 10 mins | £7.33 | (52) | 1 | 1.52 | (72) | £11.15 |
| Practice nurse at home visit | GP nurse face to face assume 30 mins | £22.00 | (52) | 1 | 0.02 | (72) | £0.44 |
| Practice nurse telephone | GP nurse face to face assume 10 mins | £7.33 | (52) | 1 | 0.11 | (72) | £0.81 |
| Health visitor | Health visitor per hour visit 30 mins | £22.00 | (52) | 1 | 0.05 | (72) | £1.10 |
| District nurse | Community nurse 30 mins | £22.00 | (52) | 1 | 0.01 | (72) | £0.22 |
| Other nurse | GP nurse face to face assume 10 mins | £7.33 | (52) | 1 | 0.13 | (72) | £0.95 |
| HCA phlebotomist | Clinical support worker 10 mins | £2.17 | (72) | 1.0219 | 0.31 | (72) | £0.82 |
| Other primary care | Advanced nurse with qualifications | £14.48 | (72) | 1.0219 | 0.19 | (72) | £3.35 |
| Out of hours | Inflated of trial costs | £5.13 | (72) | 1.0219 | 0.23 | (72) | £1.44 |
| NHS direct | Inflated of trial costs | £1.89 | (72) | 1.0219 | 0.09 | (72) | £0.21 |
| Walk-in centre | Inflated of trial costs | £6.77 | (72) | 1.0219 | 0.21 | (72) | £1.73 |
| Prescribed medications | Inflated of trial costs | £61.87 | (72) | 1.0219 | 7.74 | (72) | £583.55 |
| Secondary care | Emergency Medicine, Any Investigation | £23.85 | (72) | 1.0219 | 0.26 | (72) | £7.56 |
|  | | | | | | | £613.33 |

## Dementia

### Cost of Diagnosis

A one off cost of diagnosis is incurred in the first year of the disease to account for the costs associated with assessing and diagnosing patients. The most recent cost study of Dementia for the UK estimated the cost of Dementia diagnosis at £650 in 2012/13 prices (47) inflated to £740.61.

### Ongoing healthcare costs

The direct health care costs of dementia to the NHS were estimated in an Alzheimers UK report in 2014. The costs were estimated from a modelling study based on PSSRU aggregeate long term care model and PSSRU dementia care model. The report describes costs of care for patients with dementia in 2012 £. Full details of the costing model are reported elsewhere. Table 42 reports the costs of dementia for individuals in community or residential care according to MMSE cognitive score. In the model it is assumed that healthcare costs are met entirely by the NHS. These costs are applied in the model to patients with a dementia diagnosis on an annual basis.

Table 42: Average annual direct healthcare dementia costs

|  | Healthcare costs | | Proportion of patients residential care | Total cost | Total Costs 2020 £ |
| --- | --- | --- | --- | --- | --- |
|  | Community | Residential |  |  |  |
| Mild (MMSE 21-26) | 2,751 | 4,504 | 10.4% | 2932 | 3341 |
| Moderate (MMSE 10-20) | 2,695 | 9,438 | 76.2% | 7837 | 8929 |
| Severe (MMSE 0-9) | 11,258 | 8,689 | 76.2% | 9300 | 10,596 |

# Social Care costs

In this analysis the social care costs refer to the public and private costs incurred with social care as a consequence of a diagnosis with osteoarthritis, stroke or dementia. Social care costs associated with the other health outcomes of the model are not included in this estimate. This is likely to under-estimate the overall cost of social care in the population. However, reliable social care costs for other conditions are very hard to obtain because they are less commonly incurred in the prevalent patient population and more likely to be attributed to other factors or ageing more generally.

**Osteoarthritis**

The annual cost of osteoarthritis were estimated in a report in 2010 (71). The estimated annual cost of osteoarthritis was £783 in 2008. In the study 93% of the costs were attributable to direct medical costs and 7% to social care. Therefore, social care costs in 2020 prices was £65.

## Stroke

The community costs in the first year following stroke were estimated from the South London Stroke Register (73). The average number of days at day centres, nursing homes, residential home, sheltered accommodation and were used to estimate the social care costs.

|  | Mean number of days | Source | Unit cost per day | Source | Total cost |
| --- | --- | --- | --- | --- | --- |
| Day Centre | 3.9 | (73) | £59 | (52) | 254.38 |
| Nursing Home | 16.9 | (73) | £75 | (52) | 1,265.09 |
| Residential Home | 8.5 | (73) | £101 | (52) | 857.29 |
| Sheltered Home | 8.1 | (73) | £65 | (52) | 526.50 |
| Total cost | | | | | 2878.97 |

## Dementia

The social care costs of dementia were estimated in an Alzheimers UK report in 2014 (47). The costs were estimated from a modelling study based on PSSRU aggregeate long term care model and PSSRU dementia care model. The report describes costs of care for patients with dementia in 2013 £. Table 42 reports the costs of dementia for individuals in community or residential care according to MMSE cognitive score. We used estimates from the Alzheimers UK report to estimate the pubic and private social care costs of dementia in line with the methods used in this report. We do not include the productivity costs of informal carers or other public costs in the model. These costs are applied in the model to patients with a dementia diagnosis on an annual basis.

Table 43: Average annual dementia costs

|  | Healthcare costs | | Proportion of patients residential care | Total cost | Total Costs 2020 £ |
| --- | --- | --- | --- | --- | --- |
|  | Community | Residential |  |  |  |
| Mild (MMSE 21-26) | 3,121 | 24,737 | 10.4% | 5362 | 6109 |
| Moderate (MMSE 10-20) | 7,772 | 25,715 | 76.2% | 21455 | 24446 |
| Severe (MMSE 0-9) | 10,321 | 25,874 | 76.2% | 22176 | 25267 |

# Utilities

## Baseline Utility

Baseline utilities for all individuals in the cohort were extracted from the HSE 2011. The tariffs for the responses to the 3 level EQ-5D were derived from a UK population study (74). Utility was assumed to decline due to ageing independent of health status. In the simulation, utility declines by an absolute decrement of 0.004 per year. This estimate is based on previous HTA modelling in cardiovascular disease (11).

## Utility Decrements

The utility decrements for long term chronic conditions were applied to the age adjusted EQ-5D score. In consultation with stakeholders, we assumed that a diagnosis of diabetes was not associated with a reduction in EQ-5D independent of the utility decrements associated with complications, comorbidities or depression. Cardiovascular disease, renal failure, amputation, foot ulcers, blindness, cancer, osteoarthritis and depression were all assumed to result in utility decrements. The utility decrements are measured as a factor which is applied to the individual’s age adjusted baseline. If individuals have multiple chronic conditions the utility decrements are multiplied together to give the individual’s overall utility decrement from comorbidities and complications, in line with current NICE guidelines for combining comorbidities (75).

Due to the number of health states it was not practical to conduct a systematic review to identify utility decrements for all health states. A pragmatic approach was taken to search for health states within existing health technology assessments for the relevant disease area or by considering studies used in previous economic models for diabetes prevention. Discussions with experts in health economic modeling were also used to identify prominent sources of data for health state utilities.

Two sources of data were identified for diabetes related complications. A study from the UKPDS estimated the impact of changes in health states from a longitudinal cohort (76). They estimated the impact of myocardial infarction, ischaemic heart disease, stroke, heart failure, amputation and blindness on quality of life using seven rounds of EQ-5D questionnaires administered between 1997 and 2007. This data was used to estimate the utility decrement for amputation and congestive heart failure. The absolute decrement for amputation was converted into utility decrement factors that could be multiplied by the individuals’ current EQ-5D to estimate the relative effect of the complication. Blindness was included in the statistical model used for this analysis however the UKPDS analysis reported an increase in health state utility following a diagnosis with blindness. Discussions with the authors highlighted that this was due to treatment following formal classification with blindness and it was decided that this increase in health state utility should not be included in the cost-effectiveness model.

Utility decrements for renal failure and foot ulcers were not available from the UKPDS study described above. A study by Coffey et al. (2000) was used to estimate utility decrements for renal failure and foot ulcers (77). In this study, 2,048 subjects with type 1 and type 2 diabetes were recruited from specialty clinics. The Self-Administered Quality of Well Being index (QWB-SA) was used to calculate a health utility score.

A meta-analysis of utility values for diabetes and diabetes related complications estimated utility decrements for amputation, ulcer, end stage renal failure and blindness (78). The study pooled utility measures using different health state valuation measures in a meta-analysis. Pooling health state utility values is problematic because of the fact that different valuation methods and different preference-based measures (PBMs) can generate different values on exactly the same clinical health state (54).There were not sufficient studies in the meta-analysis to adjust for the effects of health state valuation measure on the result. This is a limitation of the analysis and we decided that it was preferable to use estimates from single studies.

Utility decrements for cardiovascular events were taken from an HTA assessing statins to reflect the utility decrements in all patients (7) rather than using the UKPDS, which is only representative of a diabetic population. The study conducted a literature review to identify appropriate utility multipliers for stable angina, unstable angina, myocardial infarction and stoke. We used these estimates in the model and assume that transient ischaemic attack is not associated with a utility decrement in line with this HTA.

We identified a systematic review of breast cancer utility studies following consultation with colleagues with experience in this area. The review highlighted a single burden of illness study with a broad utility decrement for cancer (79), rather than utilities by cancer type or disease status. This study was most compatible with the structure of the cost-effectiveness structure. Within this study 1823 cancer survivors and 5469 age-, sex-, and educational attainment-matched control subjects completed EQ-5D questionnaires to estimate utility with and without cancer.

The utility decrement for osteoarthritis was taken from a Health Technology Assessment that assessed the clinical effectiveness and cost-effectiveness of glucosamine sulphate/hydrochloride and chondroitin sulphate in modifying the progression of osteoarthritis of the knee (80).

A review of cost-effectiveness studies highlights the scarcity of studies of health-related quality of life in depression (81). The utility studies identified in the review described depression states by severity and did not adjust for comorbid conditions. Furthermore, the valuations were variable between studies suggesting poor consistency in the estimations. Therefore, it was difficult to apply these in the model. We decided to use a study which had used the EQ-5D in an RCT, for consistency with our utility measure (82). They report an average post treatment utility of 0.67, from which we estimated the utility decrement compared with the average utility reported in the HSE dataset. The decrement was then converted into a relative utility reduction.

The quality of life impact of dementia is estimated from a study by Jonsson and colleagues (83). These utility values were idenfied and used in the most recent NICE HTA for Alzheimer disease (46). A systematic review of health state utilities for alzheimers disease discusses differences in health related quality of life in different settings (84). It is often assumed that patients in institutional settings will be more disabled and have poorer quality of life. However, the studies that compared utility between settings did not identify a statistically significant difference. Therefore, we only related utility to MMSE.

Table 44 reports the multiplicative utility factors that are used in the model to describe health utility decrements from comorbid complications. The mean absolute decrement estimated in each study is reported alongside the baseline utility for each study. The utility factor was estimated by dividing the implied health utility with the comorbidity by the baseline utility.

Table 44: Utility decrement factors

|  | Mean Absolute decrement | St. error absolute decrement | Baseline Utility | Multiplicative Utility Factor | Source |
| --- | --- | --- | --- | --- | --- |
| Foot ulcer | -0.099 | 0.013 | 0.689 | 0.856 | Coffey (77) |
| Amputation | -0.172 | 0.045 | 0.807 | 0.787 | UKPDS (76) |
| Blind |  |  |  | 1.00 | Assumption |
| Renal failure | -0.078 | 0.026 | 0.689 | 0.887 | Coffey (77) |
| Stable Angina |  |  |  | 0.801 | Ward HTA (11) |
| Unstable Angina y1 |  |  |  | 0.770 | Ward HTA (11) |
| Unstable Angina y2 |  |  |  | 0.770 | Ward HTA (11) |
| Myocardial Infarction y1 |  |  |  | 0.760 | Ward HTA (11) |
| Myocardial Infarction y2 |  |  |  | 0.760 | Ward HTA (11) |
| Transient Ischaemic Attack |  |  |  | 1.000 | Ward HTA (11) |
| Stroke y1 |  |  |  | 0.629 | Ward HTA (11) |
| Stroke y2 |  |  |  | 0.629 | Ward HTA (11) |
| Breast Cancer | -0.060 | 0.008 | 0.791 | 0.913 | Yabroff (79) |
| Colorectal Cancer | -0.060 | 0.008 | 0.791 | 0.913 | Yabroff (79) |
| Osteoarthritis | -0.101 | 0.069 | 0.791 |  | Black HTA (80) |
| Depression | -0.116 |  | 0.791 | 0.875 | Benedict (82) |
| Congestive Heart Failure | -0.101 | 0.032 |  | 0.875 | UKPDS (76) |
| MMSE 26-30 |  |  | 0.690 |  | Jonsson (83) |
| MMSE 21-25 | -0.05 |  | 0.690 | 0.93 | Jonsson (83) |
| MMSE 15-20 | -0.19 |  | 0.690 | 0.725 | Jonsson (83) |
| MMSE 10-14 | -0.20 |  | 0.690 | 0.710 | Jonsson (83) |
| MMSE 0-9 | -0.36 |  | 0.690 | 0.478 | Jonsson (83) |
| UKPDS baseline utility 0.807; HSE baseline 0.7905 | | | | | |

# Intervention Effectiveness

## Intervention Effectiveness

The effectiveness of restrictions on advertising in Greater London were estimated using an interrupted time series analysis of the Kantar Fast Moving Consumer Goods (FMCG) panel from 18^th^ June 2018 to 29^th^ December 2019. The intervention effect on household energy consumption, disaggregated by socioeconomic position is detailed in Table 45.

Table 45: Change in weekly household mean (95% CI) number of packs of HFSS products, energy and nutrients purchased in London compared to the counterfactual, stratified by socioeconomic position

|  | **All (n=1970)** | **High SEP (n=464)** | **Middle SEP (n=1164)** | **Low SEP (n=342)** |
| --- | --- | --- | --- | --- |
| Packs (no.) | **-0.7 (-1.2 to -0.2)** | -0.2 (-1.1 to 0.8) | **-0.8 (-1.5 to -0.2)** | -0.8 (-2.3 to 0.7) |
| Energy (kcal) | **-1,001.0 (-1,546.0 to -456.0)** | -586.6 (-1,632.2 to 458.9) | **-1,139.4 (-1,834.2 to -444.6)** | -875.5 (-2,501.3 to 750.2) |
| Fat (g) | **-57.9 (-93.7 to -22.1)** | -32.2 (-102.5 to 38.1) | **-60.3 (105.3 to -15.2)** | -65.3 (-170.1 to 39.5) |
| Saturated fat (g) | **-26.4 (-40.4 to -12.4)** | -20.2 (-48.2 to 7.7) | **-27.9 (-45.8 to -9.9)** | -25.4 (-64.7 to 13.9) |
| Sugar | **-80.7 (-120.1 to -41.4)** | -75.8 (-155.3 to 3.7) | **-95.3 (-146.2 to -44.4)** | -14.0 (-121.8 to 93.8) |
| Salt | -2.2 (-9.8 to 5.4) | -7.5 (-20.8 to 5.8) | -4.4 (-14.7 to 5.9) | 12.8 (-5.4 to 30.9) |
| **Bold**, significant at 95% confidence level. Weekly household mean purchases estimated from controlled interrupted time series two-part model: part 1 (logit) and part 2 (generalised linear model) with negative binomial distribution for packs and gamma distribution for energy and nutrients. Models adjusted for festivals, season, number of adults in household, number of children in household, and sex, age and socioeconomic position of main food shopper. Cluster-robust standard errors used. Observations where households did not report any food and drink purchases that week dropped. Data period=18 June 2018 to 29 December 2019. | | | | |

Changes in calories have been strongly associated with weight gain. There has been a lot of work over the years developing mathematical models for weight loss and energy expenditure. A rapid review was carried out of dynamic weight change models that have been developed into online tools requiring limited inputs for age, sex, height, body weight and total energy expenditure. NIH BWP and PBRC WLP have been concurrently validated against data from the CALORIE trial (85, 86). The validation study identifies underestimation of weight change measures using the PBRC WLP tool compared with the NIH BWP model (87). Following this publication some discussion has arisen between the authors responsible for the development of both models. It is clear that there may be some discrepancy between how PBRC WLP was programmed in the validation study and the actual model. However, there is currently ambiguity on how this model should be programmed. Therefore, I would suggest that the NIH BWP model would be preferred at this time unless the issue with PBRC WLP is resolved.

We have identified 3 economic evaluations of dietary public health policies and interventions in which the Hall et al. equations have been used to describe the relationship between calories and weight change (88-90). Whilst some evaluations have programmed the dynamic weight change equations (88), others have used the equations to approximate the model (89). This method has been adopted in an Australian model for traffic light labelling and junk food taxes (91).

Method

In the Dietary Change Model weight change is conditional on changes in calories, and the relationship is based on the Hall et al. equations. For the method to be compatible with the structures, inputs, and computational demands of the SPHR Diabetes Prevention model we approximate the Hall et al. equations. We extracted a dataset of 1000 outputs from the National Institute for Diabetes and Digestive and Kidney Diseases (NIDDIK) online Body Weight Planner tool to estimate the impact of changes in calories, % carbohydrate and sodium after 12 months of a sustained dietary change. The baseline weight, age, sex, change in calories, change in % carbohydrate, and changes in sodium were all varied across these estimates in order to observe their impact on changes in weight. We developed an ordinary least squares regression model specification to describe the changes calories, percentage carbohydrate, sodium and how these are modified by baseline weight, age and gender. Changes to physical activity and height were held constant in the online tool, because these were not likely to be modifiable used to describe heterogeneity in effects in the intended application of the approximation. Goodness of fit was assessed using the R^2^ an Akaike Information Criterion. The final model specification is detailed in equation 1

$$\Delta weight= \beta_{0}+\beta_{1}{weight}_{0}+\beta_{2}age+\beta_{3}male+\beta_{4}\Delta calorie+\beta_{5}\Delta calorie*{weight}_{0}+\beta_{5}\Delta calorie*age+\beta_{6}\Delta calorie*male+\beta_{7}\Delta\%carb+ \beta_{8}\Delta sodium+\varepsilon$$

Results

The final coefficient estimates are reported in Table 46. All coefficients were statistically significantly associated with changes in weight. Changes in calories were highly associated with weight change, and this variable described a large proportion of the variation in weight outcomes. Weight at baseline, and males were positively associated with changes in weight, but the effects were relatively small. However, interaction terms suggest that the impact of changes in calories increased with age and baseline weight and decreased for males. Weight increased if the proportion of diet supplied by carbohydrates increased and increased if sodium intake increased.

Table 46: Regression coefficients for the weight change model for adults

|  | Mean | Standard error | 2.5^th^ CI | 97.5^th^ CI |
| --- | --- | --- | --- | --- |
| 1. Constant | -0.040899 | 0.0196478 | -0.0794549 | -0.0023437 |
| 2. Weight at baseline | 0.000005 | 0.0001846 | -0.000357 | 0.0003674 |
| 3. Age | 0.000200 | 0.0002212 | -0.0002337 | 0.0006343 |
| 4. Male | 0.012742 | 0.0088474 | -0.0046195 | 0.0301036 |
| 5. Change in calories | 0.024161 | 0.0001778 | 0.023812 | 0.0245098 |
| 6. Change in calories by baseline weight | 0.000012 | 1.70e-06 | 8.91e-06 | 0.0000156 |
| 7. Change in calories by age | 0.000021 | 2.06e-06 | 0.0000171 | 0.0000251 |
| 8. Change in calories by male | -0.000941 | 0.000082 | -0.0011018 | -0.0007798 |
| 9. Changes in % carbohydrate | 0.033863 | 0.0004127 | 0.0330526 | 0.0346724 |
| 10. Changes in sodium | 0.000243 | 0.0000206 | 0.0002024 | 0.0002832 |

The R^2^ was over 99% suggesting that most of the variability in changes in weight were explained by the model specification. Figure 1 illustrates a scatter plot of the weight change outcomes predicted using the Hall et al. online tool compared with the regression model approximation. The graph confirms the results from the R^2^ statistic that the regression approximation explains most of the variation in the predicted weight change. Table 46

Figure 4: Predicted values from the regression approximation and a comparison with estimates from the NIDDIK online tool

Table 47 Mean (SD) change in weight using the regression approximation method and comparison with estimates from the Hall et al. mathematical model

| Calorie changes | Hall et al. mathematical model prediction | Regression approximation mean (SD) |
| --- | --- | --- |
| -34 kcal | -0.9kg | -0.89kg (0.03) |
| -51 kcal | -1.3kg | -1.34kg (0.04) |
| -54 kcal | -1.4kg | -1.41kg (0.04) |
| -62 kcal | -1.6kg | -1.61kg (0.04) |

## Duration of Intervention Effect

In the base case analysis the change in calories was assumed to last for 12 months. After this no difference in calories were assumed to be consumed between the intervention arm and control. The NIDDIK Body Planner Online tool was used to generate the average time for weight to return to an individual’s natural history trajectory, and this was estimated to be 4 years in equal linear increments.

# Probabilistic Sensitivity Analysis

Probabilistic sensitivity analysis (PSA) was enabled in the model to describe the uncertainty in parameter inputs of the model and how this translates into uncertainty in the outcomes of the model. A suitable distribution was selected for each parameter, based upon its mean and standard error. Random sampling simultaneously across all input parameter distributions allowed parameter uncertainty to be quantified. 2000 different random samples of parameter values were selected, and each was applied to a simulated cohort of 20000 individuals meeting the eligibility criteria. For each PSA sample, the model was run and results compiled. Given the large number of parameters in the model and thus the capacity for error, a thorough process of checking that mean sampling values corresponded to mean parameter values was undertaken to ensure that the results were as accurate as possible.

# Model Validation

The SPHR model has undergone a thorough process of error checking and internal and external validations. Validation of the model to predict metabolic data, diabetes and cardiovascular disease have been reported elsewhere.

# References

1. Squires H, Chilcott J, Akehurst R, Burr J, Kelly MP. A Framework for Developing the Structure of Public Health Economic Models. Value Health. 2016;19(5):588-601.

2. Excellence NIfHaC. PH35: Preventing type 2 diabetes: population and community-level interventions. National Institute for Health and Care Excellence [Internet]. 2011; NICE public health guidance 35. Available from: <https://www.nice.org.uk/guidance/ph35>.

3. Excellence NIfHaC. PH38 Preventing type 2 diabetes - risk identification and interventions for individuals at high risk: guidance. National Institute for Health and Care Excellence [Internet]. 2012; NICE public health guidance 38. Available from: <http://guidance.nice.org.uk/PH38/Guidance/pdf/English>.

4. Research NS. Health Survey for England. University College London Department of Epidemiology and Public Health [Internet]. 2014. Available from: <http://www.esds.ac.uk/findingData/hseTitles.asp>.

5. Mortality Statistics: Deaths registered in England and Wales (Series DR), 2014. Office of National Statistics [Internet]. 2017. Available from: <http://webarchive.nationalarchives.gov.uk/20160105160709/http://www.ons.gov.uk/ons/publications/re-reference-tables.html?edition=tcm%3A77-378961>.

6. Breeze P, Squires H, Chilcott J, Stride C, Diggle PJ, Brunner E, et al. A statistical model to describe longitudinal and correlated metabolic risk factors: the Whitehall II prospective study. J Public Health (Oxf). 2015.

7. Lomax N, Norman G. Estimating population attribute values in a table: "Get me started in" iterative proportional fitting. The Professional Geographer. 2015;68(3).

8. 2011 Census: Office for National Statistics; 2012 [Available from: <https://www.ons.gov.uk/census/2011census>.

9. English Indices of Deprivation, 2015: Ministry of Housing, Communities & Local Government; 2015 [Available from: <https://www.gov.uk/government/statistics/english-indices-of-deprivation-2015>.

10. Estimates of the population for the UK, England and Wales, Scotland and Northern Ireland Office for National Statistics; 2021 [Available from: <https://www.ons.gov.uk/peoplepopulationandcommunity/populationandmigration/populationestimates/datasets/populationestimatesforukenglandandwalesscotlandandnorthernireland>.

11. Ward S, Lloyd JM, Pandor A, Holmes M, Ara R, Ryan A, et al. A systematic review and economic evaluation of statins for the prevention of coronary events. Health Technol Assess. 2007;11(14):1-iv.

12. Green MA, Li J, Relton C, Strong M, Kearns B, Wu M, et al. Cohort Profile: The Yorkshire Health Study. Int J Epidemiol. 2014:dyu121.

13. Rabe-Hesketh S, Skrondal A. Multilevel and Longitudinal Modelling Using Stata. Second edition ed. College Station: StataCorp; 2008 2008.

14. Arbeev KG, Ukraintseva SV, Akushevich I, Kulminski AM, Arbeeva LS, Akushevich L, et al. Age trajectories of physiological indices in relation to healthy life course. Mech Ageing Dev. 2011;132(3):93-102.

15. Clarke PM, Gray AM, Briggs A, Farmer AJ, Fenn P, Stevens RJ, et al. A model to estimate the lifetime health outcomes of patients with type 2 diabetes: the United Kingdom Prospective Diabetes Study (UKPDS) Outcomes Model (UKPDS no. 68). Diabetologia. 2004;47(10):1747-59.

16. Ara R, Pandor A, Stevens J, Rees A, Rafia R. Early high-dose lipid-lowering therapy to avoid cardiac events: a systematic review and economic evaluation. Health Technol Assess. 2009;13(34):1-118.

17. Excellence NIfHaC. Hypertension: Clinical management of primary hypertension in adults. 2011 2011. Report No.: CG 127.

18. Wald DS, Law M, Morris JK, Bestwick JP, Wald NJ. Combination therapy versus monotherapy in reducing blood pressure: meta-analysis on 11,000 participants from 42 trials. Am J Med. 2009;122(3):290-300.

19. Excellence NIoHaC. Statins for the prevention of cardiovascular events in patients at increased risk of developing cardiovascular disease or those with established cardiovascular disease. 2006 2006. Report No.: Technology appraisals, TA94.

20. Hippisley-Cox J, Coupland C. Development and validation of QDiabetes-2018 risk prediction algorithm to estimate future risk of type 2 diabetes: cohort study. BMJ. 2017;359:j5019.

21. Watson P, Preston L, Squires H, Chilcott J, Brennan A. Modelling the Economics of Type 2 Diabetes Mellitus Prevention: A Literature Review of Methods. Appl Health Econ Health Policy. 2014;12(3):239-53.

22. Hayes AJ, Leal J, Gray AM, Holman RR, Clarke PM. UKPDS outcomes model 2: a new version of a model to simulate lifetime health outcomes of patients with type 2 diabetes mellitus using data from the 30 year United Kingdom Prospective Diabetes Study: UKPDS 82. Diabetologia. 2013;56(9):1925-33.

23. D'Agostino RB, Sr., Vasan RS, Pencina MJ, Wolf PA, Cobain M, Massaro JM, et al. General cardiovascular risk profile for use in primary care: the Framingham Heart Study. Circulation. 2008;117(6):743-53.

24. Hippisley-Cox J, Coupland C, Vinogradova Y, Robson J, Minhas R, Sheikh A, et al. Predicting cardiovascular risk in England and Wales: prospective derivation and validation of QRISK2. BMJ. 2008;336(7659):1475-82.

25. McEwan P, Bennett H, Ward T, Bergenheim K. Refitting of the UKPDS 68 risk equations to contemporary routine clinical practice data in the UK. Pharmacoeconomics. 2015;33(2):149-61.

26. ClinRisk. QResearch [Internet]. 2019. Available from: <http://www.qrisk.org/>.

27. Ahern AL, Wheeler GM, Aveyard P, Boyland EJ, Halford JCG, Mander AP, et al. Extended and standard duration weight-loss programme referrals for adults in primary care (WRAP): a randomised controlled trial. The Lancet. 2017;389(10085):2214-25.

28. Hippisley-Cox J, Coupland C, Robson J, Brindle P. Derivation, validation, and evaluation of a new QRISK model to estimate lifetime risk of cardiovascular disease: cohort study using QResearch database. BMJ. 2010;341:c6624. doi: 10.1136/bmj.c6624.:c6624.

29. Khaw KT, Wareham N, Luben R, Bingham S, Oakes S, Welch A, et al. Glycated haemoglobin, diabetes, and mortality in men in Norfolk cohort of european prospective investigation of cancer and nutrition (EPIC-Norfolk). BMJ. 2001;322(7277):15-8.

30. Kannel WB, D'Agostino RB, Silbershatz H, Belanger AJ, Wilson PW, Levy D. Profile for estimating risk of heart failure. Arch Intern Med. 1999;159(11):1197-204.

31. Kaffashian S, Dugravot A, Brunner EJ, Sabia S, Ankri J, Kivimaki M, et al. Midlife stroke risk and cognitive decline: a 10-year follow-up of the Whitehall II cohort study. Alzheimers Dement. 2013;9(5):572-9.

32. Johansen NB, Vistisen D, Brunner EJ, Tabak AG, Shipley MJ, Wilkinson IB, et al. Determinants of aortic stiffness: 16-year follow-up of the Whitehall II study. PLoS One. 2012;7(5):e37165.

33. Dadvand P, Rankin J, Shirley MD, Rushton S, Pless-Mulloli T. Descriptive epidemiology of congenital heart disease in Northern England. Paediatr Perinat Epidemiol. 2009;23(1):58-65.

34. Davies M, Hobbs F, Davis R, Kenkre J, Roalfe AK, Hare R, et al. Prevalence of left-ventricular systolic dysfunction and heart failure in the Echocardiographic Heart of England Screening study: a population based study. Lancet. 2001;358(9280):439-44.

35. Lahmann PH, Hoffmann K, Allen N, van Gils CH, Khaw KT, Tehard B, et al. Body size and breast cancer risk: findings from the European Prospective Investigation into Cancer And Nutrition (EPIC). Int J Cancer. 2004;111(5):762-71.

36. Pischon T, Lahmann PH, Boeing H, Friedenreich C, Norat T, Tjonneland A, et al. Body size and risk of colon and rectal cancer in the European Prospective Investigation Into Cancer and Nutrition (EPIC). J Natl Cancer Inst. 2006;98(13):920-31.

37. Renehan AG, Tyson M, Egger M, Heller RF, Zwahlen M. Body-mass index and incidence of cancer: a systematic review and meta-analysis of prospective observational studies. Lancet. 2008;371(9612):569-78.

38. Schett G, Kleyer A, Perricone C, Sahinbegovic E, Iagnocco A, Zwerina J, et al. Diabetes is an independent predictor for severe osteoarthritis: results from a longitudinal cohort study. Diabetes Care. 2013;36(2):403-9.

39. Palmer AJ, Roze S, Valentine WJ, Minshall ME, Foos V, Lurati FM, et al. The CORE Diabetes Model: Projecting long-term clinical outcomes, costs and cost-effectiveness of interventions in diabetes mellitus (types 1 and 2) to support clinical and reimbursement decision-making. Curr Med Res Opin. 2004;20(Suppl. 1):S5-S26.

40. Golden SH, Lazo M, Carnethon M, Bertoni AG, Schreiner PJ, Diez Roux AV, et al. Examining a bidirectional association between depressive symptoms and diabetes. JAMA. 2008;299(23):2751-9.

41. Whyte EM, Mulsant BH, Vanderbilt J, Dodge HH, Ganguli M. Depression after stroke: a prospective epidemiological study. J Am Geriatr Soc. 2004;52(5):774-8.

42. Walters K, Hardoon S, Petersen I, Iliffe S, Omar RZ, Nazareth I, et al. Predicting dementia risk in primary care: development and validation of the Dementia Risk Score using routinely collected data. Bmc Medicine. 2016;14.

43. Briggs A, Claxton K, Sculfer M. Decision Modelling for Health Economic Evaluation2006 2006.

44. Wimo A, Religa D, Spangberg K, Edlund AK, Winblad B, Eriksdotter M. Costs of diagnosing dementia: results from SveDem, the Swedish Dementia Registry. Int J Geriatr Psychiatry. 2013;28(10):1039-44.

45. Xie J, Brayne C, Matthews FE. Survival times in people with dementia: analysis from population based cohort study with 14 year follow-up. BMJ. 2008;336(7638):258-62.

46. Bond M, Rogers G, Peters J, Anderson R, Hoyle M, Miners A, et al. The effectiveness and cost-effectiveness of donepezil, galantamine, rivastigmine and memantine for the treatment of Alzheimer's disease (review of Technology Appraisal No. 111): a systematic review and economic model. Health Technol Assess. 2012;16(21):1-470.

47. Prince M, Knapp M, Guerchet M, McCrone P, Prina P, Comas-Herrera A, et al. Dementia UK Update. Cognitive Function and Ageing Study [Internet]. 2014. Available from: <http://www.cfas.ac.uk/files/2015/07/P326_AS_Dementia_Report_WEB2.pdf>.

48. Getsios D, Blume S, Ishak KJ, Maclaine GD. Cost effectiveness of donepezil in the treatment of mild to moderate Alzheimer's disease: a UK evaluation using discrete-event simulation. Pharmacoeconomics. 2010;28(5):411-27.

49. Cancer Survival in England: Patients Diagnosed, 2006–2010 and Followed up to 2011. Office of National Statistics [Internet]. 2012. Available from: <http://www.ons.gov.uk/ons/publications/re-reference-tables.html?edition=tcm%3A77-277733>.

50. Seshasai SR, Kaptoge S, Thompson A, Di AE, Gao P, Sarwar N, et al. Diabetes mellitus, fasting glucose, and risk of cause-specific death. N Engl J Med. 2011;364(9):829-41.

51. James BD, Leurgans SE, Hebert LE, Scherr PA, Yaffe K, Bennett DA. Contribution of Alzheimer disease to mortality in the United States. Neurology. 2014;82(12):1045-50.

52. Jones K, Burns A. Unit costs of health and social care. Personal Social Services Research Unit: University of Kent; 2021 2021.

53. NHS reference costs 2019-20. Department of Health [Internet]. 2021. Available from: <https://www.england.nhs.uk/national-cost-collection/>.

54. Peasgood T, Brazier J. Is Meta-Analysis for Utility Values Appropriate Given the Potential Impact Different Elicitation Methods Have on Values? Pharmacoeconomics. 2015;33(11):1101-5.

55. CG127 Hypertension: costing template. National Institute for Care and Clinical Excellence [Internet]. 2011. Available from: <http://guidance.nice.org.uk/CG127/CostingTemplate/xls/English>.

56. GOS sight test fees to rise 1.9% from April 2021: Optometry Today; 2021 [Available from: <https://www.aop.org.uk/ot/professional-support/health-services/2021/03/25/gos-sight-test-fees-to-rise-19-from-april-2021>.

57. Belsey JD, Pittard JB, Rao S, Urdahl H, Jameson K, Dixon T. Self blood glucose monitoring in type 2 diabetes. A financial impact analysis based on UK primary care. Int J Clin Pract. 2009;63(3):439-48.

58. Gillett M, Royle P, Snaith A, Scotland G, Poobalan A, Imamura M, et al. Non-pharmacological interventions to reduce the risk of diabetes in people with impaired glucose regulation: a systematic review and economic evaluation. Health Technol Assess. 2012;16(33):1-iv.

59. Poole C, Tetlow T, McEwan P, Holmes P, Currie C. The prescription cost of managing people with type 1 and type 2 diabetes following initiation of treatment with either insulin glargine or insulin determir in routine general practice in the UK: a retrospective database analysis. Current Medical Research and Opinion. 2007;23(1):S41-S8.

60. Blak BT, Mullins CD, Shaya FT, Simoni-Wastila L, Cooke CE, Weir MR. Prescribing trends and drug budget impact of the ARBs in the UK. Value Health. 2009;12(2):302-8.

61. Walker S, Asaria M, Manca A, Palmer S, Gale CP, Shah AD, et al. Long-term healthcare use and costs in patients with stable coronary artery disease: a population-based cohort using linked health records (CALIBER). Eur Heart J Qual Care Clin Outcomes. 2016;2(2):125-40.

62. Clarke P, Gray A, Legood R, Briggs A, Holman R. The impact of diabetes-related complications on healthcare costs: results from the United Kingdom Prospective Diabetes Study (UKPDS Study No. 65). Diabet Med. 2003;20(6):442-50.

63. Baboolal K, McEwan P, Sondhi S, Spiewanowski P, Wechowski J, Wilson K. The cost of renal dialysis in a UK setting--a multicentre study. Nephrol Dial Transplant. 2008;23(6):1982-9.

64. Byrne C, Steenkamp R, Castledine C, Ansell D, Feehally J. UK Renal Registry 12th Annual Report (December 2009): chapter 4: UK ESRD prevalent rates in 2008: national and centre-specific analyses. Nephron Clin Pract. 2010;115 Suppl 1:c41-67. doi: 10.1159/000301159. Epub@2010 Mar 31.:c41-c67.

65. Cost-effectiveness of transplantation 2013 [updated 2013. Available from: <https://www.organdonation.nhs.uk/newsroom/fact_sheets/organ_donation_registry_fact_sheet_7_21337.pdf>.

66. Gordois A, Scuffham P, Shearer A, Oglesby A, Tobian JA. The health care costs of diabetic peripheral neuropathy in the US. Diabetes Care. 2003;26(6):1790-5.

67. OECD. Purchasing Power Parities (PPPs) for OECD Countries. <http://stats> oecd org/Index aspx?datasetcode=SNA_TABLE4 [Internet]. 2013. Available from: <http://www.oecd.org/>.

68. Alva M, Gray A, Mihaylova B, Leal J, Holman R. The impact of diabetes-related complications on healthcare costs: new results from the UKPDS (UKPDS 84). Diabetic Medicine. 2014:459-66.

69. Madan J, Rawdin A, Stevenson M, Tappenden P. A rapid-response economic evaluation of the UK NHS Cancer Reform Strategy breast cancer screening program extension via a plausible bounds approach. Value Health. 2010;13(2):215-21.

70. Tappenden P, Eggington S, Nixon R, Chilcott J, Sakai H, Karnon J. Colorectal cancer screening options appraisal Report to the English Bowel Cancer Screening Working Group. National Health Service [Internet]. 2004. Available from: <http://www.cancerscreening.nhs.uk/bowel/scharr.pdf>.

71. The economic costs of arthritis for the UK economy. Oxford Economics [Internet]. 2014. Available from: <https://www.oxfordeconomics.com/publication/open/222531>.

72. Chalder M, Wiles NJ, Campbell J, Hollinghurst SP, Searle A, Haase AM, et al. A pragmatic randomised controlled trial to evaluate the cost-effectiveness of a physical activity intervention as a treatment for depression: the treating depression with physical activity (TREAD) trial. Health Technol Assess. 2012;16(10):1-iv.

73. Grieve R, Porsdal V, Hutton J, Wolfe C. A comparison of the cost-effectiveness of stroke care provided in London and Copenhagen. Int J Technol Assess Health Care. 2000;16(2):684-95.

74. Dolan PG, C; Kind, P.;Williams, A. A social tariff for Euroqol: Results from a general population survey. Discussion Paper 138. 1995;University of York.

75. Ara R, Wailoo A. NICE DSU Technical Support Document 12: The use of health state utility values in decision models. 2011 2011.

76. Alva M, Gray A, Mihaylova B, Clarke P. The Effect of Diabetes Complications on Health-Related Quality of Life: The importance of longitudinal data to address patient heterogeneity. Health Econ. 2013:10.

77. Coffey JT, Brandle M, Zhou H, Marriott D, Burke R, Tabaei BP, et al. Valuing health-related quality of life in diabetes. Diabetes Care. 2002;25(12):2238-43.

78. Lung TW, Hayes AJ, Hayen A, Farmer A, Clarke PM. A meta-analysis of health state valuations for people with diabetes: explaining the variation across methods and implications for economic evaluation. Qual Life Res. 2011;20(10):1669-78.

79. Yabroff KR, Lawrence WF, Clauser S, Davis WW, Brown ML. Burden of illness in cancer survivors: findings from a population-based national sample. J Natl Cancer Inst. 2004;96(17):1322-30.

80. Black C, Clar C, Henderson R, MacEachern C, McNamee P, Quayyum Z, et al. The clinical effectiveness of glucosamine and chondroitin supplements in slowing or arresting progression of osteoarthritis of the knee: a systematic review and economic evaluation. Health Technol Assess. 2009;13(52):1-148.

81. Zimovetz EA, Wolowacz SE, Classi PM, Birt J. Methodologies used in cost-effectiveness models for evaluating treatments in major depressive disorder: a systematic review. Cost Eff Resour Alloc. 2012;10(1):1-10.

82. Benedict A, Arellano J, De CE, Baird J. Economic evaluation of duloxetine versus serotonin selective reuptake inhibitors and venlafaxine XR in treating major depressive disorder in Scotland. J Affect Disord. 2010;120(1-3):94-104.

83. Jonsson L, Andreasen N, Kilander L, Soininen H, Waldemar G, Nygaard H, et al. Patient- and proxy-reported utility in Alzheimer disease using the EuroQoL. Alzheimer Dis Assoc Disord. 2006;20(1):49-55.

84. Shearer J, Green C, Ritchie CW, Zajicek JP. Health state values for use in the economic evaluation of treatments for Alzheimer's disease. Drugs Aging. 2012;29(1):31-43.

85. Hall KD, Sacks G, Chandramohan D, Chow CC, Wang YC, Gortmaker SL, et al. Quantification of the effect of energy imbalance on bodyweight. The Lancet. 2011;378(9793):826-37.

86. Thomas DM, Martin CK, Heymsfield S, Redman LM, Schoeller DA, Levine JA. A Simple Model Predicting Individual Weight Change in Humans. J Biol Dyn. 2011;5(6):579-99.

87. Guo J, Brager DC, Hall KD. Simulating long-term human weight-loss dynamics in response to calorie restriction. Am J Clin Nutr. 2018;107(4):558-65.

88. Basu S, Vellakkal S, Agrawal S, Stuckler D, Popkin B, Ebrahim S. Averting obesity and type 2 diabetes in India through sugar-sweetened beverage taxation: an economic-epidemiologic modeling study. PLoS Med. 2014;11(1):e1001582.

89. Lal A, Mantilla-Herrera AM, Veerman L, Backholer K, Sacks G, Moodie M, et al. Modelled health benefits of a sugar-sweetened beverage tax across different socioeconomic groups in Australia: A cost-effectiveness and equity analysis. PLoS Med. 2017;14(6):e1002326.

90. Liu J, Mozaffarian D, Sy S, Lee Y, Wilde PE, Abrahams-Gessel S, et al. Health and Economic Impacts of the National Menu Calorie Labeling Law in the United States: A Microsimulation Study. Circ Cardiovasc Qual Outcomes. 2020;13(6):e006313.

91. Sacks G, Veerman JL, Moodie M, Swinburn B. 'Traffic-light' nutrition labelling and 'junk-food' tax: a modelled comparison of cost-effectiveness for obesity prevention. Int J Obes (Lond). 2011;35(7):1001-9.

1. The model did not converge when BMI slope was included as a predictor for HDL growth. [↑](#footnote-ref-1)
